# The nucleolar protein NOL12 is required for processing of large ribosomal subunit rRNA precursors in Arabidopsis

**DOI:** 10.1186/s12870-023-04561-9

**Published:** 2023-11-03

**Authors:** Monika Zakrzewska-Placzek, Anna Golisz-Mocydlarz, Michal Krzyszton, Justyna Piotrowska, Malgorzata Lichocka, Joanna Kufel

**Affiliations:** 1 Institute of Genetics and Biotechnology, Faculty of Biology, University of Warsaw, Pawinskiego 5a, Warsaw, 02-106 Poland; 2 Laboratory of Seeds Molecular Biology, Institute of Biochemistry and Biophysics, Polish Academy of Sciences, Pawinskiego 5a, Warsaw, 02-106 Poland; 3 Institute of Biochemistry and Biophysics, Polish Academy of Sciences, Pawinskiego 5a, Warsaw, 02-106 Poland

**Keywords:** Arabidopsis thaliana, Nucleolus, pre-rRNA processing, Ribosome biogenesis, rRNA, Stress response

## Abstract

**Background:**

NOL12 5′-3′ exoribonucleases, conserved among eukaryotes, play important roles in pre-rRNA processing, ribosome assembly and export. The most well-described yeast counterpart, Rrp17, is required for maturation of 5.8 and 25S rRNAs, whereas human hNOL12 is crucial for the separation of the large (LSU) and small (SSU) ribosome subunit rRNA precursors.

**Results:**

In this study we demonstrate that plant AtNOL12 is also involved in rRNA biogenesis, specifically in the processing of the LSU rRNA precursor, 27S pre-rRNA. Importantly, the absence of AtNOL12 alters the expression of many ribosomal protein and ribosome biogenesis genes. These changes could potentially exacerbate rRNA biogenesis defects, or, conversely, they might stem from the disturbed ribosome assembly caused by delayed pre-rRNA processing. Moreover, exposure of the *nol12* mutant to stress factors, including heat and pathogen *Pseudomonas syringae*, enhances the observed molecular phenotypes, linking pre-rRNA processing to stress response pathways. The aberrant rRNA processing, dependent on AtNOL12, could impact ribosome function, as suggested by improved mutant resistance to ribosome-targeting antibiotics.

**Conclusion:**

Despite extensive studies, the pre-rRNA processing pathway in plants remains insufficiently characterized. Our investigation reveals the involvement of AtNOL12 in the maturation of rRNA precursors, correlating this process to stress response in Arabidopsis. These findings contribute to a more comprehensive understanding of plant ribosome biogenesis.

**Supplementary Information:**

The online version contains supplementary material available at 10.1186/s12870-023-04561-9.

## Background

In eukaryotic cells, the processing of the long, polycistronic ribosomal RNA precursor (pre-rRNA) involves a series of events that begin co-transcriptionally and occur simultaneously with ribosome assembly to ensure timely and accurate formation of the ribosomes [1, 2]. The primary rRNA transcript (35S in budding yeast, 47S in humans) is synthesized by RNA polymerase I (Pol I) and contains 5.8S, 18S and 25S ribosomal RNAs (rRNAs), as well as external and internal transcribed spacers (5′ETS, 3′ETS, ITS1, ITS2), which are removed during processing in a coordinated manner. The additional 5S rRNA is synthesized independently by the RNA polymerase III (Pol III) [3]. Mature rRNAs are excised from the precursor by endo- and exonucleolytic activities and incorporated into the mature ribosome subunits – SSU (small ribosome subunit, 40S, with 18S rRNA) and LSU (large ribosome subunit; 60S, 25S, 5.8S and 5S rRNAs) which, together with many ribosomal proteins (RPs) form the 80S ribosome [3]. Pre-rRNA processing includes chemical nucleotide modifications, mainly 2′-O-methylation and pseudouridylation, introduced at different stages during ribosome biogenesis, that stabilize the rRNA and ribosome structure [4, 5].

The processing of pre-rRNA takes place mainly in the nucleolus, and nucleoplasm, and the final steps of maturation are carried out after the export of the pre-ribosomes to the cytoplasm [1, 3]. A large number of enzymes and factors are involved in the entire maturation process, many of which have been identified in model organisms, including Arabidopsis, and extensively described in recent reviews [1–3, 6].

The known plant ribosome biogenesis factors (RBFs) include enzymes that directly cleave or trim pre-rRNAs, namely endo- and exoribonucleases, and many other factors that facilitate the processing [1, 6]. In Arabidopsis the nascent rRNA transcript is cleaved by RNase III-type enzyme RTL2 [7, 8]. The resulting pre-rRNA is initially shortened by the 5′-3′ exoribonucleases XRN2 and XRN3 [9], and then endonucleolytically cleaved at the defined site P by the U3 snoRNP complex (NF-D), that contain U3 and U14 snoRNAs, fibrillarin, NOP5/Nop58, Diskerin/Cbf5p and the nucleolin [10, 11], producing the 35S precursor. The resulting 35S pre-rRNA is further matured in two alternative ways (see Supplementary Fig. S1A). In the major processing pathway, the 35S is cleaved within ITS1 segment, which separates the pre-rRNAs destined to the SSU (P-A3 pre-rRNA) and LSU (27S-A3 pre-rRNA). In the alternative minor pathway, the complete removal of 5′ ETS precedes the cleavage at the site A3 within ITS1 [1, 6]. Pre-rRNA processing intermediates resulting from the A3 cleavage, namely the P-A3 and 27S-A3 species, are subsequently subjected to further endonucleolytic processing. The cleavage sites P′ in 5′ETS [12], A2 in ITS1 and C2 in ITS2 [9] have been mapped so far, but the enzymes responsible for these events have not been identified. In turn, the role of NOB1 endonuclease in the 3′ end maturation of 18S rRNA has been demonstrated [13].

The endonucleolytic steps are followed by exonucleolytic trimming of the resulting intermediates, leading to the formation of mature 5′ and 3′ rRNA ends. This requires the participation of XRN exoribonucleases that process the 5′ ends of 27S-A3 and 26S intermediates, and the exosome complex, containing DIS3/RRP44A and RRP6L2 exoribonucleases, responsible for the 3′ processing of P-A3/P′-A3 species and several precursors of 5.8S rRNA [3, 9, 12, 14, 15]. In yeast, the XRN2 homologue, Rat1, functions redundantly with another 5′-3′ exoribonuclease, Rrp17 [3, 16]. Rrp17 is a relatively short, basic, nucleolar and nucleoplasmic protein that has been identified in a large multi-protein complex involved in the 60S ribosomal subunit assembly and export [16]. A purified Rrp17 protein has been demonstrated to bind and degrade RNA substrates, preferably those with 5′ hydroxyl or monophosphate groups. Lack of Rrp17 results in a strong defect in the formation of mature 5.8S and 25S rRNAs and the accumulation of different rRNA maturation intermediates. Interestingly, cells depleted of Rrp17 accumulate 5.8S rRNA extended at both 5′ and 3′ ends, which suggests that this factor is required for both 5′ and 3′ end processing [16]. 5.8S rRNA exists in two forms, the major short 5.8S_S_ and the long 5.8S_L_, which differ at their 5′ ends [3]. In yeast, the more abundant 5.8S_S_ rRNA is produced by 5′-3′ exoribonucleolytic activities of both Rat1 and Rrp17 following the cleavage at site A3 by RNase MRP, while 5.8S_L_ results from direct endonucleolytic cleavage by an unknown endonuclease [3, 16].

Rrp17 is conserved among eukaryotes and the lack of this enzyme in yeast is complemented by the expression of the human homologue NOL12. Human NOL12 (hNOL12) localizes to the nucleolus and its silencing results in fragmentation of this compartment [17]. Similarly, the knock-down of the *Drosophila melanogaster* Rrp17 homologue *Viriato* strongly affects nucleolar architecture, which is rescued by hNOL12 [18]. hNOL12 has been shown to be required for efficient separation of the large and small subunit pre-rRNAs, but unlike yeast Rrp17, its direct role in rRNA 5′ end trimming has not been demonstrated [19, 20]. In turn, hNOL12 overexpression has been recently linked to the progression of hepatocellular carcinoma [21].

*Arabidopsis thaliana* genome encodes the Rrp17 homologue, AtNOL12, which is weakly conserved except for residues in the catalytic region [19]. Its function has not yet been characterized. Here we show that AtNOL12 is a nucleolar-localized protein, which functions in pre-rRNA processing, similarly to Rrp17 in yeast. Analysis of the *nol12* mutant transcriptome reveals that defects in the pre-rRNA processing pathway are accompanied by deregulation of the expression of a wide range of RP and RBF genes. In addition, the lack of AtNOL12 affects the plant sensitivity to abiotic stress and pathogen attack.

## Results

### Arabidopsis NOL12 contains the conserved catalytic domain and localizes to the nucleolus

**Fig. 1:**
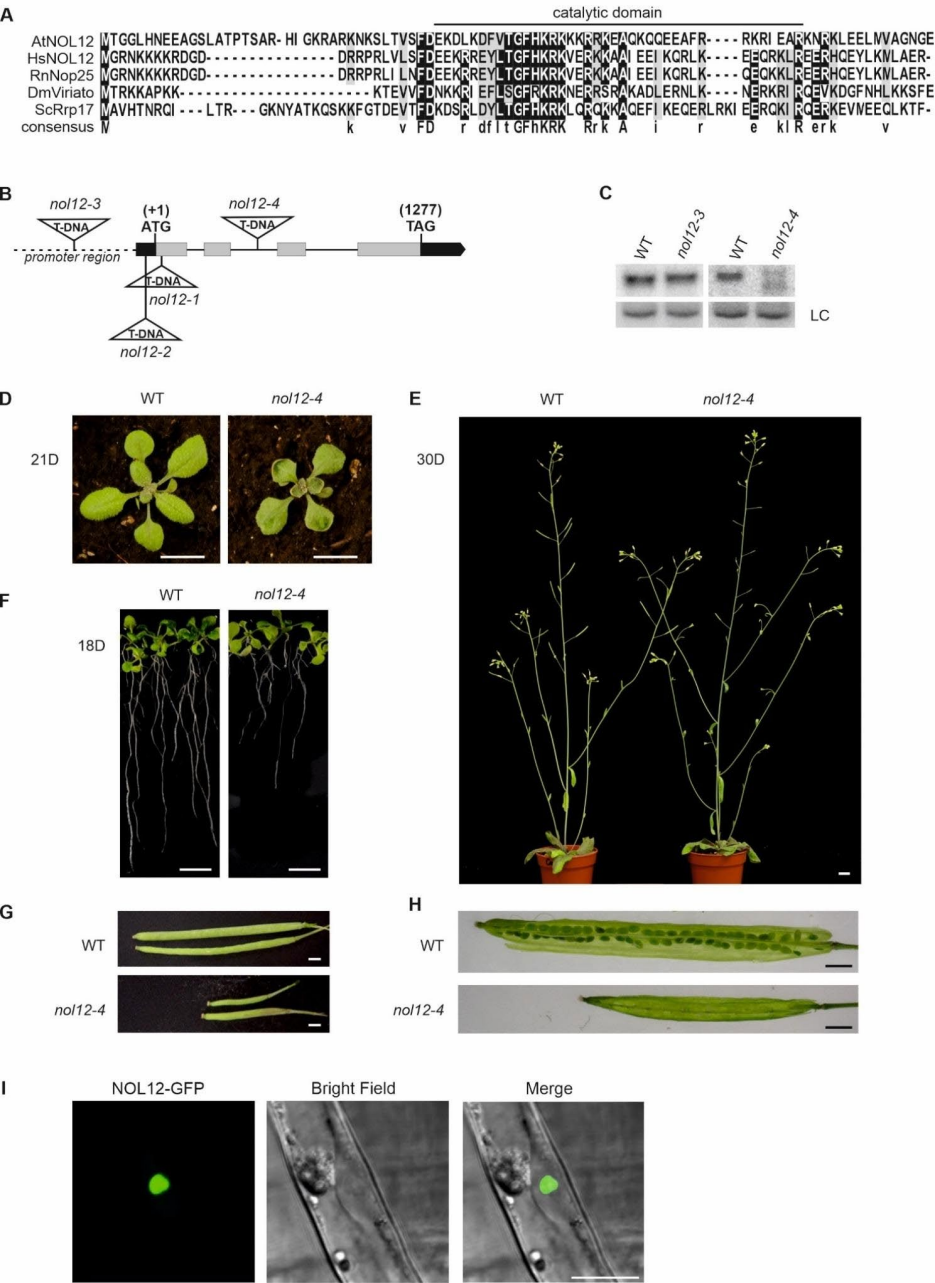
AtNOL12 is a conserved nucleolar protein. **(A)** Fragment of T-Coffee sequence alignment of NOL12 homologues. AtNOL12 (*Arabidopsis thaliana*), HsNOL12 (human), RnNop25 (*Rattus norvegicus*), DmViriato (*Drosophila melanogaster*) and ScRrp17 (yeast *Saccharomyces cerevisiae*). The putative catalytic domain is indicated according to [16, 19]. **(B)** Structure of the AtNOL12 (*At1G11240*) gene. Exons are represented with grey bars and localization of T-DNA insertions are indicated. **(C)** Northern blot analysis of *NOL12* mRNA in wild-type (WT) *nol12-3* and *nol12-4* mutant lines. Hybridization for *EIF4A1* mRNA was used as a loading control (LC). Full-length blots are included in a Supplementary Information file. Analysis was performed in two biological replicates. **(D-H)** The morphological phenotype of *nol12-4* plants. Scale bars represent 1 cm (D-F) or 1 mm (G-H). **(I)** Nucleolar localization of NOL12 fused to GFP observed by confocal microscopy in Arabidopsis root hair cells. Scale bar represents 10 μm

*A. thaliana* Rrp17 homologue AtNOL12 (*At1G11240*) was identified by BLAST analysis [22]. The length and basic amino acid content of AtNOL12 resembles other members of this protein family. Importantly, it contains a catalytic region which is conserved among eukaryotes [19] (Fig. 1A and Supplementary Fig. S1B). Protein sequence-based phylogenetic analysis, including AtNOL12 and 24 homologues from other plant species, both monocots and dicots, also revealed extensive distribution and evolutionary conservation throughout the plant kingdom (Supplementary Fig. S1C). To gain more insight into the function of the AtNOL12 protein in Arabidopsis, we obtained the following *nol12* mutant lines with T-DNA insertions in the *AtNOL12* gene: *nol12-1* (SAIL_309E09), *nol12-2* (SALK_104922) and *nol12-4* (GK-536B01), as well as *nol12-3* (SALK_104924) line with T-DNA insertion in the *AtNOL12* gene promoter (Fig. 1B). We were not able to select homozygous *nol12-1* and *nol12-2* plants, so we excluded these lines from our study. To confirm the knockdown of *AtNOL12* expression we checked the level of AtNOL12 mRNA in *nol12-3* and *nol12-4* mutants by northern blotting (Fig. 1C). Since the *nol12-3* line showed a similar level of *AtNOL12* mRNA as wild-type (WT) plants, the *nol12-4* line was chosen for further analyses. Homozygous *nol12-4* mutants displayed a specific phenotype of upward-curled young leaves (Fig. 1D), characteristic of mutants with 60S maturation defects, such as mutants deficient in LSG1-2 or NSN1 GTPases [23–25].

The curling of the rosette leaves was most evident in the early stages of growth, during leaf development and rosette growth (stages 1–3 according to [26]) and decreased in the later stages (Fig. 1E and Supplementary Fig. S2). *nol12* plants also exhibited shorter roots when grown in vertical plates (Fig. 1F). In addition, the lack of NOL12 caused reduced fertility manifested by smaller siliques (Fig. 1E, G) and a high number of aborted seeds (Fig. 1H). The observed phenotypic features strongly indicate disturbances in developmental processes, from embryo development to leaf shape and root growth regulation.

The visible phenotypes of the *nol12-4* line were rescued by expression of the *AtNOL12* gene fused to GFP (Supplementary Fig. S2, 35S::*NOL12-GFP/nol12-4* line). Since NOL12 homologues in other species localize to nucleoli and perform nucleolar-localized functions, we used the 35S::*NOL12-GFP/nol12-4* line to confirm nucleolar localization of AtNOL12 also in Arabidopsis (Fig. 1I).

The nucleolar localization, the presence of a conserved catalytic domain and the observed phenotype strongly suggest that AtNOL12 may be involved in pre-rRNA processing and plant development, especially in the early stages.

### The *nol12* mutation alters the expression of many ribosome-related genes

**Fig. 2:**
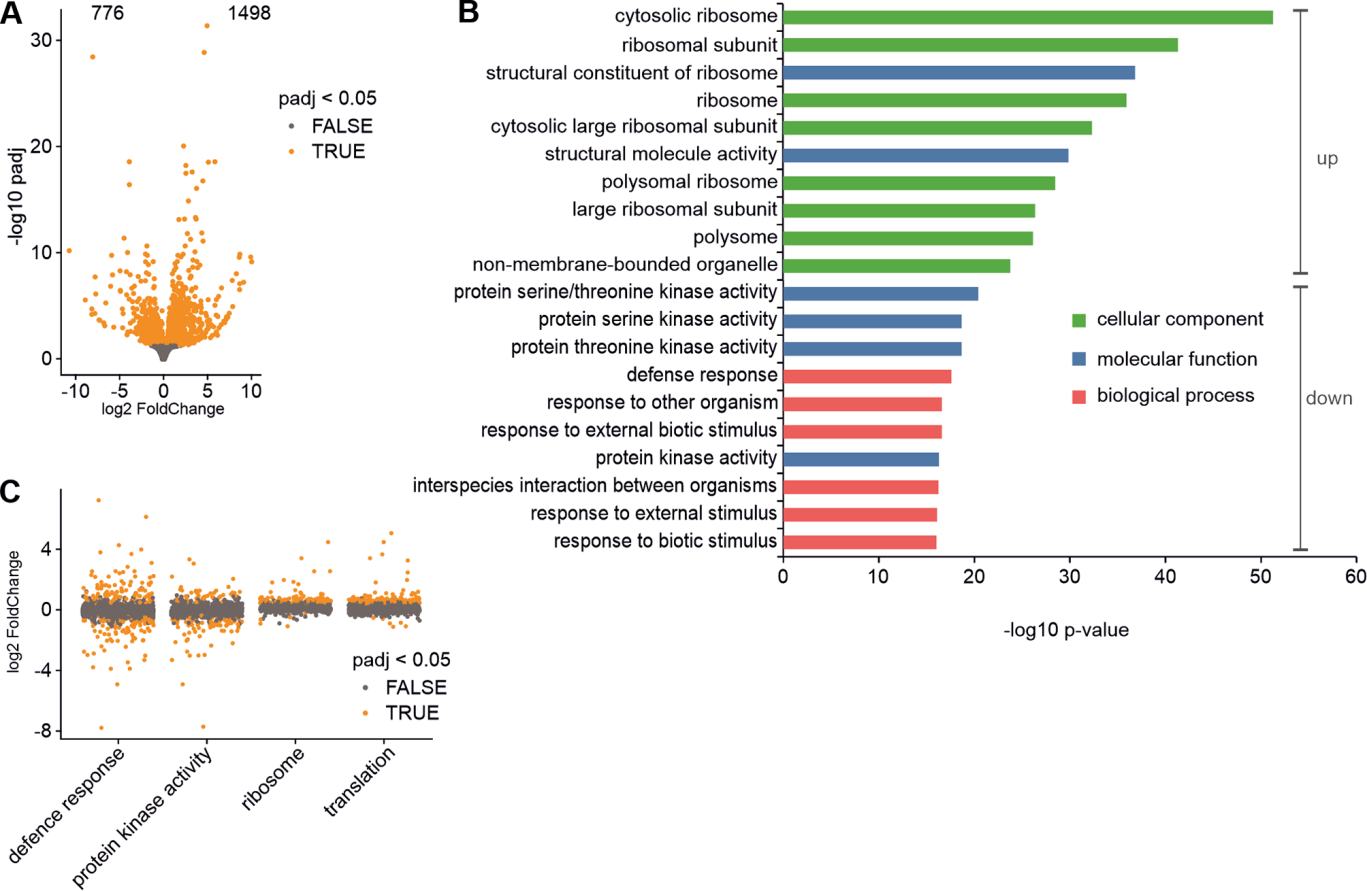
*nol12* mutation affects the expression of genes related to ribosomes and defence response. **(A)** Plot showing 2,274 genes with differential expression in the *nol12-4* mutant. **(B)** GO term analysis of genes with affected expression (padj < 0.05) in the *nol12-4* mutant (shown are only top 10 hits, all results are presented in Supplementary Data Set S1). GO terms enriched among genes with upregulated (up) and downregulated (down) expression, respectively. **(C)** Gene expression changes of genes belonging to selected GO term groups

Phenotypic features observed in *nol12-4* mutant reveal abnormalities in growth and development. In particular, the curled leaf shape is likely due to a disoriented leaf polarity caused by changes in the expression of leaf-polarity-related genes. It is known from research on Arabidopsis that the establishment of abaxial-adaxial leaf polarity can be affected, among others, by ribosome insufficiency or dysfunction [23, 25, 27–29]. Based on the nucleolar localization of AtNOL12 and functions of its homologues in other species we assumed that the phenotypes associated with the lack of this protein may result from the impaired ribosome biogenesis. To identify the set of genes with altered expression in the *nol12-4* mutant we performed transcriptomic analysis (RNA-seq) of total RNA from 14-day-old mutant and WT plants. Differential gene expression analysis revealed 776 downregulated and 1,498 upregulated Araport11 annotated genes, with 462 and 1,032 strongly (absolute log_2_FC > 1) downregulated or upregulated respectively (DESeq2, padj < 0.05; Fig. 2A and Supplementary Data Set S1). Gene Ontology (GO) term enrichment analysis showed that upregulated genes were related to ribosomes and their biogenesis, while downregulated ones were enriched in genes encoding proteins with kinase activity and those involved in defense response against pathogens (Fig. 2B and Supplementary Data Set S1). However, a number of genes associated with these GO terms was also present among upregulated ones. In contrast, translation-related genes with altered expression were almost exclusively upregulated (Fig. 2C).

### AtNOL12 is required for efficient processing of rRNA precursors

**Fig. 3:**
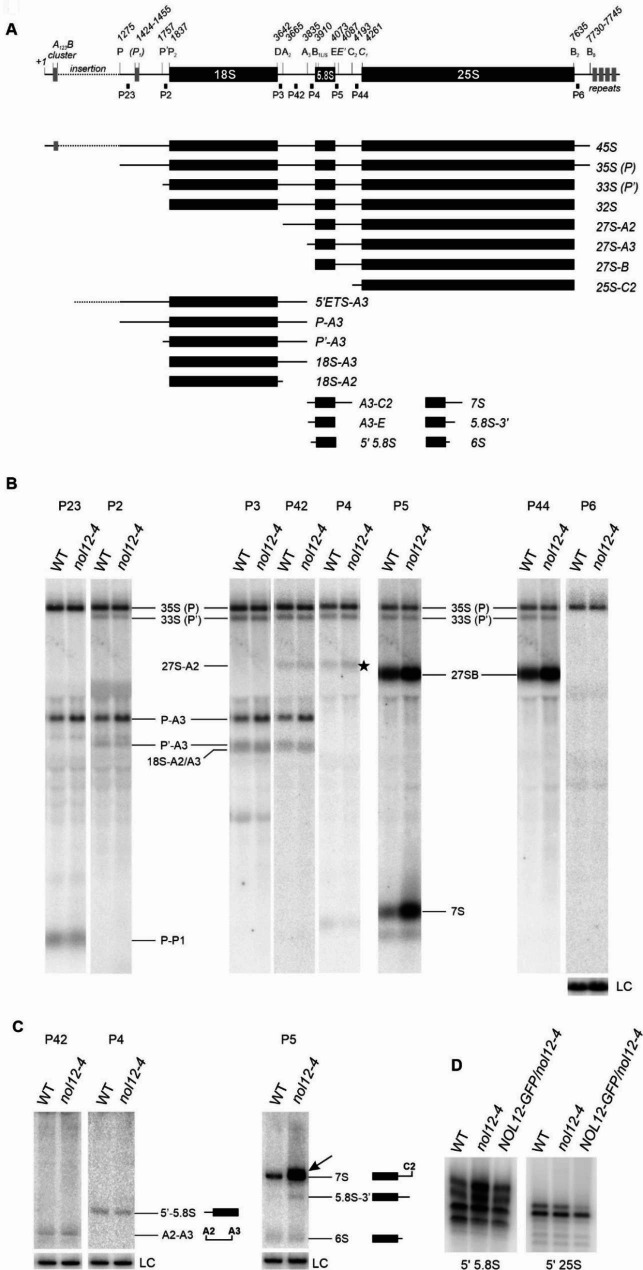
The *nol12-4* mutant accumulates rRNA precursors of the large ribosomal subunit. **(A)** Schematic representation of pre-rRNA structure and processing intermediates. Localization of probes used for northern blot analysis is shown. **(B-C)** Northern blot analysis of high-molecular weight **(B)** and low-molecular-weight **(C)** pre-rRNA precursors in WT and *nol12-4* mutant plants using probes depicted in **(A)**. RNA was separated on 1.1% agarose or 6% acrylamide gels. rRNA precursors and intermediates are indicated on the right. Two large pre-rRNAs, 35S-P and 33S-P′, are not distinguished by northern analysis. Probes for mature 18S rRNA **(B)** and 7SL RNA (C) were used as loading controls (LC). Schematic representation of detected low-molecular-weight pre-rRNAs is shown in **(C)**. Northern blots were performed in at least three biological replicates. **(D)** Primer extension analysis for 5.8S and 25S 5′ ends. Full-length gels and blots are included in a Supplementary Information file

Changes in the expression of genes encoding RP and RBF in the *nol12-4* mutant may result from defects in pre-rRNA maturation due to AtNOL12 dysfunction. Alternatively, impairment of these factors in mutant plants may lead to inefficient rRNA biogenesis. We assumed that, like its homologue Rrp17, Arabidopsis NOL12 acts directly at the 5′ ends of pre-rRNAs, especially that the contribution of 5′-3′ nuclear exoribonucleases XRN2 and XRN3 to these processing steps is limited. Depletion of these proteins led to a modest accumulation of 5′-extended 5.8S and 25S precursors and did not affect the mature rRNAs [9]. This strongly suggests the involvement of a redundant 5′-3′ exonuclease activity. To investigate whether the role of AtNOL12 in rRNA processing is direct, we monitored the pattern and level of rRNA precursors in *nol12-4* plants by northern blotting of total RNA from 14-day-old *nol12-4* and WT seedlings, using a set of probes designed for detection of particular pre-rRNA species (Fig. 3A).

Analysis of high-molecular weight RNAs using probes P5 and P44, located in ITS2, revealed a prominent increase of 27SB pre-rRNA in the *nol12-4* mutant, possibly reflecting delayed or deficient processing in ITS2 in the absence of AtNOL12 (Fig. 3B). Unexpectedly, at this stage of our analysis, we detected the accumulation of 3′-extended, but not 5′-extended, 5.8S rRNA precursors in *nol12-4* plants (Fig. 3B, probes P4 and P5). This observation was in contrast to what was reported for the yeast Rrp17. To inspect the pattern of 5.8S precursor in more detail, we performed northern blots for low-molecular weight RNAs using the same set of probes (Fig. 3C). This allowed us to identify the 7S and 5.8S-3′ species that accumulated in *nol12-4*. We also detected an additional precursor in the mutant which migrated slower than 7S pre-rRNA and was hardly visible in the WT (Fig. 3C, probe P5). This species was not extended at the 3′ end, because it was detected by the P45 probe, located just upstream of the cleavage site C2, but not by P46, located just downstream of this site, which indicates that this species ends directly at C2 (Supplementary Fig. S3C). We assume that the faster-migrating and more abundant precursor present in both plant lines corresponds to the standard 7S pre-rRNA, while the slower-migrating and less abundant species, detected in *nol12-4*, represents 5′-extended forms. Primer extension analysis of 5′ ends of 5.8S/7S rRNAs in *nol12* and WT plants confirmed that 5′-extended species accumulated in the mutant (Fig. 3D). Notably, we did not observe 5′-extended 25S rRNA (Fig. 3D).

This phenotype strongly suggests that Arabidopsis NOL12 participates in 5.8 S rRNA 5′ end maturation together with XRN2/3 exonucleases [9] as is the case in yeast where this processing step is performed by a joint action of Rat1 and Rrp17 [3, 16]. In contrast, while XRN2 is involved in this process in human cells, direct activity of hNOL12 in ITS2 has not been demonstrated [3, 20, 30].

Importantly, an increase of rRNA precursors in the *nol12-4* mutant had no effect on mature rRNAs (Supplementary Fig. S3C and D). Alteration in pre-rRNA level, but not in mature rRNAs, have been reported for mutants in many plant rRNA processing factors (e.g. [9, 12, 31, 32]), presumably due to the coexistence of alternative processing pathways and redundant activities of enzymes involved in plant rRNA maturation.

### The *nol12* mutant shows increased sensitivity to biotic and abiotic stresses

Pre-rRNA processing in plants, as well as in other organisms, is affected by stress conditions and a number of plant mutants in genes involved in ribosome maturation show altered response to various stress conditions, mainly abiotic [1, 33–39]. In addition, the nucleolus is targeted by many plant viruses and other pathogens, linking this subnuclear structure to biotic stress [40]. Moreover, bacterial infection has been recently reported to have an effect on pre-rRNA processing [41].

**Fig. 4:**
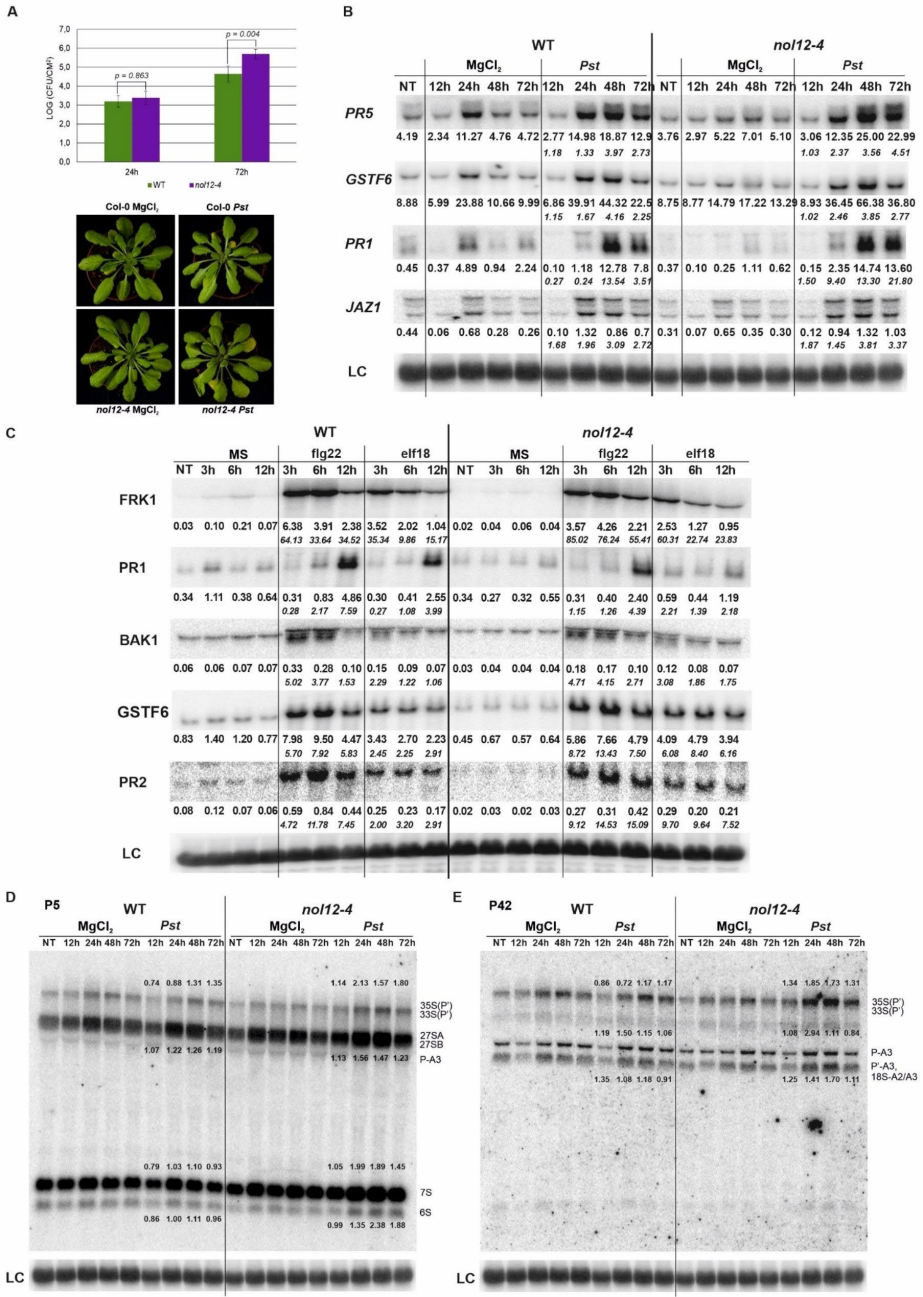
The *nol12-4* mutant is sensitive to *Pseudomonas syrignae* pv. *tomato* DC3000 infection. (**A**) Growth of *Pst* DC3000 after 24 and 72 hpi in WT and the *nol12-4* mutant. Results are the mean of four independent experiments and error bars represent SD; P < 0.01 (Student’s t-test). Lower panel: images of indicated plants infected with *Pst* DC3000 and control plants treated with MgCl_2_ (**B**) Northern blot analysis of factors involved in response to *Pst* DC3000. Samples were collected from non-treated (NT), control (MgCl_2_) and infected (*Pst*) WT and *nol12-4* plants at indicated time points. The ratio of transcript level in treated *nol12-4* versus WT normalized to 18S rRNA loading control (LC) is shown as the main numbers, while the ratio relative to the control conditions is given in italics. (**C**) Northern blot analysis of factors involved in PAMPs response. Samples were collected at indicated time points from non-treated (NT) 14-day-old seedlings, treated with MS (control) or 100 nM of flg22 and elf18. The ratio of transcript level in treated *nol12-4* versus WT normalized to 18S rRNA loading control (LC) is shown as the main numbers, while the ratio relative to the control conditions is given in italics. (**D-E**) Northern blot analysis of pre-rRNA precursors in WT and *nol12-4* lines using probes P5 and P42. Numbers represent the ratio of transcript level in *Pst*-treated WT and the mutant relative to control (MgCl_2_) and normalized to 18S rRNA loading control (LC). Northern analyses in (B-E) were performed in at least three biological replicates. Full-length blots are included in a Supplementary Information file

Our transcriptomic analyses revealed that many genes with downregulated expression in the absence of AtNOL12 were related to defense response and response to biotic stimulus (Supplementary Table S2; see Fig. 2C). Among the most obvious candidates are for example *NIMIN1* (*NIM1-INTERACTING 1*) and *NIMIN2*, *RMG1* (*RESISTANCE METHYLATED GENE 1*), *FRK1* (*FLG22-INDUCED RECEPTOR-LIKE KINASE 1*), *CRK7* (*CYSTEINE-RICH RECEPTOR-LIKE PROTEIN KINASE 7*) and *CRK13*, *ACD6* (*ACCELERATED CELL DEATH 6*), *WRK54*, *PDF1.4* (*PLANT DEFENSIN PROTEIN*) and *PR3* (*PATHOGENESIS-RELATED 3*). To analyze the potential function of AtNOL12 protein in plant innate immunity, we tested 6 weeks-old *nol12-4* and WT plants upon *Pseudomonas syringae* pv. *tomato* DC3000 (*Pst*) infection. *Pst* is a highly virulent bacteria widely used to assess plant-pathogen interactions [42]. Plants have developed a two-step specialized innate immune system. The first, pattern-triggered immunity (PTI), is induced by pathogen-associated molecular patterns (PAMPs), which directly activate the innate immune response in plants. The second step, effector-triggered immunity (ETI), is induced by resistance proteins (*R* proteins), which recognize pathogen effectors [43]. During bacterial infection changes in gene expression result mostly from transcriptional reprogramming [44, 45]. In our experiment, bacterial growth assessed after 72 h post infection (hpi) showed significantly higher pathogen multiplication in the *nol12-4* mutants compared to the WT plants, which indicates that plants lacking AtNOL12 are more sensitive to *Pst* (Fig. 4A). We also investigated changes in mRNA level of key pathogenesis markers (*PR1*, *PR5*, *GSTF6* and *JAZ1*) by northern blot analysis (Fig. 4B and Supplementary Fig. S4A). *PR1*, *PR5* and *GSTF6* are involved in the salicylic acid response pathway, while *JAZ1* is associated with the activation of the jasmonic acid response pathway. These markers were activated after *Pst* infection in both WT and *nol12-4*, however this effect was slightly stronger in the mutant after 48 or 72 hpi (Fig. 4B and Supplementary Fig. S4A).

Next, to investigate the mode of action of PAMP-induced pathways in the *nol12-4* mutant, we treated 14-day-old seedlings with the best known bacterial PAMPs recognized by plants, namely flg22 (peptide fragment of bacterial flagellin) and elf18 (translation elongation factor Tu), and then examined selected mRNAs by northern blot analysis in both WT and mutant plants compared to control conditions. The response pathways of both flg22 and elf18 were similar and triggered the same markers, including *FRK*, *BAK1*, *PR1*, *PR2* and *GSTF6*. However, the response to flg22 was significantly stronger than to elf18 (Fig. 4C, Supplementary Fig. S4B). It can be seen that *FRK1* and *BAK1*, which are recognized by pattern recognition receptors (PRR), were activated within the first 3 h, followed by *GSFT6* and *PR2*, and at the latest *PR1*, which is the basic pathogenic marker. Together these data suggest that the lack of AtNOL12 protein deregulates the response to biotic stress. In addition, the reduced expression of genes associated with pathogen response in the *nol12-4* mutant (see Fig. 2B and Table S2) is consistent with its enhanced susceptibility to the pathogen.

To check the effect of pathogen attack on pre-rRNA processing we performed northern blot analysis for *nol12-4* and WT plants infected by *Pst*. Using P5 and P42 probes, which show the most evident differences between the mutant and WT, we detected an increase in 35S-P/33S-P′, 27S, 7S and P-A3 intermediates that was more pronounced in *nol12-4* (Fig. 4D and E). This indicates that, on the one hand, pathogen attack affects pre-rRNA processing, as already demonstrated by [41], and on the other hand, that AtNOL12 plays a role in regulating the rRNA maturation pathway under biotic stress. In this experiment, we observed a weaker accumulation of 27S and 7S in the mutant under control conditions compared to our previous experiments. This is probably due to the use of much older, 6-week-old plants for *Pst* infection, as opposed to 14-day-old seedlings used in other experiments.

Since increased responsiveness to biotic stimuli usually coincides with higher sensitivity to abiotic stress, we subjected 14-day-old seedlings of WT, *nol12-4* mutant and 35S::*NOL12-GFP/nol12-4* transgenic line to salinity (100 mM NaCl), sugar (3% glucose or media without sugar) and heat stress, which are the major abiotic factors causing severe plant damage, and heat stress has already been shown to impact pre-rRNA processing and ribosome maturation [38, 39]. In our case only heat stress showed significant effect on rRNA maturation pathway.

**Fig. 5:**
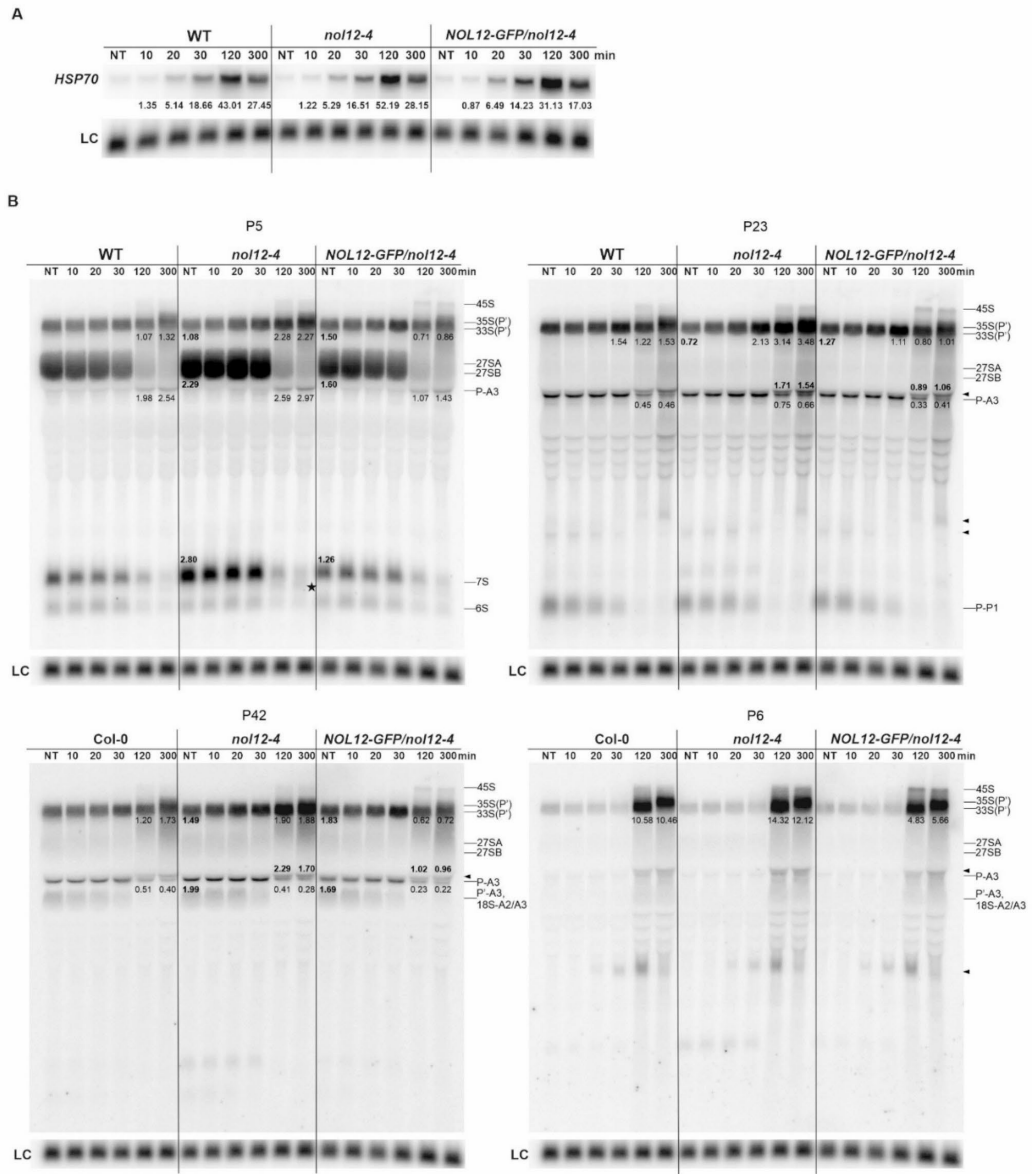
AtNOL12 affects pre-rRNA processing under heat stress. Northern blot analysis of mRNA **(A)** and rRNA precursors and intermediates **(B)** in WT, *nol12-4* and 35S::NOL12-GFP/*nol12* lines. Samples were collected from 14-day-old seedlings and transferred to 37°C for the times indicated. RNA was separated on 1.1% agarose gels and hybridized with *HSP70* **(A)** and probes P5, P23, P42, and P6 **(B)**. rRNA precursors and intermediates are described on the right. Black arrowheads indicate the heat stress-dependent species, including the P-C2 intermediate, migrating just above the P-A3 [46], asterisk indicates a *nol12*-specific intermediate that probably corresponds to 5.S-3′ species. The values provided above or below RNA intermediates show changes in their accumulation in the mutants compared to WT; numbers in bold represent relative values in the mutants compared to the WT at the corresponding treatment time, the remaining numbers denote relative values compared to the untreated plants (NT) for the same line. U2 snRNA was used as a loading control (LC). The analyses were performed in three biological replicates. Full-length blots are included in a Supplementary Information file

To obtain a comprehensive picture of pre-rRNA processing under heat stress, we performed northern analysis for WT, *nol12-4* and 35S::NOL12-GFP/*nol12-4* seedlings plants transferred to 37°C for 10, 20 min, 30 min, 2 and 5 h (Fig. 5), as well as to 42°C for 2 h and 5 h (Supplementary Fig. S5). Induction of response to heat stress was confirmed by checking the levels of known stress marker *HSP70* by northern blot (Fig. 5A and Supplementary Fig. S5A). Moderate effect on accumulation of *HSP70* mRNA in *nol12-4* indicates that the mutant is more sensitive to heat than WT plants.

As previously reported [39], a short exposure to elevated temperature did not induce any changes in the rRNA precursor profile, which were clearly visible after 2 and 5 h. Under these conditions we observed an accumulation of the largest pre-rRNAs, namely 45S and 35S-P/33S-P′, and a concomitant decrease in downstream pre-rRNA processing intermediates (27S, 7S) in all tested plant lines, confirming that rRNA maturation was inhibited at elevated temperatures (Fig. 5B) as shown before [38, 39]. The accumulation of 35S-P/33S-P′ species, but not 45S, was visibly stronger in the *nol12-4* mutant compared to the WT and 35S::NOL12-GFP/*nol12-4* lines.

Additional pre-rRNA intermediates, more prominent in the *nol12-4* mutant, were detected in the heat-treated seedlings, P-C2 fragment with probes P23 and P42 (Fig. 5B, marked with an arrowhead), and most likely the 5.8S-3′ precursor with probe P5 (Fig. 5B, marked with an asterisk). The accumulation of the P-C2 intermediate is a hallmark of a stress-dependent pre-rRNA processing pathway called “ITS2-first”, where the initial cleavage of the primary precursor occurs at site C2 in ITS2 [1, 38, 39, 46]. Similar results were obtained at 42°C, but a long exposure to such a high temperature deteriorates the condition of plants, which in turn affects the quality of RNA (Supplementary Fig. S5).

These data confirmed that AtNOL12 is involved in ITS2 processing and 5.8 S rRNA 5′ end maturation, and revealed that this protein contributes to the regulation of these events under both biotic and abiotic stress conditions.

### *nol12* mutants exhibit improved resistance to cycloheximide

**Fig. 6:**
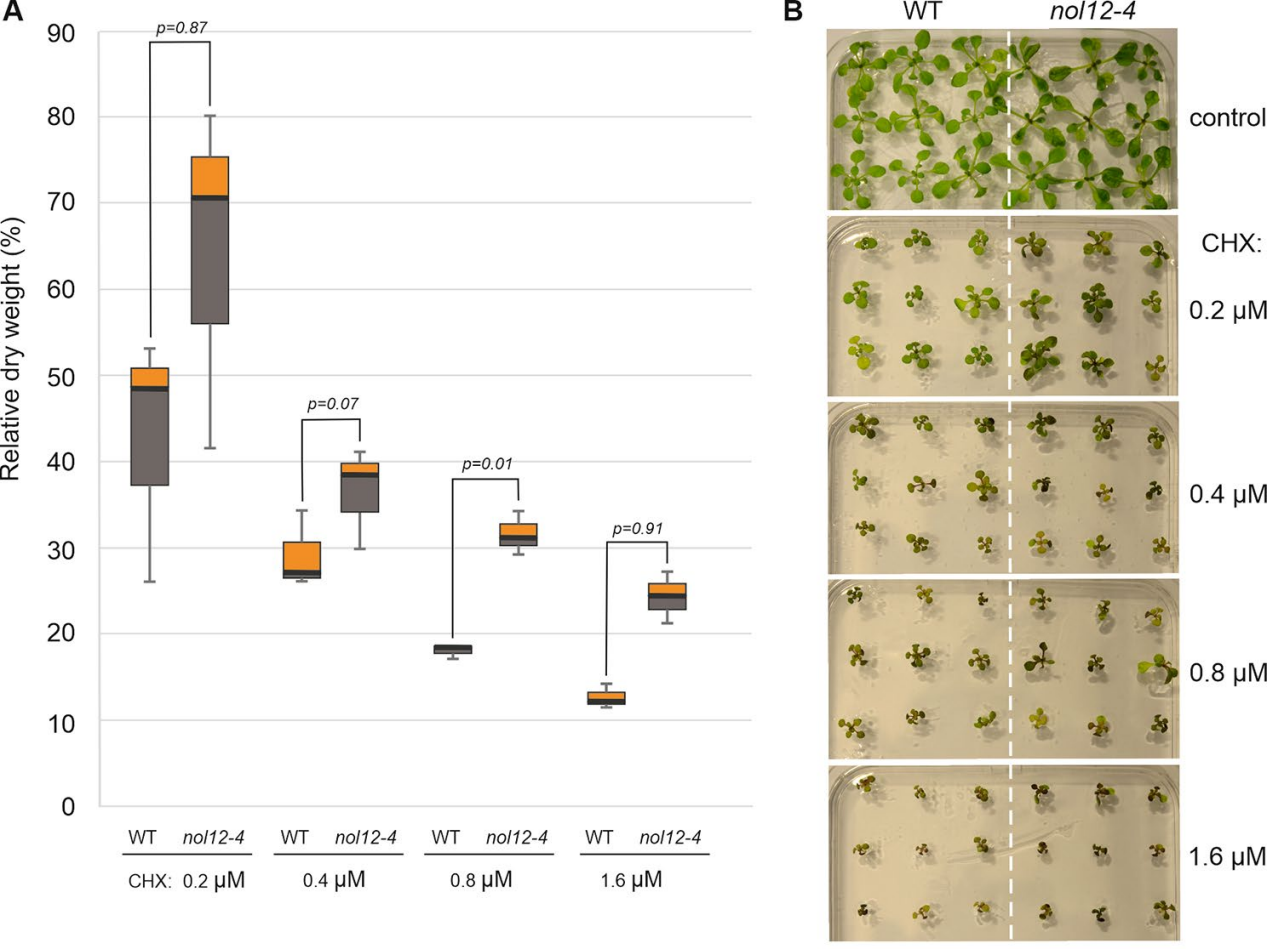
The *nol12-4* mutant presents reduced sensitivity to cycloheximide. **(D)** Box plot for relative dry weight of WT and *nol12-4* plants, grown on plates with indicated CHX concentration, normalized to respective plant lines grown on CHX-free medium. The experiment was performed in three biological replicates, for 27 plants in each replicate. Dark horizontal lines represent the median, with the box representing the 25th and 75th percentiles and the whiskers the 1.5 IQR limits. *p-values* by ANOVA test among WT and *nol12-4* are depicted. **(E)** WT and *nol12-4* plants grown on plates containing increasing concentrations of CHX

A noticeable number of Arabidopsis ribosome biogenesis mutants exhibit altered sensitivity to several antibiotics, which is likely caused by the production of ribosomes with abnormal structure and function due to aberrant pre-rRNA processing and ribosome assembly [29, 32, 35, 47–51]. To test whether ribosome activity is impaired in *nol12-4* mutant, we assessed the mutants’ sensitivity to a set of antibiotics. We have chosen four antibiotics known to target particular ribosomal locations in prokaryotes and have been shown to differentially inhibit the growth of mutants in ribosome biogenesis factors relative to WT plants, namely the aminoglycosides streptomycin, spectinomycin and erythromycin, as well as non-aminoglycoside chloramphenicol. To analyze the rate of growth of *nol12-4* and WT plants on antibiotic-containing media we measured the root length of 14-day old seedlings. Compared to the WT, the growth of the mutant roots was less inhibited on media containing spectinomycin or streptomycin, however, the results for erythromycin and chloramphenicol showed no statistically significant differences (Supplementary Fig. S6A-B). We also tested the sensitivity of *nol12-4* to the eukaryotic protein synthesis inhibitor cycloheximide (CHX), which binds eukaryotic ribosomal complexes at the E-site of the 60S subunit [52]. *De novo* protein synthesis is required for radicle protrusion in Arabidopsis and CHX strongly inhibits this process [53], therefore seedlings grown on CHX-containing media have strongly shortened roots (Supplementary Fig. S6C). To reliably estimate the rate of seedling growth in this experiment, we measured the dry weight of seedlings grown on media supplemented with increasing concentration of CHX, ranging from 0.2 µM to 1.6 µM. Compared to WT, growth of the *nol12-4* mutant was moderately less inhibited on media containing CHX and this effect was progressively stronger with increasing concentration of cycloheximide (Fig. 6).

The limited resistance of *nol12-4* plants to certain antibiotics and CHX may result from the presence of a population of structurally altered ribosomes, that are less susceptible to binding these chemicals. There is increasing evidence that plants have specialized functional subpopulations of ribosomes that are organ- or development-specific, and this ribosome heterogeneity plays a regulatory role in translatome reprogramming in response to environmental stimuli or stress conditions (reviewed in [54, 55]). It is possible that the moderately altered antibiotic resistance of *nol12-4* reflects an imbalance in the content of specialized ribosome fractions caused by changes in RP levels, even though the mature ribosomal RNAs are not affected and the ribosomes are functional, as evidenced by the lack of overall effect of these antibiotics on mutant plants. In addition, the increased resistance of *nol12-4* seedlings to CHX suggests that the mutant has altered translational activity. Taken together, our data show that AtNOL12-dependent aberrant rRNA processing may have a modest impact on normal ribosome function.

## Discussion

### AtNOL12 is a conserved nucleolar protein involved in pre-rRNA maturation

NOL12 homologues have been identified in various organisms, and plant AtNOL12 contains the evolutionarily conserved catalytic domain, suggesting conservation of protein function ([16] and this work). The best described homologue, the yeast Rrp17, has the 5′-3′ exoribonucleolytic activity, necessary for digestion of the 5′ ends of 5.8S and 25S rRNAs and is required for processing of the 27S-A3 pre-rRNA [16]. In turn, human hNOL12 participates in processing within ITS1 and its downregulation leads to a decrease in the resulting intermediates [19, 20]. Our work revealed that the activity of AtNOL12 overlaps to a greater extent with the yeast counterpart. Accumulation of 27S and 5′-extended 5.8S rRNA precursors observed in the *nol12-4* mutant clearly suggests the requirement of AtNOL12 for efficient excision of ITS2. On the other hand, the possible role of hNOL12 in ITS2 processing can not be excluded, as enrichment of this protein by eCLIP analysis was detected in both ITS1 and ITS2 regions [56]. There are numerous indications that also in yeast ITS2 processing is dependent on the effective removal of ITS1 [57–60]. In Arabidopsis, accumulation of 27S pre-rRNA may be due to insufficient 27S 5′ trimming in the absence of the possible 5′-3′ exoribonucleolytic activity of AtNOL12. This could lead not only to the increased level of 5′-extended form of 5.8S rRNA in *nol12-4* plants, but also to defective processing within ITS2, resulting in the accumulation of 27S pre-rRNA. The endonucleases involved in cleavages in ITS1 in Arabidopsis are unknown, but the downstream 27S-A2 and A3-C2 products accumulate in AtXRN2 and AtXRN3 exoribonuclease mutants, supporting the involvement of these two enzymes, and probably also AtNOL12, in 27S 5′ processing [9]. Rrp17 has been shown to act in parallel with Rat1/Xrn2 in analogous events in yeast [16].

Surprisingly, in contrast to yeast Rrp17, we did not detect 5′-extended 25S rRNA in the *nol12-4* mutant. We assume that in plants maturation of 25S rRNA 5′ ends is performed by the redundant activity of AtXRN2 and AtXRN3. This is supported by accumulation of the 25S-C2 species in the double *xrn2 xrn3* mutant, but not in *nol12-4* ([61] and this work).

### Possible effect of AtNOL12 absence on ribosome structure and/or function

Defects in pre-rRNA processing in the *nol12-4* mutant do not lead to changes in the levels of mature rRNAs, which has been also observed for other plant rRNA biogenesis mutants [9, 12, 31, 32]). This is most likely due to the redundancy of ribosome maturation pathways, including the stress-dependent bypass that is considered to be essential to maintain ribosome quantity and function [46]. However, altered rRNA processing might affect ribosome assembly, which may entail qualitative changes in the protein composition and structure of mature ribosomes. This in turn can potentially be reflected in widespread upregulation of RBF and RP gene expression, as is the case in *nol12-4*, to compensate for disturbed ribosome homeostasis. Altered composition or structure of ribosomes caused by lack of AtNOL12 is further supported by improved resistance of the *nol12-4* mutant to cycloheximide and ribosome-targeting antibiotics. A limited effect of these chemicals on the mutant growth may reflect the heterogeneity of ribosomes, which allows for changing the composition of ribosomes in response to adverse conditions to sustain translational activity [54, 55, 62]. Ribosome heterogeneity leading to specialized ribosomes is based on diverse ribosome subpopulations containing RP paralogs, additional protein components, RPs with changed stoichiometry or posttranslational modifications, and finally distinct rRNA variants. For example, perturbations in the expression of RPs in yeast and human cells has been reported to alter transcription and translation of specific gene subsets without affecting overall protein synthesis [63, 64]. It is therefore possible that defects in rRNA processing, especially under stress, and/or dysregulated expression of RP genes in the *nol12-4* mutant may affect the balance of ribosome subpopulations and, consequently, the transcriptomic profile.

### Contribution of AtNOL12 to plant stress response and development

Among genes differentially expressed in the absence of AtNOL12, in addition to those related to ribosomes and their biogenesis, we also detected genes involved in stress/defence responses and cell cycle regulation. This is consistent with altered sensitivity of *nol12-4* plants to biotic and abiotic stresses. Our results suggest that AtNOL12, like SAHY1 and AtRH7, represents another factor linking nucleolar functions and stress signalling pathways [34, 35, 37]. Although only a few cases of connections between nucleolar factors and plant immunity have been described so far [41], nucleolus is known to play an important role in detecting stress such as salinity, drought and temperature [40, 65]. In human cells hNOL12 has been reported to be required for genome integrity, DNA damage repair, cell proliferation and apoptotic response [19]. We cannot exclude that AtNOL12 also regulates similar processes, but we did not find genes related to these categories among DEGs in the *nol12-4* mutant (Supplementary Dataset S1), suggesting that the plant homologue is probably not involved in these activities. These differences between the human and plant counterparts are most likely due to their somehow distinct localization profile. While AtNOL12 is predominantly nucleolar, hNOL12 is also present in numerous foci, namely in paraspeckles in the nucleoplasm and in the GW/P-bodies in the cytoplasm [19].

In Arabidopsis, the biogenesis and function of ribosomes is also related to the regulation of development. This is supported by observations that not only pre-rRNA processing, but also many developmental processes are affected by impaired function of RBFs, including embryo development, cell cycle progression and leaf shape regulation [6, 12–14, 24, 25, 31–33, 66–70]. Our RNA-seq data show that AtNOL12 is not only involved in ribosome biogenesis, but also regulates the expression of genes related to cell cycle and cell division (Supplementary Data Set S1). This suggests that ineffective ribosome maturation may induce nucleolar stress, which leads to inhibition of cell cycle progression [71]. Similarly, knockdown of *Drosophila* NOL12 homologue *Viriato* has been reported to inhibit cell growth [18]. Alternatively, disturbances in the cell cycle and cell division in *nol12-4* plants may be partly due to defects in the synthesis of the cell wall, since genes involved in this process were also significantly enriched among DEGs in the mutant (Supplementary Data Set S1). It is known that cell wall integrity influences many biological processes in organisms, including cell cycle and development in plants [72, 73]. Rrp17 in yeast is directly involved in nucleolar export of the 60S ribosome subunit [16], and defects in this process in *Arabidopsis* are linked to abnormal secondary wall formation [74]. These clues point to a potential role for AtNOL12 in pre-60S subunit export from the nucleolus, which may underlie the developmental defects observed in the absence of this protein.

## Conclusions

To conclude, our data confirmed that AtNOL12 is involved in the maturation of rRNA precursors destined for the large ribosomal subunit, in particular in the processing of ITS2 and 5.8 S rRNA 5′ end maturation, and revealed that this protein contributes to the regulation of these events under both biotic and abiotic stress conditions. These findings complement previous reports on the effects of stress on ribosome biogenesis and add to our understanding of the pre-rRNA processing pathway in plants.

## Materials & methods

### Plant material and growth conditions

The *Arabidopsis thaliana* wild-type (WT), mutant and transgenic plants used in the study were all in the Columbia-0 (Col-0) genetic background. We used the following mutant lines with T-DNA insertion in the *AtNOL12* (*At1G11240*) gene: *nol12-1* (SAIL_309E03), *nol12-2* (SALK_104922), *nol12-3* (SALK_104924), *nol12-4* (GABI-Kat GK-536B01), and transgenic *nol12-4* line expressing *AtNOL12* gene fused to GFP (35S::NOL12-GFP/*nol12-4*). Unless stated otherwise plants were grown in Percival growth chambers at 22/19°C on a 16-h-light and 8-h-dark (long-day) photoperiod. Seeds were germinated in soil or were surface-sterilized with 30% bleach/0.02% Triton-X100 solution and germinated on half-strength MS [75] basal salt medium supplemented with 1% (w/v) sucrose and 0.3% phytagel (Sigma-Aldrich). Construction of 35S::NOL12-GFP/*nol12-4* transgenic line: *AtNOL12* coding sequence was PCR-amplified and cloned into pDONR201 vector (Invitrogen), then transferred using LR Clonase (Thermo Scientific) to pGWB605 vector containing C-terminal GFP tag, under the control of 35S promoter. Resulting plasmid was used for *Agrobacterium*-mediated transformation of WT plants by floral-dip method [76].

### Sequence analysis

Plant NOL12 homologue was identified with the blastp algorithm [22]. Sequences were aligned using the T-Coffee web server [77] and rendered with Boxshade. For phylogenetic analysis protein sequences of AtNOL12 homologs were obtained from UniProt (uniport.org), aligned using MAFFT [78] and analyzed using the Neighbor-Joining method. Phylogenetic tree was visualized using Phylo.io application [79].

### Confocal microscopy

Subcellular localization of NOL12-GFP in *A. thaliana* was evaluated using a Nikon C1 confocal system built on TE2000E and equipped with a 60× Plan-Apochromat oil immersion objective (Nikon Instruments B.V. Europe, Amsterdam, The Netherlands). GFP fusion proteins were excited by a Sapphire 488 nm laser (Coherent, Santa Clara, CA, USA) and observed using the 515/530 nm emission filter. Confocal images were analyzed using free viewer EZ-C1 and ImageJ software.

### RNA methods

Total RNA extraction: total RNA was isolated from 14-day-old seedlings or 21-day-old mature plants using TRI Reagent (Sigma-Aldrich) according to the manufacturer’s instructions.

RNA-seq: WT *A. thaliana* Col-0 and *nol12* mutant seeds were sown on MS plates and stratified for 2 days at 4°C. Plants were grown in long-day conditions and harvested at age of 14 days. Three biological replicates were prepared for each genotype and total RNA was isolated using the Trizol method. Libraries were prepared using Illumina TruSeq Stranded Total RNA with Ribo-Zero Plant rRNA Removal (Plant Leaf) protocol including barcoding and were then paired-end sequenced on HiSeq4000 by DNA Research Centre (Poznan, Poland). The raw sequence data have been deposited in the Gene Expression Omnibus (GEO) database under accession code GSE232067. Obtained fastq files were quality checked using *fastqc* (v0.10.1 -http://www.bioinformatics.babraham.ac.uk/projects/fastqc/) with all the replicates showing high-quality RNA-seq data. The reads for each sample were aligned to the TAIR10 *A. thaliana* genome from ensembl (release v29; [80]) using HISAT2 (v2.0.4; [81]) with the following command-line parameters: *--fr --rna-strandness RF --known-splicesite-infile.* Mapped reads were sorted using samtools sort (v1.1; [82]). Reads were counted with *htseq-count* (v0.6.0; [83]) and the following command-line parameters: *-a 0 -s reverse* using Araport11 gene annotation (release 201,604; https://www.araport.org/). Differential expression was performed using *DESeq2* (v1.8.2; [84]) *R* (v3.2.2) package with parameter *alpha = 0.05*. Genes with FDR < 0.05 were considered significantly changed and those with FDR < 0.05 and absolute log_2_FC > 1 were strongly affected. GO enrichment analysis was performed using gprofiler (https://biit.cs.ut.ee/gprofiler/page/r) with FDR < 0.01.

Northern blotting: low-molecular weight RNAs were separated on 6% acrylamide/7 M urea gels and electrophoretically transferred to a Hybond-XL membrane (GE Healthcare). High-molecular-weight RNAs were analysed on 1.1% agarose/6% formaldehyde gels and transferred to a Hybond-XL membrane by capillary elution. γ-³²P 5′-end-labelled oligonucleotides (Supplementary Table S1) were used as probes against precursor and mature rRNAs and U2 snoRNA-specific probe for a loading control. The probes were end-labeled using T4 Polynucleotide Kinase (Thermo Fisher Scientific) with [γ-³²P] (SRP-201, Hartmann Analytic) as per manufacturer’s instructions. For detection of mRNAs random primed probes were used. Random primed probes were amplified on a cDNA template using respective primers and DECAprime™ II labeling kit (Thermo Fisher Scientific) and [α-³²P] dATP (SRP-203, Hartmann Analytic). Quantification of northern blots was performed using Typhoon FLA 9000 Gel Imaging Scanner (GE Healthcare) and ImageQuant (GE Healthcare) or ImageJ (U. S. National Institutes of Health, https://imagej.nih.gov/ij/) software.

Primer extension: for the detection of 5.8S rRNA 5′ ends primer extension analysis was done using 10 µg of total RNA and specific 5′-end-labeled oligonucleotides. The oligonucleotides were labeled using T4 Polynucleotide Kinase (Thermo Fisher Scientific) with [γ-³²P] (SRP-201, Hartmann Analytic) as per manufacturer’s instructions, and then ethanol precipitated. RNA was denatured at 80°C and then hybridized to 1 pmol of labeled primer by slowly cooling the reaction mixture to 50°C. Reverse transcription was performed using SuperScript IV Reverse Transcriptase (Invitrogen) for 15 min at 50°C in 20-µl reactions containing 1 mM dNTPs, 10 units of RiboLock (Thermo Fisher Scientific) and 1x SuperScript IV buffer (Invitrogen), and stopped by 10 min. incubation at 80°C. Products of the reaction were denatured at 95°C for 5 min and separated on 6% acrylamide/7 M urea sequencing gels.

### Bacterial Infection assays, stress and antibiotic resistance tests

Bacterial infection assays were performed with virulent *Pseudomonas syringae* pv. *tomato* strain DC3000 (*Pst*). Bacteria for inoculation were grown overnight in LB medium with rifampicin at 28°C, centrifuged at 3500 g for 7 min, washed, and resuspended in 10 mM MgCl_2_ with density adjusted to 10^6^ cfu ml^− 1^ (OD600nm = 0.003). 6-week-old plants were inoculated by spraying with the *Pst* suspension in 0.05% Silwet L-77 or with 10 mM MgCl_2_/0.05% Silwett L-77 (control plants) and then covered with plastic lids overnight. The material was harvested from at least 10 plants for each time point, frozen in liquid nitrogen, and used for RNA extraction. Bacterial growth was quantified by assessing the number of dividing bacterial cells 24 and 72 h after infection (hpi). Leaves were harvested and surface-sterilized (15 s in 70% ethanol, followed by 15 s in sterile water). Samples (four-leaf discs) were taken using a cork-borer (4 mm) from 2 leaves, ground in sterile Mili-Q water, diluted and plated on a selection medium. Plates were incubated at 28°C and colonies were counted after 48 h.

PAMP assays: sterilized seeds were grown on MS plates as described above. Five days after stratification seedlings were transferred to a 24-well plate with liquid MS (two seedlings per well, 1 ml of medium). MS was exchanged for a fresh medium after 8 days (500 µl per well) and the next day 500 µl of flg22 (Alpha Diagnostic International Inc.) or elf18 (synthesized by GL Biochem Ltd, Shanghai, China) solution was added to each well to a final concentration of 100 nM. Seedlings were harvested at the indicated time points and frozen in liquid nitrogen. Samples collected from each experiment were subjected to RNA isolation and northern blot analysis.

Stress tests: In the case of high-temperature stress, seeds were surface-sterilized and grown on MS medium supplemented with 1% (w/v) sucrose and 0.3% phytagel under long-day conditions, then after 2 weeks plates were transferred to a 37 or 42°C growth chamber. Samples were collected at the indicated time, blotted dry, and frozen in liquid nitrogen. For salt treatment, seeds were grown on an MS medium containing 100 mM NaCl, 1% (w/v) sucrose, and 0.3% phytagel (Sigma-Aldrich). After 14 days, the seedlings were harvested and frozen in liquid nitrogen. RNA was then isolated and analysed by northern blotting.

Antibiotic tests: the seeds were initially germinated on MS medium supplemented with 1% (w/v) sucrose and 0.3% phytagel, without antibiotics, under long-day conditions, for 5 days, and then transferred to the antibiotic-containing plates. Root length and dry weight measurements were performed after 14 days of growth in antibiotic-containing media. Dry weight measurements of plants grown on CHX media were performed after drying seedlings in 70°C overnight. The experiment was conducted in three biological replicates, 27 plants were analyzed for each replicate.

### Electronic supplementary material

Below is the link to the electronic supplementary material.

Supplementary Material 1

Supplementary Material 2

## Data Availability

The data that support the findings of this study are openly available; RNA-seq data are deposited in the Gene Expression Omnibus database under accession code GSE232067. The plasmids and plant lines generated during the study are available from the corresponding author on reasonable request.
