# Supplementary material for: The nucleolar protein NOL12 is required for processing of large ribosomal subunit rRNA precursors in Arabidopsis
Source: BMC Plant Biol. 2023 Nov 3;23:538. doi: 10.1186/s12870-023-04561-9 (PMC10623804; doi:10.1186/s12870-023-04561-9)

SUPPLEMENTARY FIGURES

Figure S1

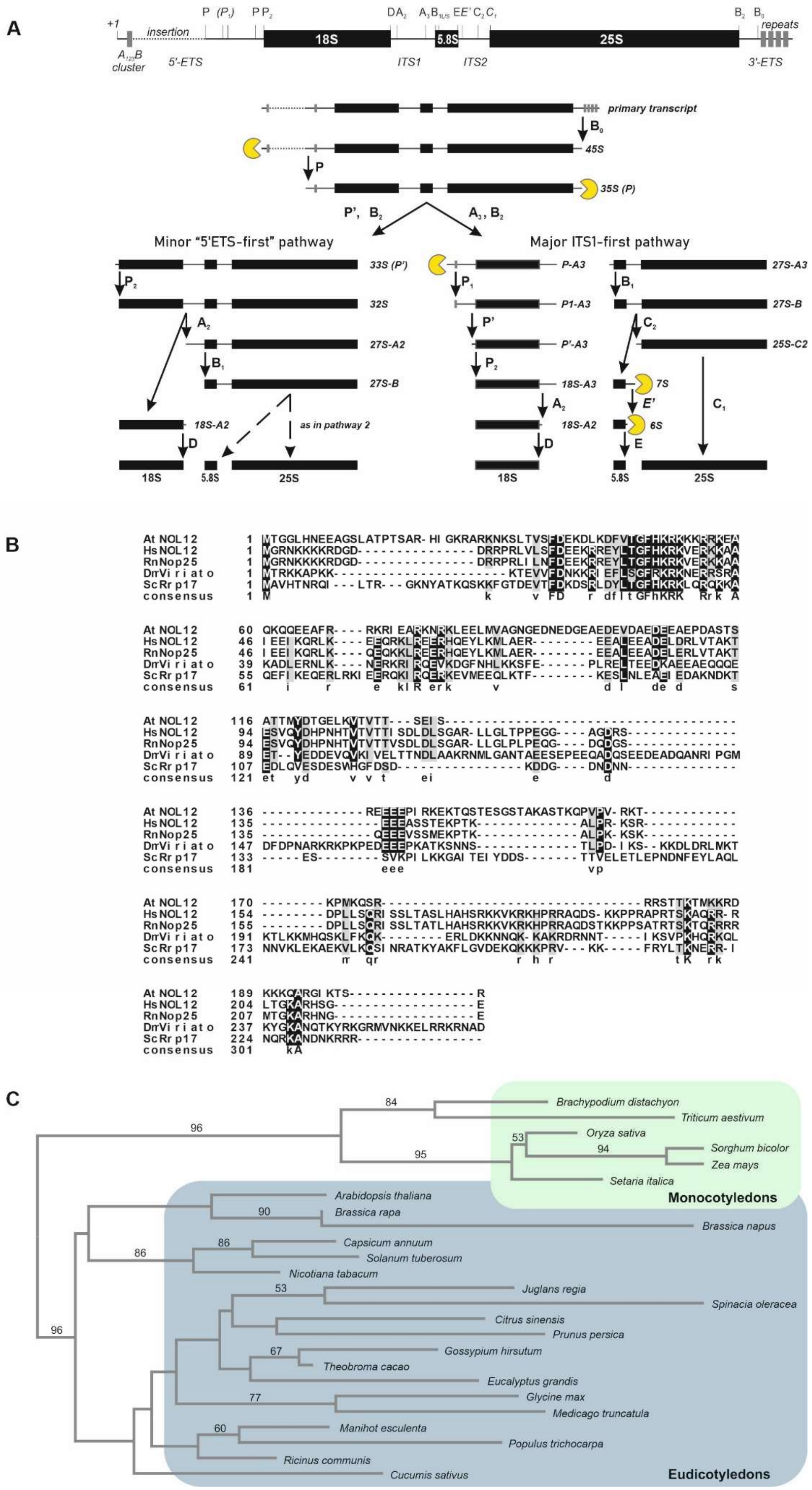

Supplementary Figure S1. Pre-rRNA processing pathway. (A) Schematic representation of pre-rRNA structure and processing in Arabidopsis. **(B)** T-Coffee sequence alignment of NOL12 homologues. AtNOL12 (*Arabidopsis thaliana*), HsNOL12 (human), RnNop2512 (*Rattus norvegicus*), DmViriato (*Drosophila melanogaster*) and ScRrp17 (yeast *Saccharomyces cerevisiae*). (C) Phylogenetic analysis of AtNOL12 homologues in other plant species, including Monocotyledons (highlighted in green) and Eudicotyledons (in blue). The tree was derived by neighbor-joining distance analysis. Bootstrap values over 50% are indicated above the nodes.

Figure S2

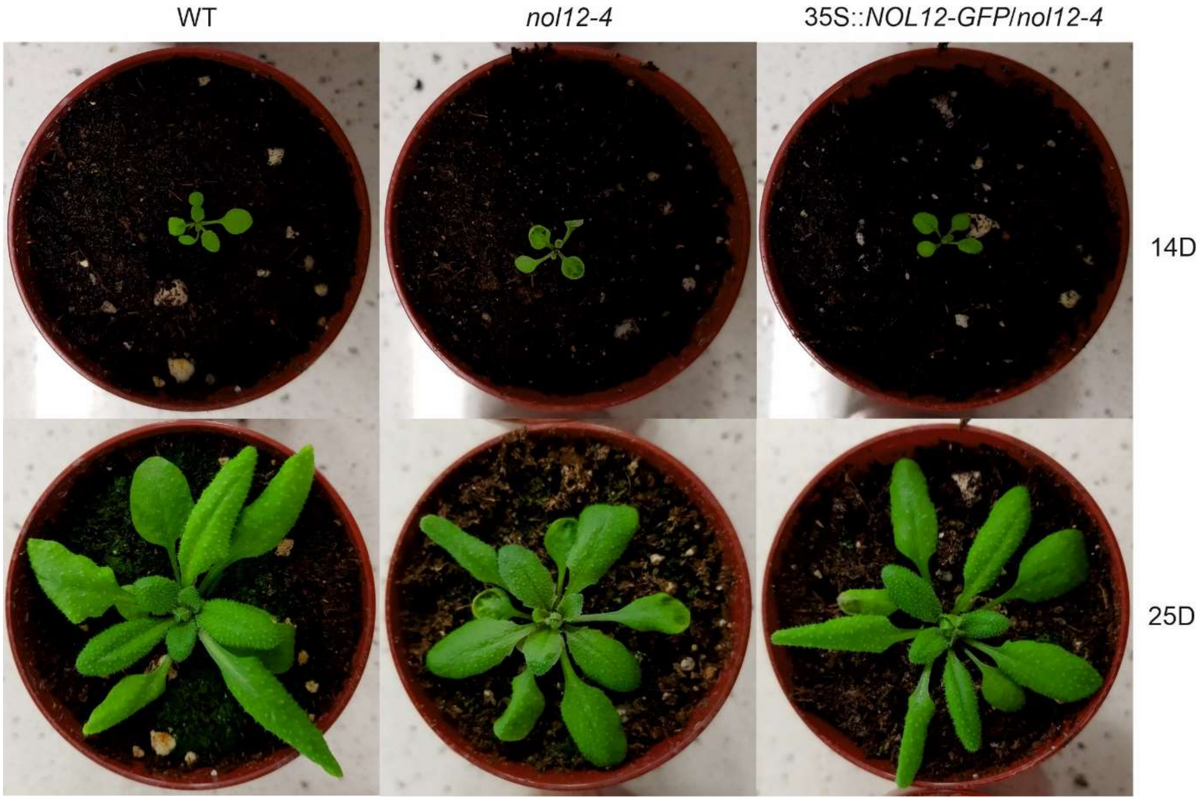

Supplementary Figure S2. The *nol12-4* phenotype is rescued by the expression of 35S::NOL12-GFP transgene. The morphological phenotype of 14-day-old and 21-day-old *nol12-4* plants and the 35S::NOL12-GFP/*nol12-4* transgenic line.

Figure S3

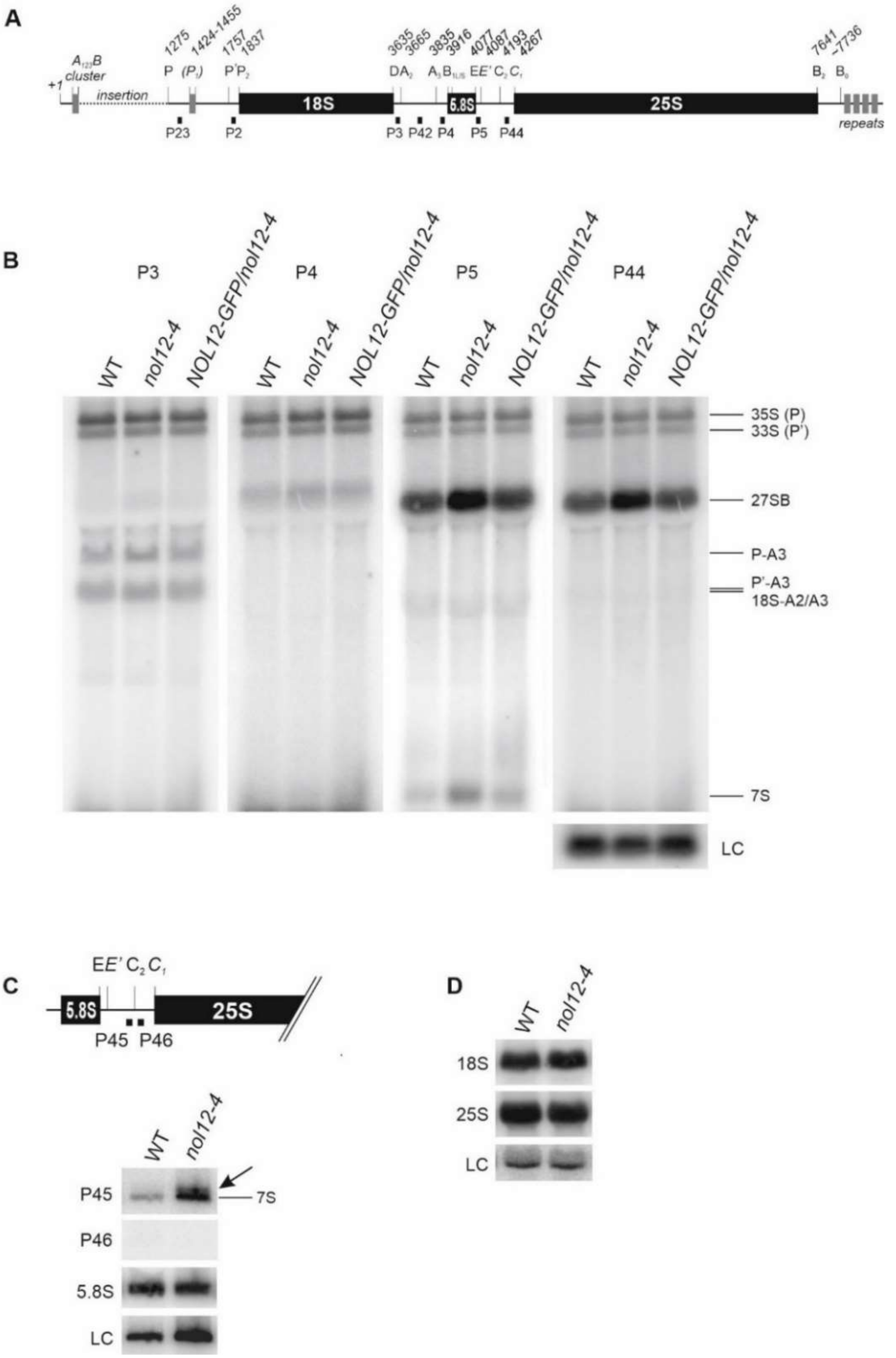

Supplementary Figure S3. The *nol12-4* mutant accumulates pre-rRNAs, but the level of mature rRNAs is not affected. (A) Diagram showing the pre-rRNA structure and probes used for northern blot analysis shown on (B). **(B-D)** Northern blot analysis of pre-rRNA precursors and mature rRNAs in WT, *nol12-4* and 35S::NOL12-GFP/*nol12-4* transgenic line, using probes depicted on (A) and (C). Probes for mature U2 snRNA (B), 7SL RNA (C) and eIF-4A mRNA (D) were used as loading controls (LC). A schematic representation of detected low-molecular-weight pre-rRNAs is shown in (C). The analyses were performed in at least two biological replicates. Full-length blots are included in a Full-length gels and blots section.

Figure S4

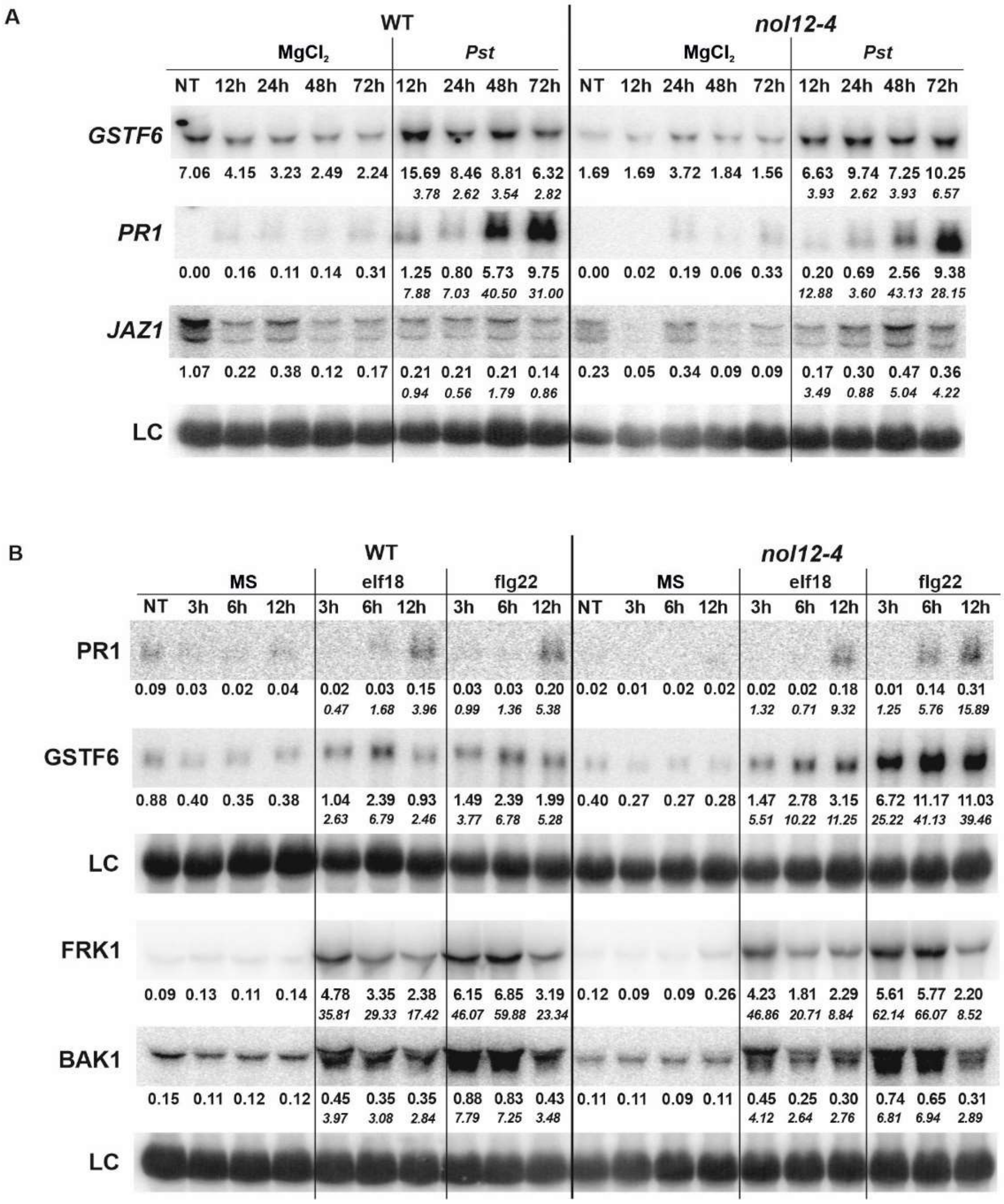

Supplementary Figure S4. The *nol12-4* mutant is sensitive to *Pseudomonas* infection. (**A**) Northern blot analysis of factors involved in response to *Pst* DC3000. Samples were collected from non-treated (NT), control (MgCl2) and infected (*Pst*) WT and *nol12-4* plants at indicated time points. The ratio of transcript level in treated WT and *nol12-4* lines normalized to 18S rRNA loading control (LC) is shown as the main numbers, while the ratio relative to the control is given in italics. (**B**) Northern blot analysis of factors involved in PAMPs response. Samples were collected at indicated time points from non-treated (NT) 14-day-old seedlings, treated with MS (control) or 100 nM of flg22 and elf18. The ratio of transcript level in treated *nol12-4* versus WT normalized to 18S rRNA loading control (LC) is shown as the main numbers, while the ratio relative to the control conditions is given in italics. Analyses were performed in three biological relicates. Full-length blots are included in a Full-length gels and blots section.

Figure S5

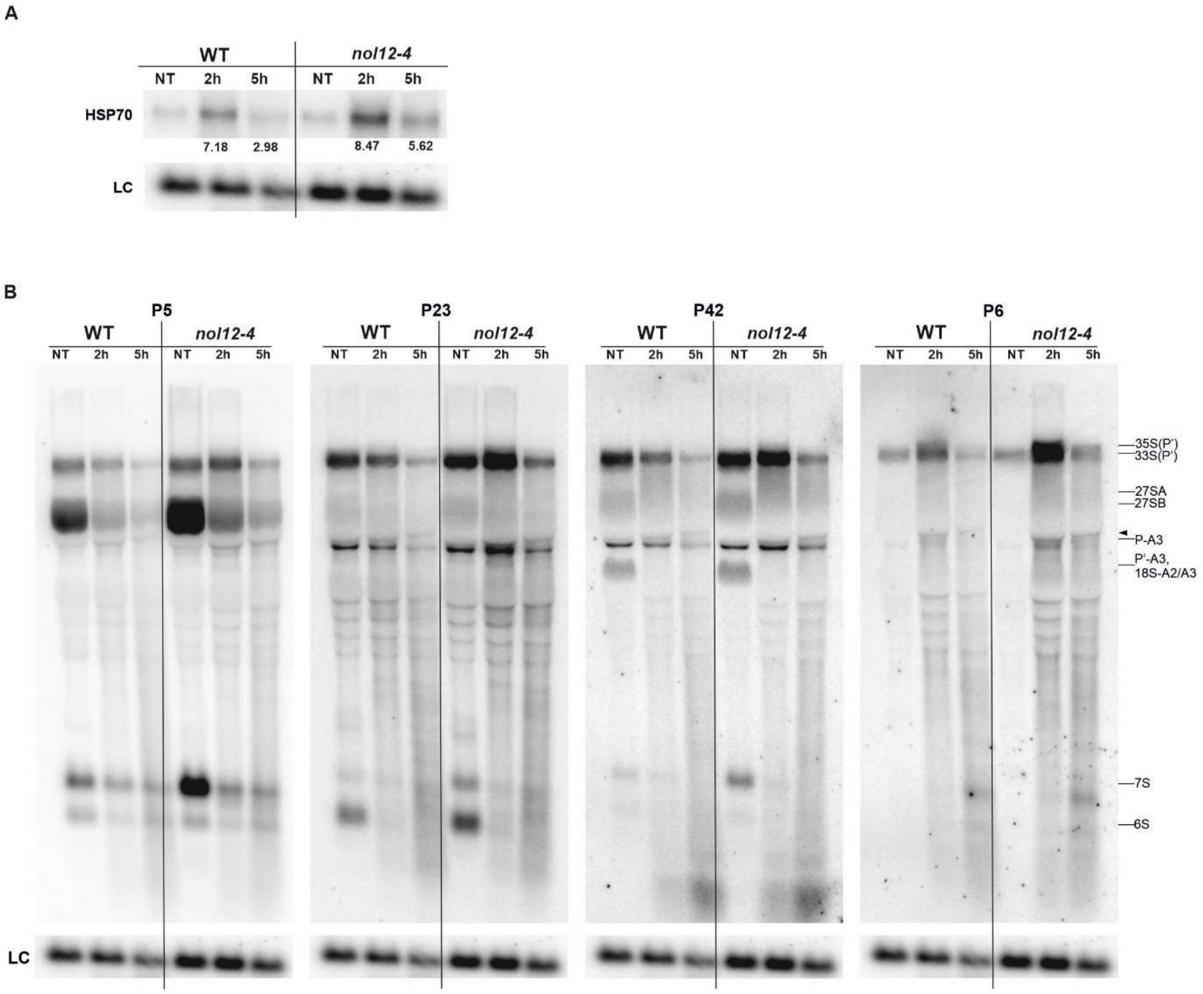

Supplementary Figure S5. Pre-rRNA processing in prolonged heat stress. Northern blot analysis of mRNA **(A)** and rRNA precursors and intermediates **(B)** in WT and the *nol12-4* mutant. Samples were collected from 14-day-old seedlings and treated with high temperatures of 42°C for 2 and 5 h. RNA was separated in 1.1% agarose gels and hybridized with *HSP70* **(A)** and P5, P23, P42, and P6 probes **(B)**. rRNA precursors and intermediates are described on the right. Black arrowhead indicate the P-C2 intermediate. U2 snRNA was used as a loading control (LC). The experiments were performed in three biological replicates. Full-length blots are included in a Full-length gels and blots section.

Figure S6

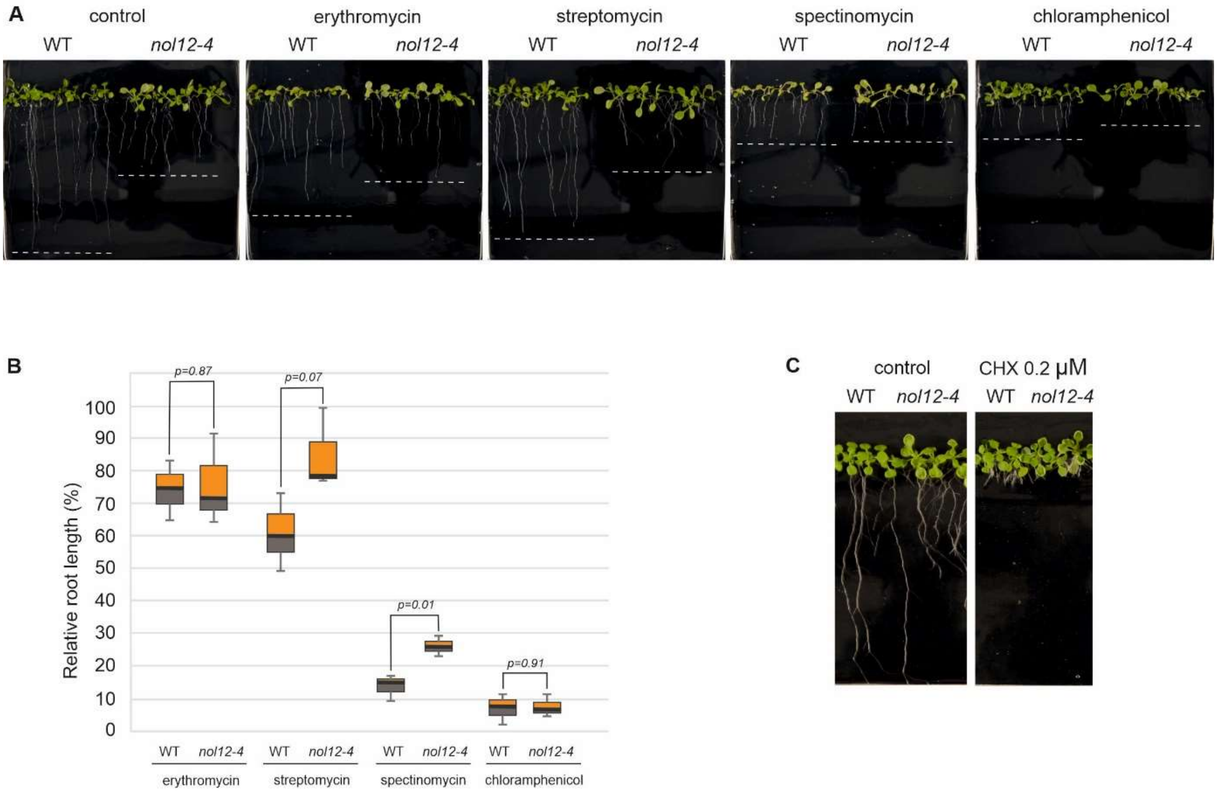

Supplementary Figure S6. The *nol12-4* mutant presents altered sensitivity to ribosome-targeting antibiotics. Analysis of root growth of WT and *nol12-4* plants grown on media containing indicated antibiotics in the following concentrations: erythromycin 30 µg/ml, streptomycin 30 µg/ml, spectinomycin 50 µg/ml and chloramphenicol 10 µg/ml. **(A)** Root length of 19-day-old seedlings grown on vertical plates. **(B)** Box plot for relative root length of WT and *nol12-4* plants grown on plates with indicated antibiotics, normalized to respective plant lines grown on antibiotic-free medium. Dark horizontal lines represent the median, with the box representing the 25th and 75th percentiles and the whiskers the 1.5 IQR limits. *p-values* by ANOVA test among WT and *nol12-4* are depicted. For each antibiotic treatment, at least 14 plants were measured. **(C)** WT and *nol12-4* plants grown on plates containing 0.2 µM cycloheximide (CHX).

SUPPLEMENTARY TABLES

Supplementary Table S1. Oligonucleotides used in this work.

Name	Sequence	Additional description
**primers used for genotyping**		
GABI-TDN	5'-CCCATTTGGACGTGAATGTAGACAC	for GABI mutant *nol12-4*
GFP-R	5'-TCGCCCTCGAACTTCACCTC	for 35S::NOL12-GFP/*nol12-4* line
LBb1.3	5'-TAGAGTGGACAACTCGGGTTCC	for SALK mutant lines
NOL12-F	5'-GTTGAGCCTGGTTCTTCTTG	
NOL12-R	5'-GTGAAAACCAGTCACAAAATCC	
LB3	5'-TAGCATCTGAATTTCATAACCAATCTCGATACAC	for SAIL mutant *nol12-3*
NOL12-A	5'-GGCTTGTTTTGATTAAACCTCG	
**primers for plasmid construction**		
F	5'-GGGGACAAGTTTGTACAAAAAAGCAGGCTTAATGACGGGAGGCTTGCAT	
R	5'-GGGGACCACTTTGTACAAGAAAGCTGGGTATCGCGAAGTCTTGATCCC	
**oligonucleotide probes**		
P2	5'-CATCGATCACGGCAATTCCCCGC	(Zakrzewska-Placzek et al., 2010)
P3	5'-GGTCGTTCTGTTTTGGACAGGTATC	(Zakrzewska-Placzek et al., 2010)
P4	5'-CGTTTTAGACTTCAGTTCGCAG	(Zakrzewska-Placzek et al., 2010)
P5	5'-GCAAAGGATGGTGAGGGACGACG	(Zakrzewska-Placzek et al., 2010)
P6	5'-CGTTAAGGAGCTGTTGCTTTGTTAGTGTAG	(Zakrzewska-Placzek et al., 2010)
P7	5'-GATTCTGCAATTCACACCAAGTATC	used for detection of 5.8S rRNA and primer extension for 5.8S 5' end (Zakrzewska-Placzek et al., 2010)
P8	5'-CTCCGCTTATTGATATGCTTAAAC	used for detection of 25S rRNA and primer extension for 25S 5' end (Zakrzewska-Placzek et al., 2010)
P10	5'-CATATGACTACTGGCAGGATCAACC	for 18S rRNA (Zakrzewska-Placzek et al., 2010)
P13	5'-ACTGGGCAGCCCAGAAACATGC	for 7SL RNA (loading control, Zakrzewska-Placzek et al., 2010)
P23	5'-GTTCCAACTACTCTACCGAAGTAC	(Zakrzewska-Placzek et al., 2010)
P42	5'-CCACGGATCCGGCGGGCAAGG	(Zakrzewska-Placzek et al., 2010)
P44	5'-GGACTTTGGGTCATCTACAGCTTC	(Zakrzewska-Placzek et al., 2010)

P45	5'-CATGTCGGTACGCTCCAGGCG	
P46	5'-CCGATAAAATGTAATGGATCAAGTTC	
U2	5'-AATAGAGTTAATATCGTGTGGG	
**primers used for random primed probes**		
BAK1-F	5'-AGTGGAGCAGCTAATCCAAGTGGC	
BAK1-R	5'-GGTAAGAAAAGAAACCTGACGCACGG	
EIF4A1-F	5'-TCATGAGAGCTTTGATGCCATGG	
EIF4A1-R	5'-GATGAGAACACGGGAGGAACCAG	
FRK1-F	5'-TCGGATTCGGCGTTTGTTGATTC	
FRK1-R	5'-CTCTCGTTTCGCGCTGTTTCTGC	
GSTF6-F	5'-CTCAACTGGCAAGGACATGGCG	
GSTF6-R	5'-CATTCAAATCAAACACTCGGCAGCAG	
HSP70-F	5'-AGGACAGCTTGTGAGCGGGC	
HSP70-R	5'-GCGGTACACCTCGTGGAGCAG	
JAZ1-F	5'-ACGTCAGCCGACAACAACCATGAG	
JAZ1-R	5'-AGGGTTTGAAGACGCTTTGGCTGG	
NOL12-F2	5'-GACGGGAGGCTTGCATAATG	
NOL12-R2	5'-CTATCGCGAAGTCTTGATCCC	
PR1-F	5'-TCCCTCGAAAGCTCAAGATAGCCCAC	
PR1-R	5'-GCTTCTCGTTCACATAATTCCCACGAG	
PR2-F	5'-CAATGCAGAACATCGAGAACGCGG	
PR2-R	5'-CACCACGATTTCCAACGATCCGCC	
PR5-F	5'-CGCCGGTCAAGGACCCAAGC	
PR5-R	5'-ACAGGCACTCTTGCAGGCCAC	
VSP2-F	5'-GACTTCGACACGGTGCCCGC	
VSP2-R	5'-GGTCACGCCAGCAGCTTCGAG	
WRKY25-F	5'-CATCGGTTAATGGGAGGCGCTTG	
WRKY25-R	5'-GAGCGACGTAGCGCGGTTGG	

Supplementary Table S2. Characterization of genes involved in pathogen response in *nol12-4* plants.

ID	Name	log2 FoldChange	**Description** (modified after www.arabidopsis.org)
**AT1G02450**	NIMIN1	-4,74	NIMIN1 modulates PR gene expression by forming a ternary complex with NPR1 and TGA factors upon SAR induction. The complex binds to a positive regulatory cis-element of the PR-1 promoter, leading to PR-1 gene induction. NIMIN1 decreases transcriptional activation, possibly through its EAR motif, which results in fine-tuning of PR-1 gene expression.
**AT5G40990**	GLIP1	-4,73	Component of plant resistance. Contains lipase signature motif and GDSL domain. Directly interferes with the fungal infection by acting on fungal cell walls through its action as a antimicrobial compound. Critical for both local and systemic resistance responses in the incompatible interaction with *Alternaria brassicicola* in the ethylene-dependent pathway.
**AT2G30750**	CYP71A12	-4,42	Putative cytochrome P450; together with CYP71A13 produces dihydrocamalexic acid (DHCA), the precursor to the defense-related compound camalexin, which accumulates in the intercellular space and contributes to the resistance of Arabidopsis to *P. syringae* without inhibiting bacterial growth.
**AT1G54040**	ESP	-3,97	Epithiospecifier protein, interacts with WRKY53. Involved in pathogen resistance and leaf senescence.
**AT4G11170**	RMG1	-3,76	Encodes RMG1 (Resistance Methylated Gene 1), a NB-LRR disease resistance protein with a Toll/interleukin-1 receptor (TIR) domain at its N terminus. RMG1 is expressed at high levels in response to flg22. Expression of RMG1 is controlled by DNA methylation in its promoter region, which is constitutively demethylated by active DNA demethylation mediated by the DNA glycosylase ROS1.
**AT2G19190**	FRK1	-3,50	Encodes a receptor-like protein kinase that is involved in early defense signaling. Expression of this gene is strongly induced during leaf senescence. A target of the WRKY6 transcription factor.
**AT4G23150**	CRK7	-3,45	Encodes a cysteine-rich receptor-like protein kinase. Involved in defense response to bacterium.
**AT4G23210**	CRK13	-2,95	Encodes a Cysteine-rich receptor-like kinase (CRK13). Overexpression of CRK13 leads to hypersensitive response cell death, and induces defense against pathogens by causing increased accumulation of salicylic acid.
**AT4G23280**	CRK20	-2,93	Encodes a cysteine-rich receptor-like protein kinase. Involved in defense response to bacterium.
**AT4G14400**	ACD6	-2,66	Encodes a protein with putative ankyrin and transmembrane regions. It is involved in resistance to *P. syringae*.
**AT2G40750**	WRKY54	-2,30	Member of WRKY Transcription Factor Group III. Together with WRKY70 positively regulates SARD1 and CBP60g expression in plant immunity.

**AT5G01560**	LECRK-VI.4	-2,30	Encodes LecRKA4.3, a member of the lectin receptor kinase subfamily A4, which function redundantly in the negative regulation of ABA response in seed germination. Involved in defense response to bacterium.
**AT5G60900**	RLK1	-2,18	Encodes a receptor-like protein kinase. Involved in defense response to bacterium.
**AT5G44910**	TIR	-2,16	Toll-Interleukin-Resistance (TIR) domain family protein. Involved in defense response to bacterium.
**AT3G57700**	ZRK10	-2,15	Protein kinase superfamily protein. Involved in defense response to bacterium.
**AT1G19610**	PDF1.4	-2,14	Predicted to encode a PR (pathogenesis-related) protein. Belongs to the plant defensin (PDF) family. Involved in defense response.
**AT1G52940**	PAP5	-2,06	Encodes a purple acid phosphatase that is induced under prolonged phosphate) starvation and is required for maintaining basal resistance against *P. syringae* and *B. cinerea.*
**AT4G23130**	CRK5	-2,06	Encodes a cysteine-rich receptor-like protein kinase. Involved in defense response to bacterium and salicylic acid.
**AT2G43590**	PR3	-2,04	PR-3 like gene that is induced by pathogen infection.
**AT3G25882**	NIMIN2	-1,87	Encodes a kinase that physically interacts with NPR1/NIM1. Involved in regulation of systemic acquired resistance.
**AT1G02930**	GSTF6	-1,80	Encodes glutathione transferase belonging to the phi class of GSTs.
**AT4G12470**	AZI1	-1,72	Encodes AZI1 (AZELAIC ACID INDUCED 1), involved in the priming of salicylic acid induction and systemic immunity triggered by pathogen or azelaic acid. Targeting if AZI1 to chloroplasts is increased during SAR induction and that localization requires the PRR domain. It is involved in the uptake and movement of the azelaic acid signal. AZI1 is strongly induced by flg22 MAMP treatment that also increases its enrichment in the plastid fraction.
**AT5G46050**	PTR3	-1,67	Encodes a di- and tri-peptide transporter involved in responses to wounding, virulent bacterial pathogens, and high NaCl concentrations.
**AT4G26090**	RPS2	-1,34	Encodes a plasma membrane protein with leucine-rich repeat, leucine zipper, and P loop domains that confers resistance to *P. syringae* infection by interacting with the avirulence gene avrRpt2. RPS2 protein interacts directly with plasma membrane associated protein RIN4 and this interaction is disrupted by avrRpt2.
**AT1G51850**	SIF2	-1,29	Malectin-like receptor-like kinase involved in MAMP mediated stomatal immunity. Interacts with BAK1/FLS2 signaling complex and subsequently phosphorylates and activates SLAC1.
**AT4G21400**	CRK28	-1,10	Encodes a cysteine-rich receptor-like protein kinase CRK28 that associates with BAK1 or FLS2.
**AT4G21410**	CRK29	-1,10	Encodes a cysteine-rich receptor-like protein kinase.
**At4g23190**	CRK11	-1,06	Encodes putative receptor-like protein kinase that is induced by *R. solanacearum, P. syringae* and salicylic acid. Involved in defense response to bacterium.

**AT5G48380**	BIR1	-1,01	Encodes a BAK1-interacting receptor-like kinase named BIR1. Negatively regulates multiple plant resistance signaling pathways.
**AT1G33970**	IAN9	-0,85	IAN9 is a member of a small family of proteins. Its expression is repressed upon pathogen infection and loss of function mutants show increased resistance to bacterial pathogens.
**AT4G23270**	CRK19	-0,83	Encodes a cysteine-rich receptor-like protein kinase. Involved in defense response to bacterium and salicylic acid.
**AT3G08510**	PLC2	-0,81	Phosphoinositide-specific phospholipase C (PI-PLC), catalyzes hydrolysis of phosphatidylinositol 4,5-bisphosphate into inositol 1,4,5-trisphosphate and diacylglycerol. Involved in auxin biosynthesis and signaling. It also regulates MAMP-triggered immunity by modulating ROS production.
**AT4G03960**	PFA-DSP4	-0,80	Encodes an atypical dual-specificity phosphatase involved in the negative regulation of defense response to *P. syringae*.
**AT1G12220**	RPS5	-0,80	Resistance gene, mediates resistance against *P. syringae*. Contains a putative nucleotide binding site composed of kinase-1a (or P-loop), kinase-2a, and putative kinase-3a domains, 13 imperfect leucine-rich repeats, and a potential leucine zipper. Confers resistance to *P. syringae* strains that express avrPphB.
**AT1G70530**	CRK3	-0,58	Encodes a cysteine-rich receptor-like protein kinase CRK3.
**AT1G30135**	JAZ8	3,99	Jasmonate-zim-domain protein 8, involved in regulation of defense response.
**AT3G22275**	JAZ13	6,96	Encodes a non-TIFY JAsmonate ZIM-domain (JAZ13) protein with a Ser-rich C-terminal tail that is a site for phosphorylation that interacts with the bHLH transcription factor MYC2 and the co-repressor TOPLESS and acts as a repressor of JA signaling.

Full-length gels and blots.

Original full-size images of gels and blots cropped on Figures.

Raw data for Fig. 1C

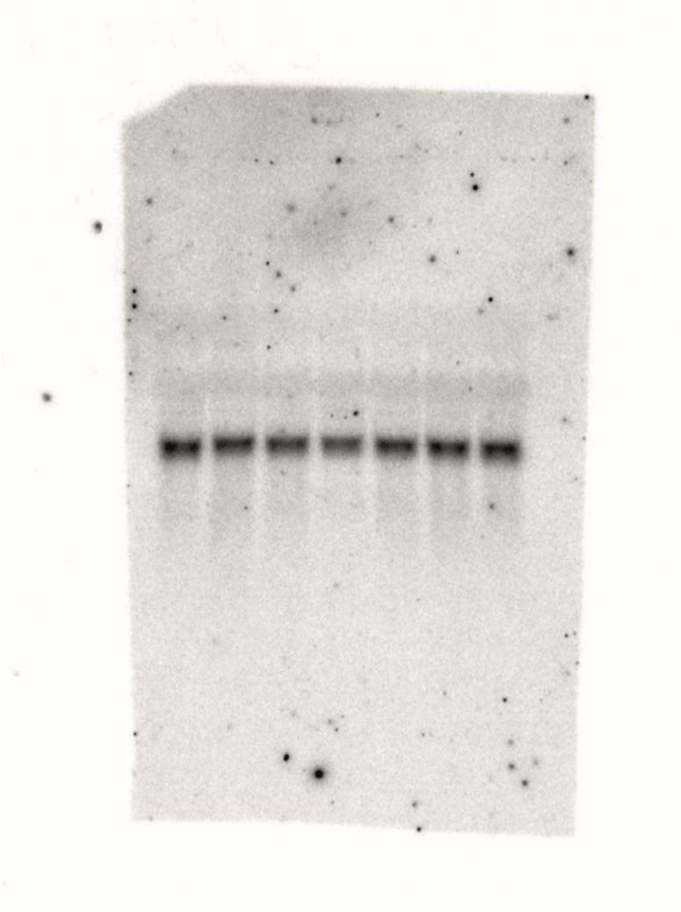

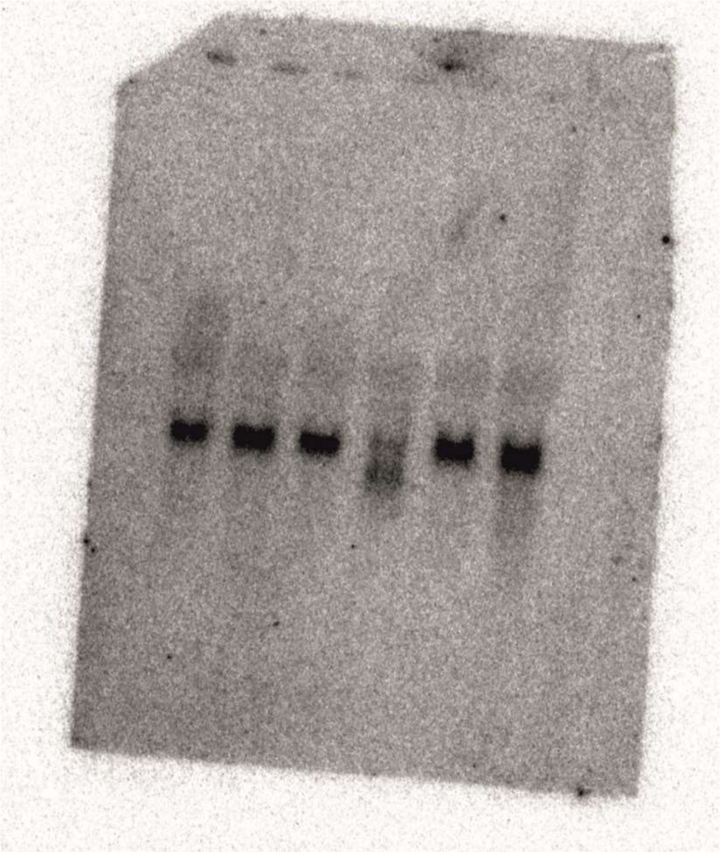

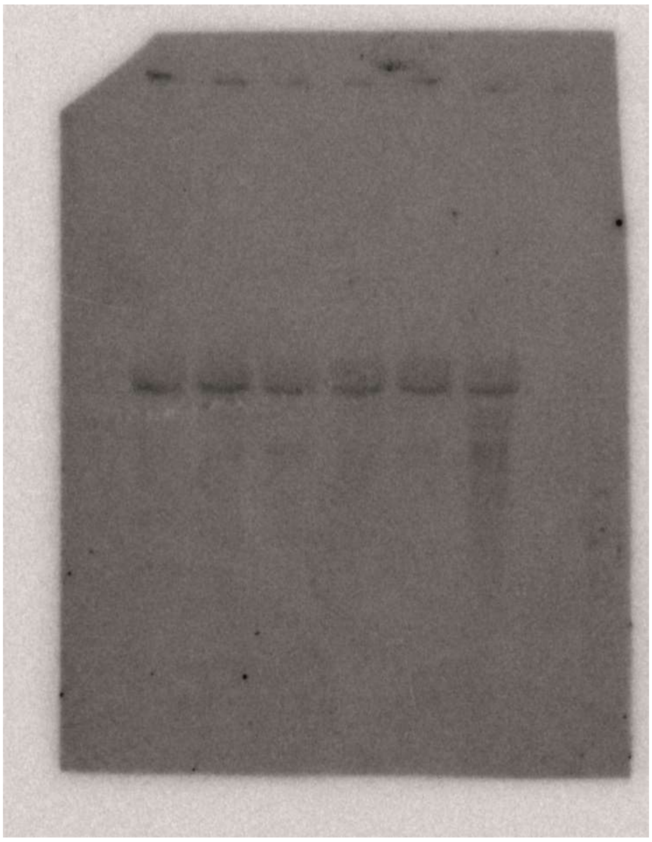

Raw data for Fig. 3B

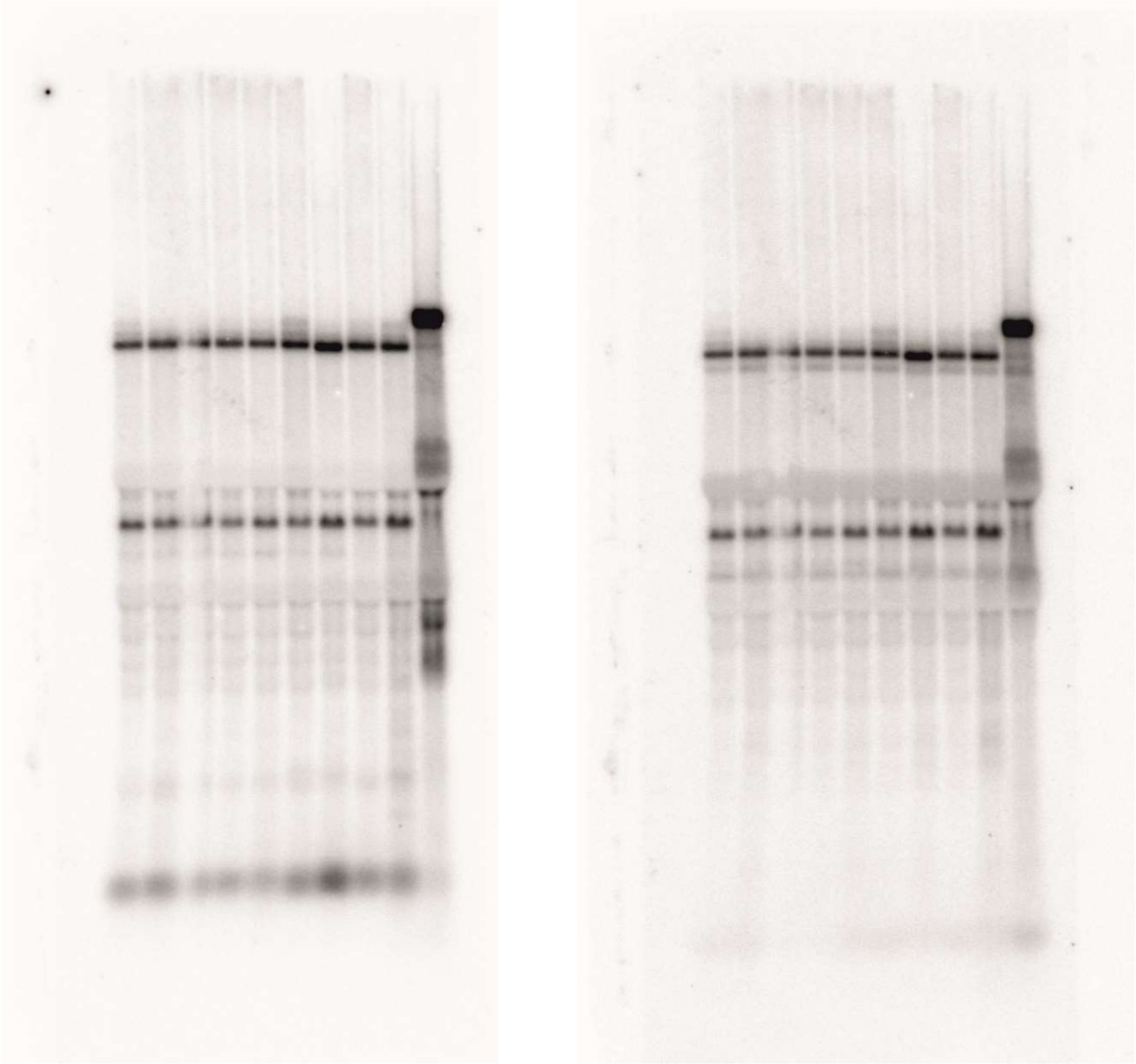

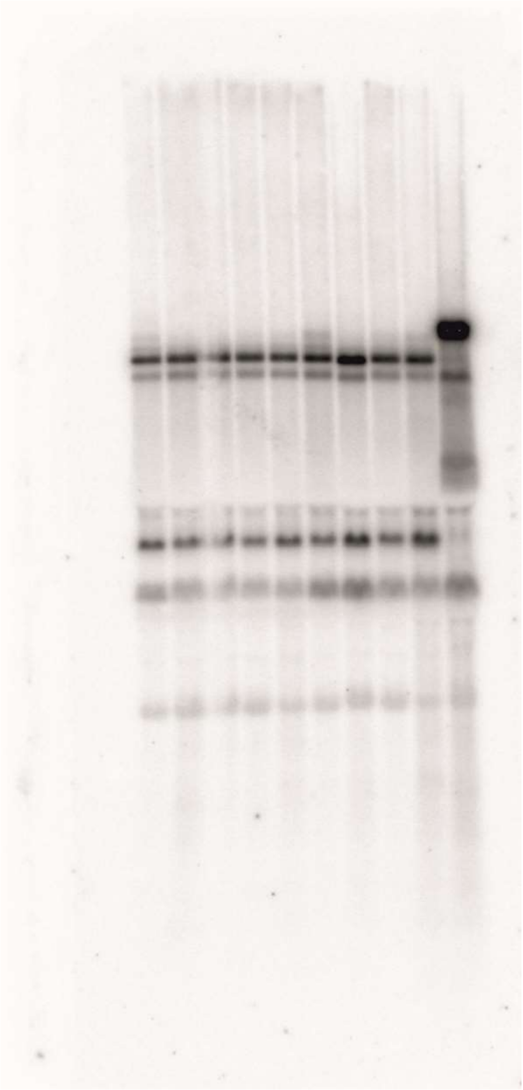
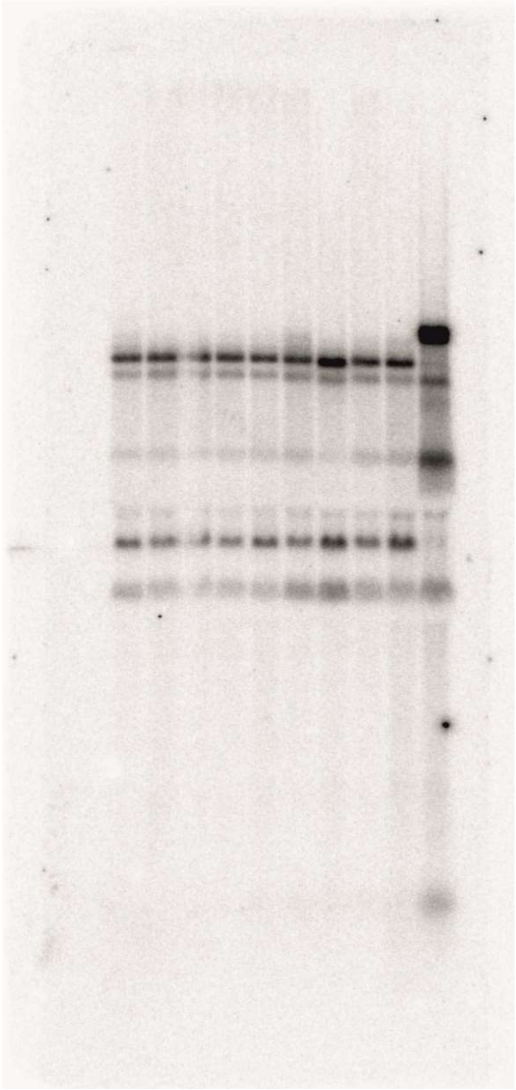

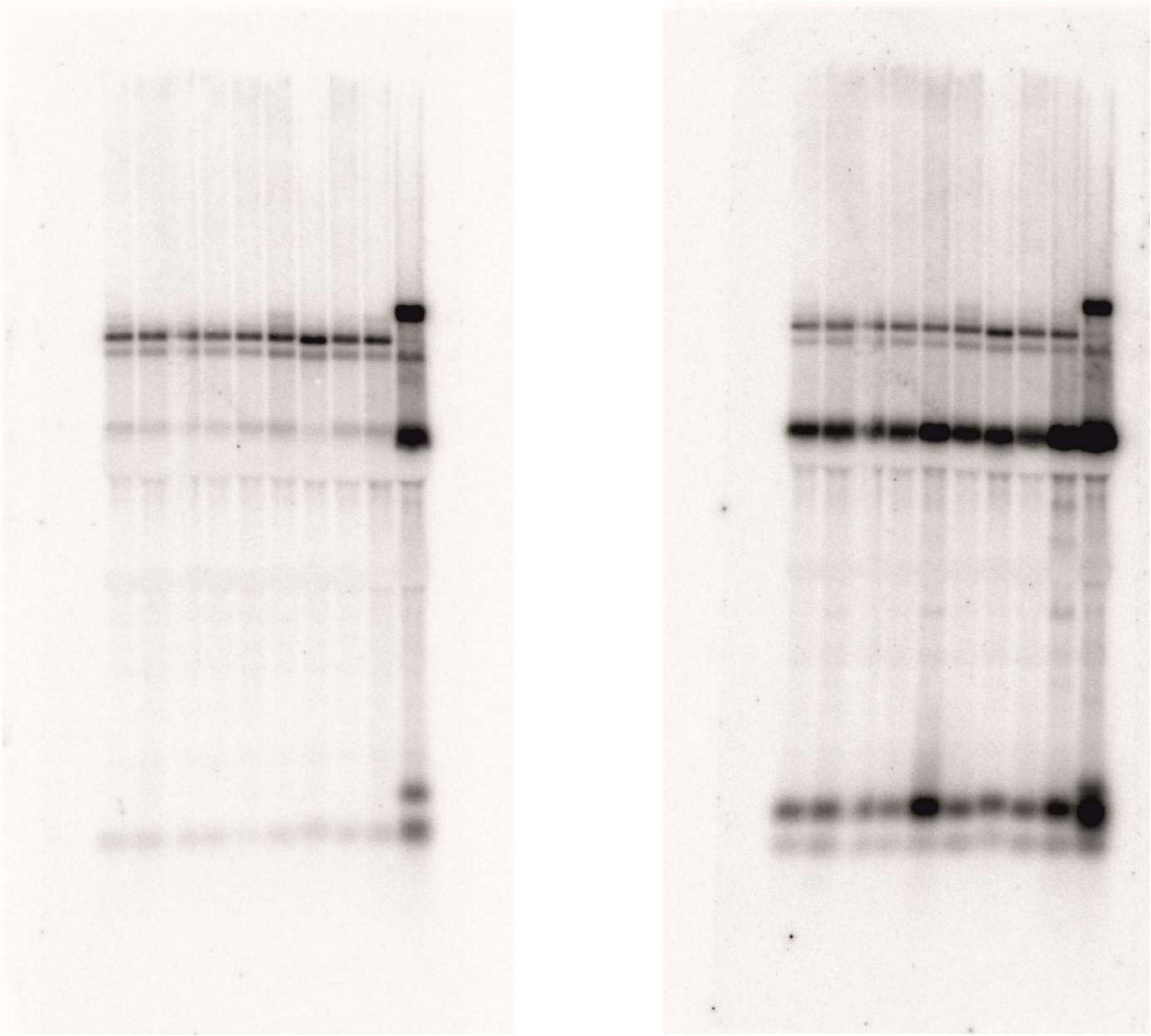

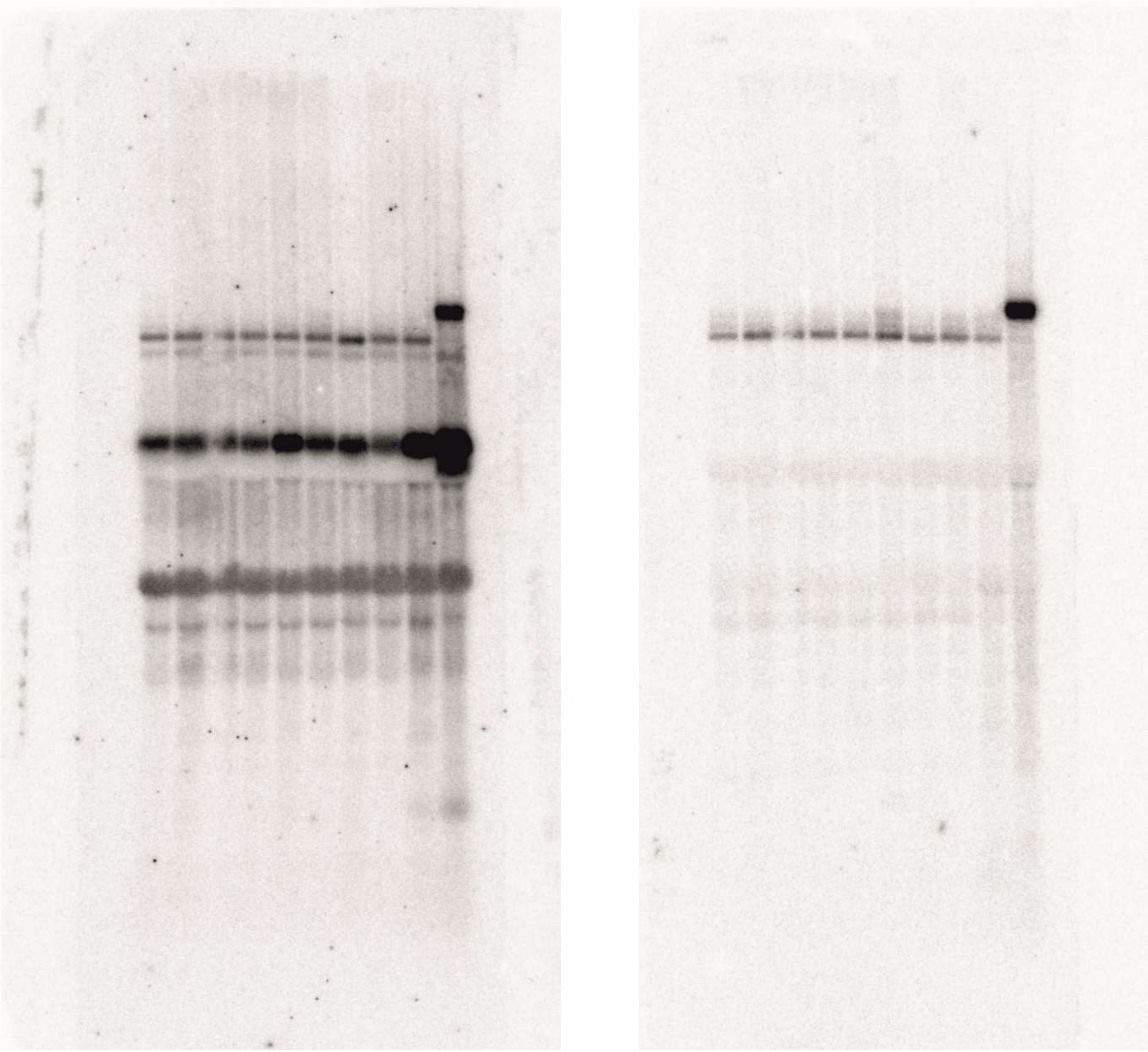

Raw data for Fig. 3C

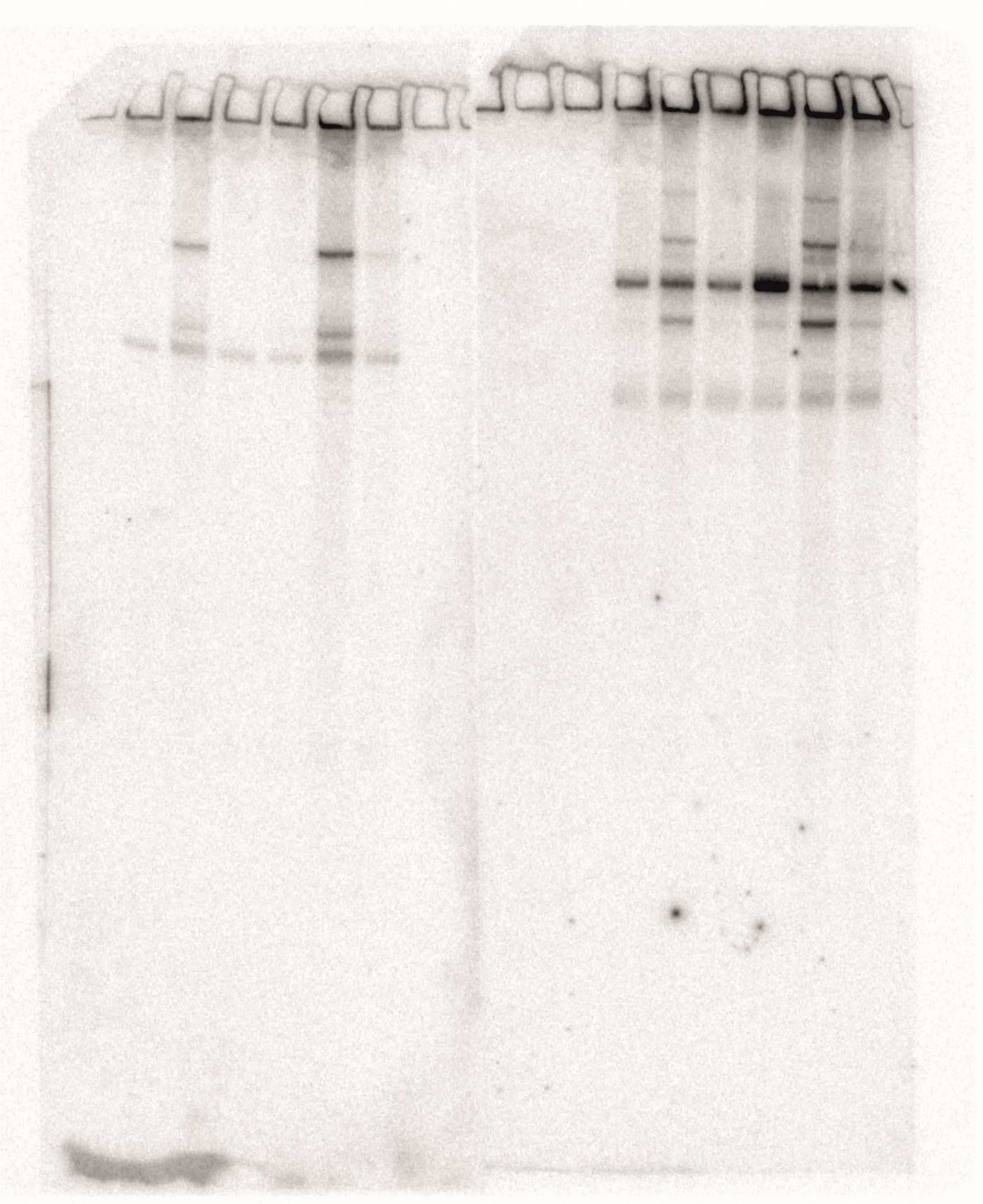

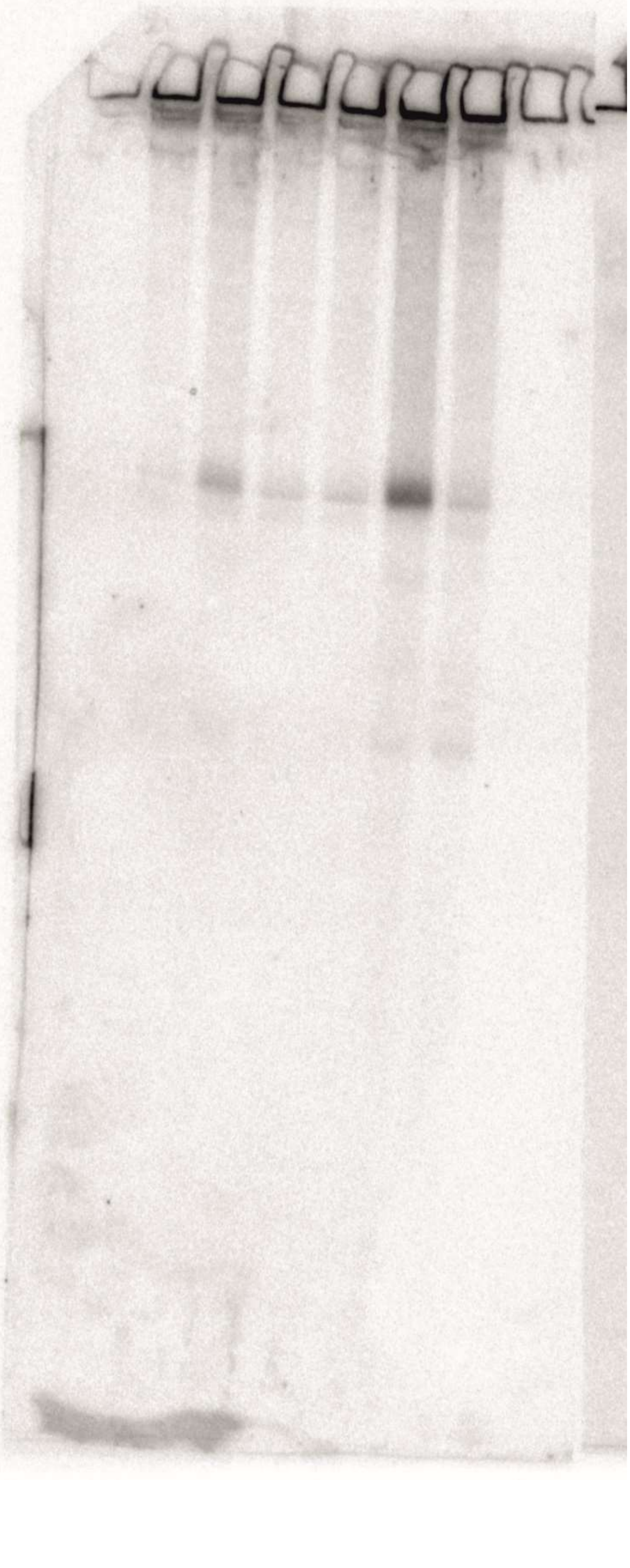

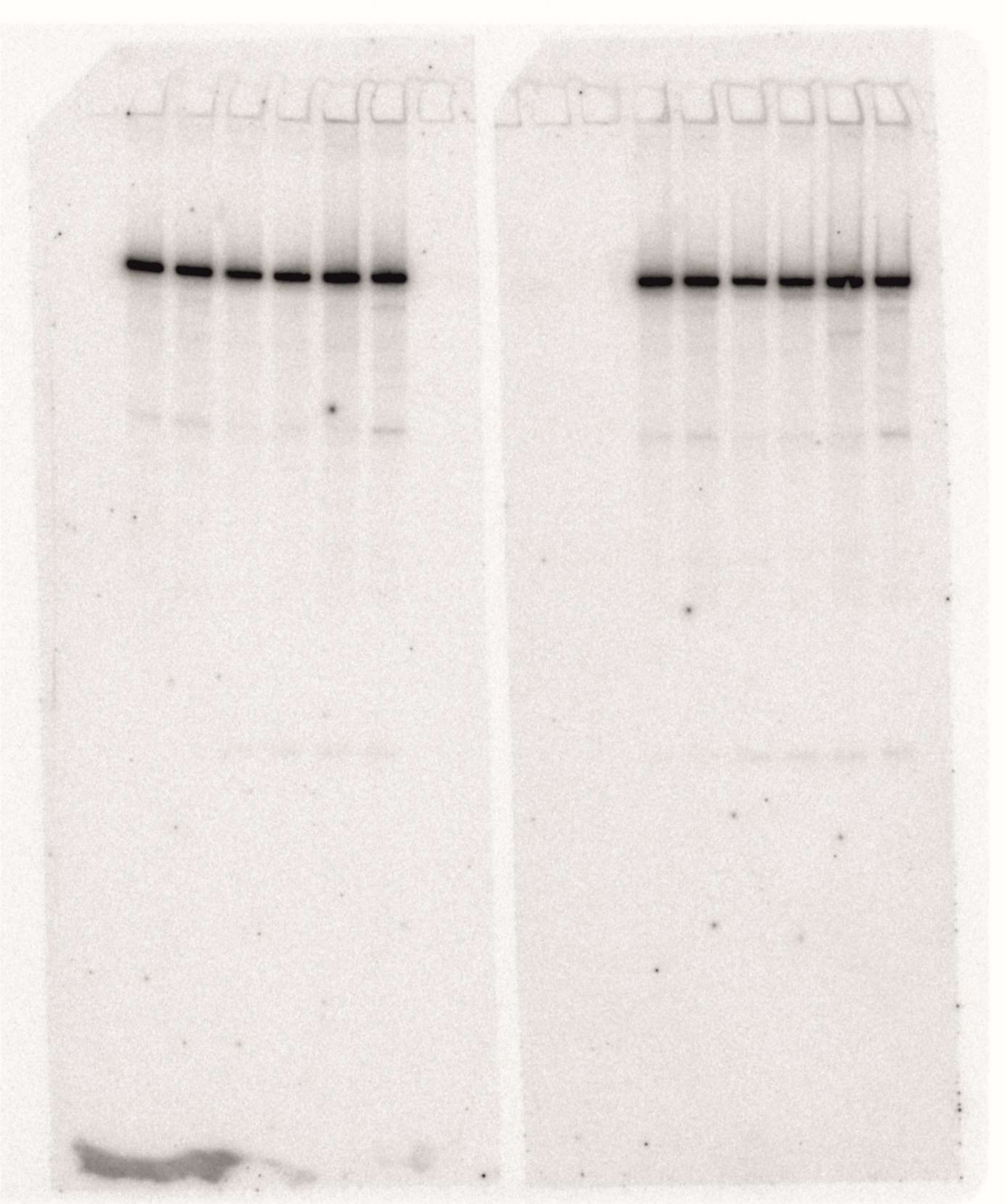

Raw data for Fig. 3D

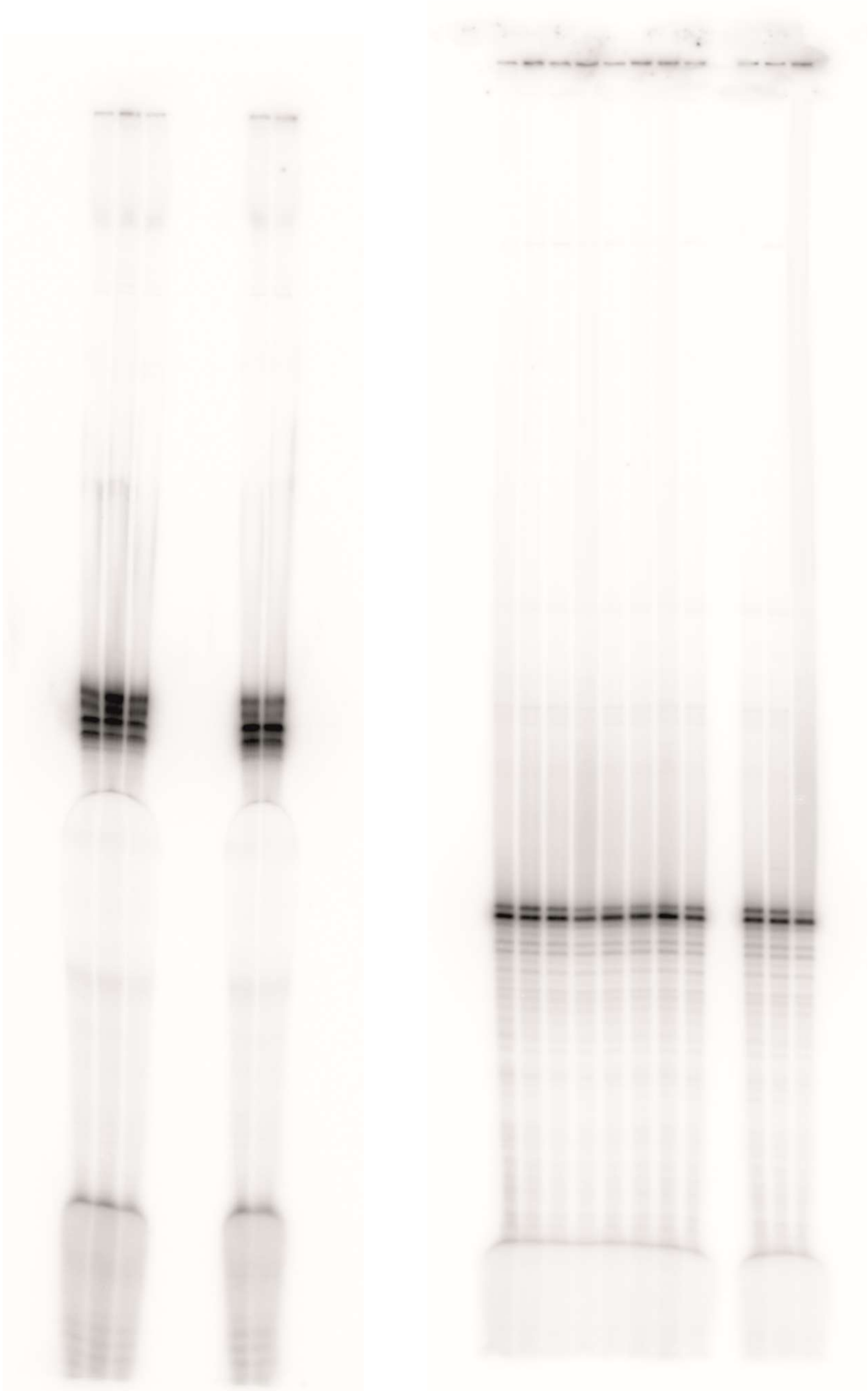

Raw data for Fig. 4B

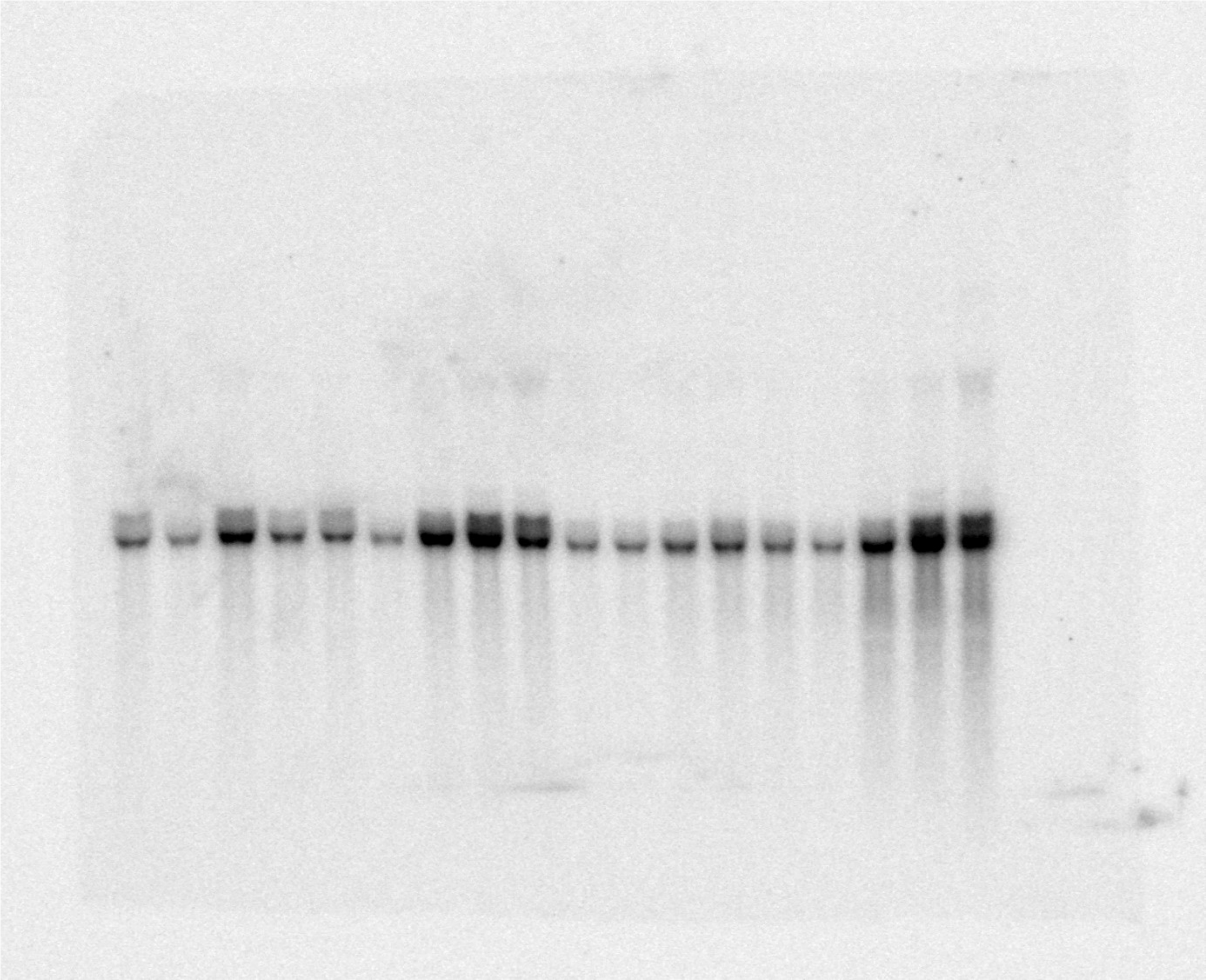

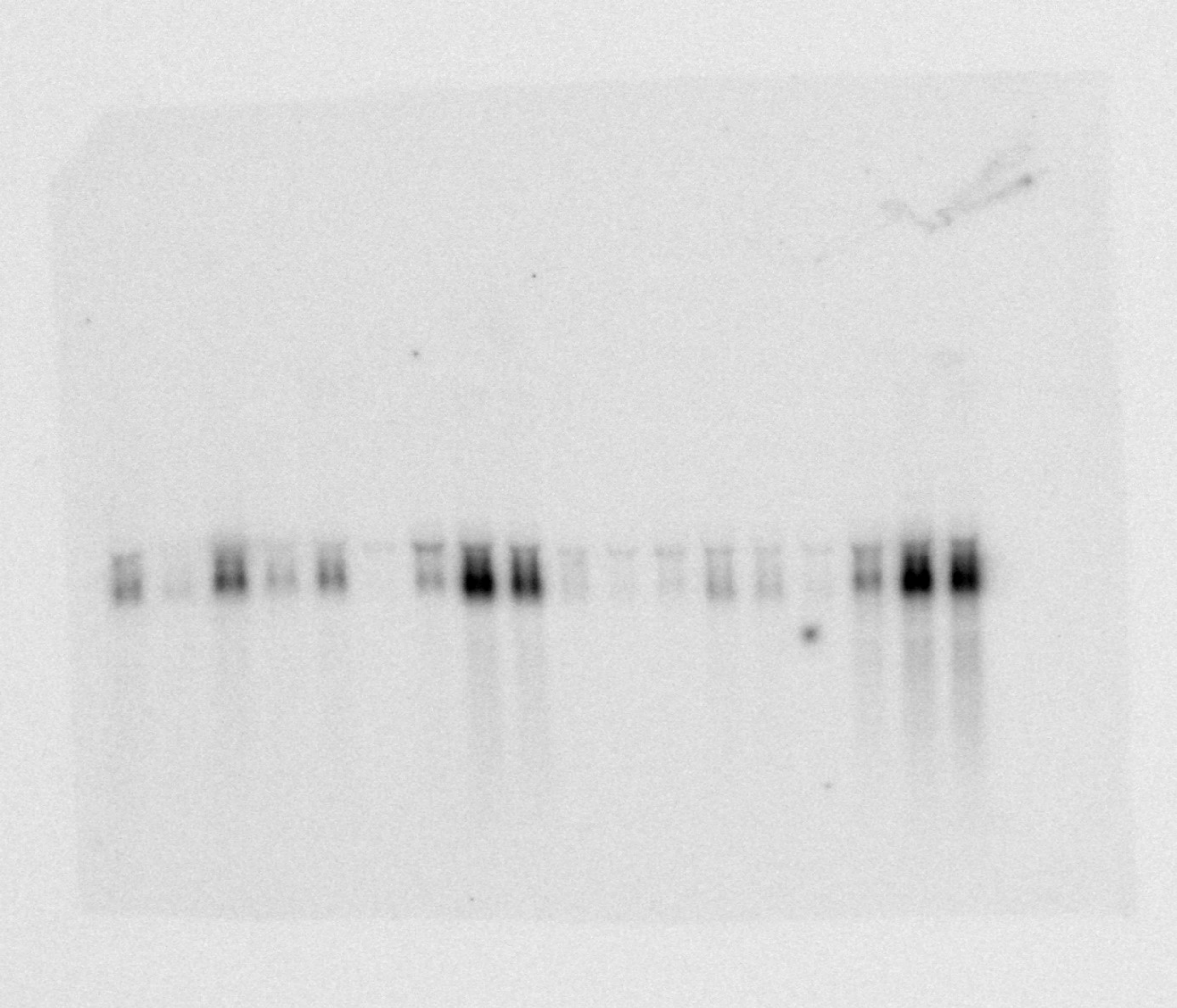

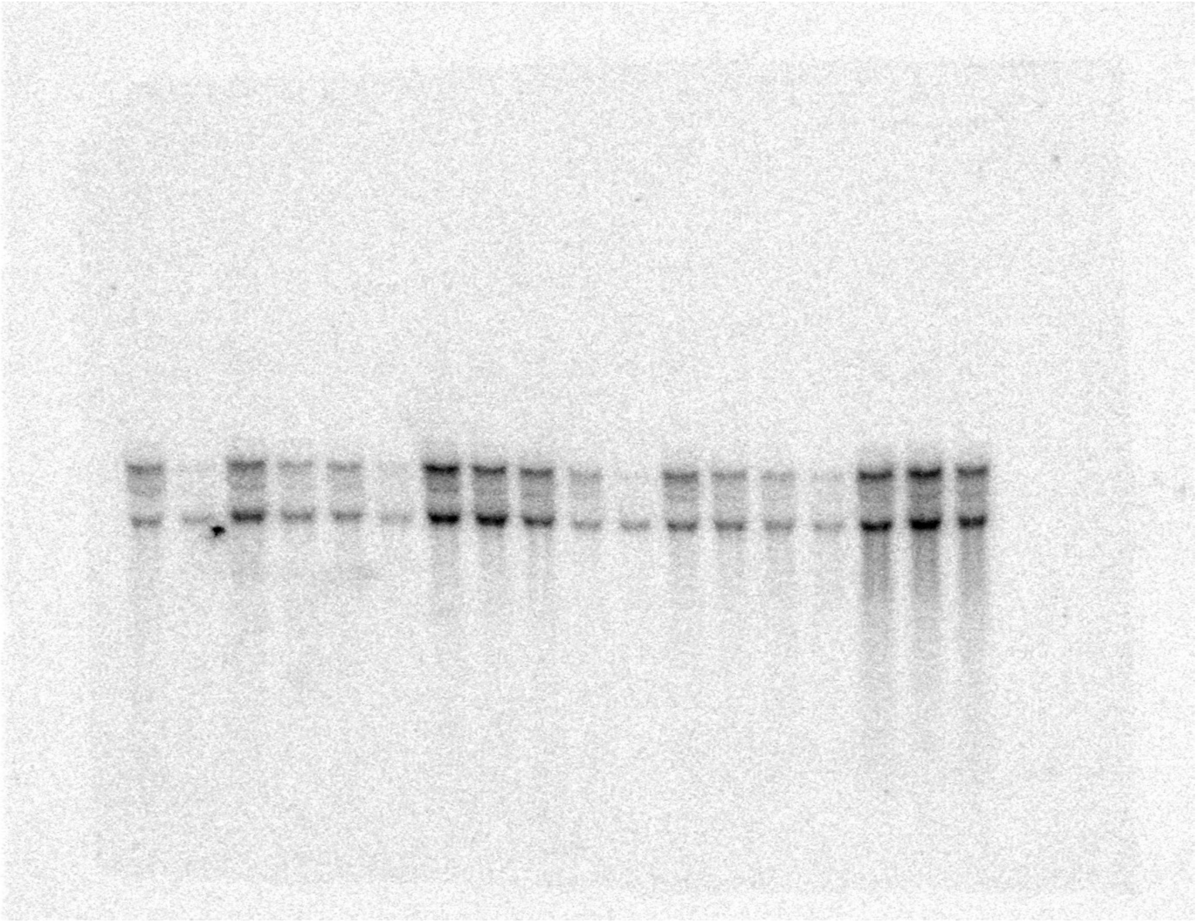

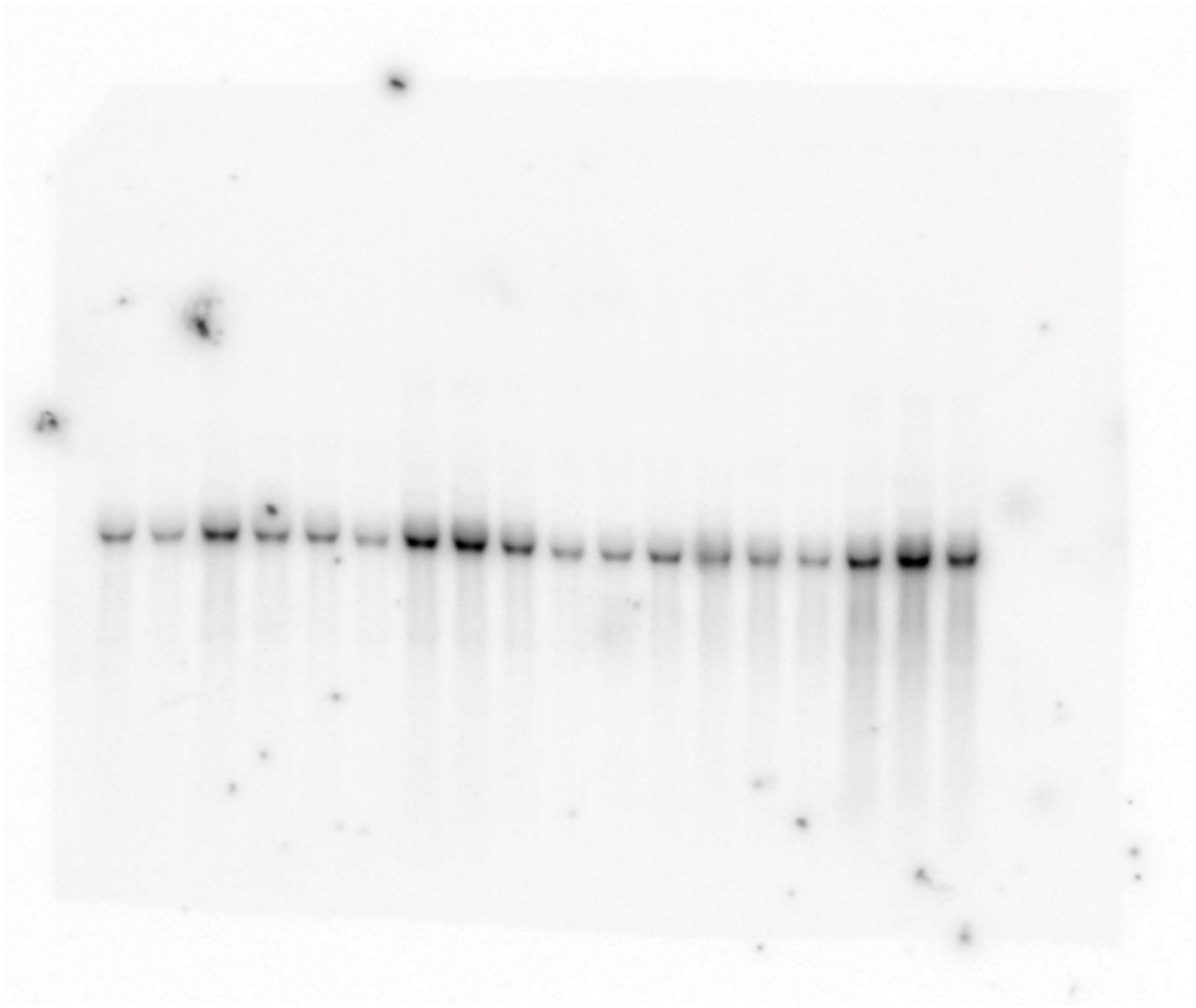

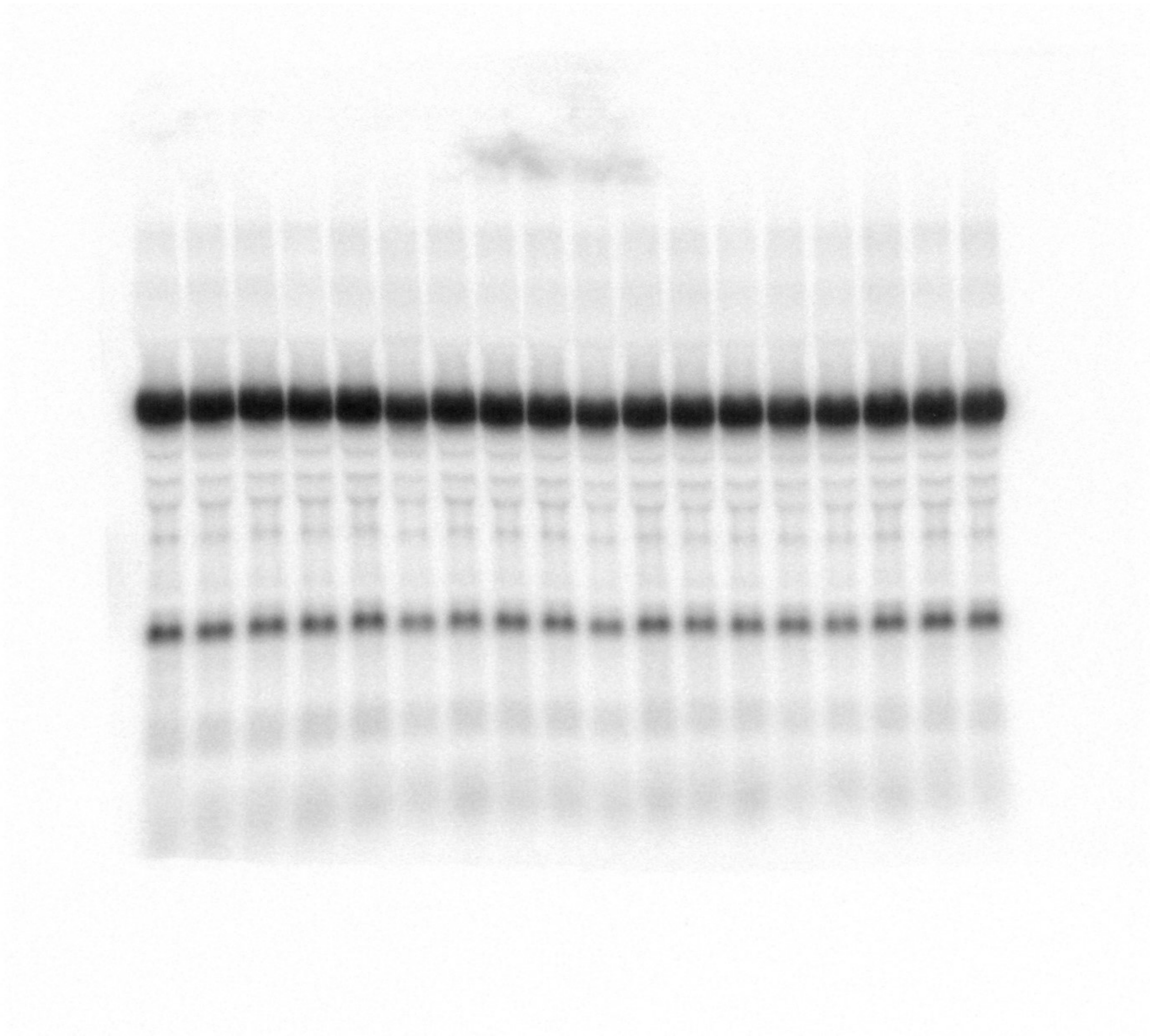

Raw data for Fig. 4C

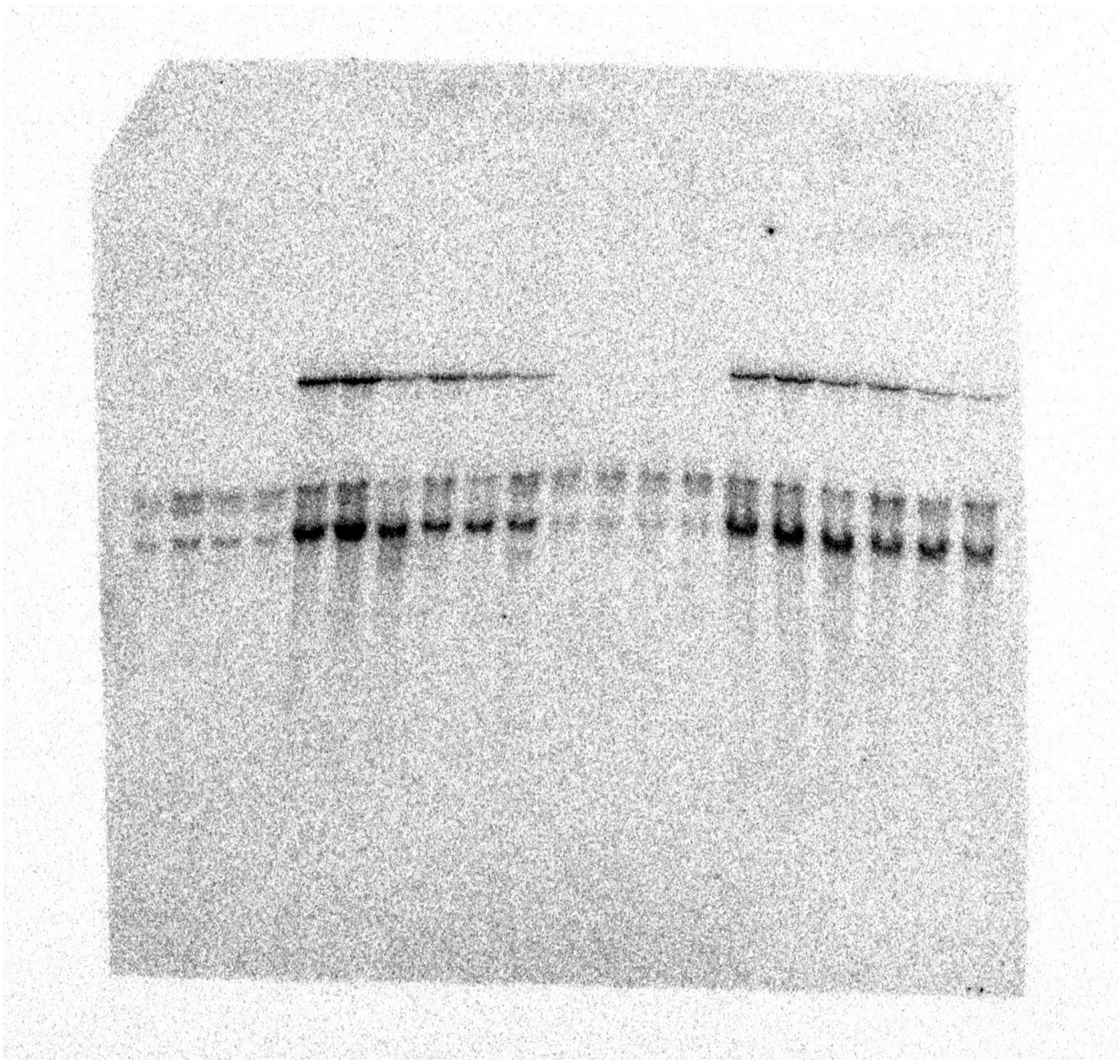

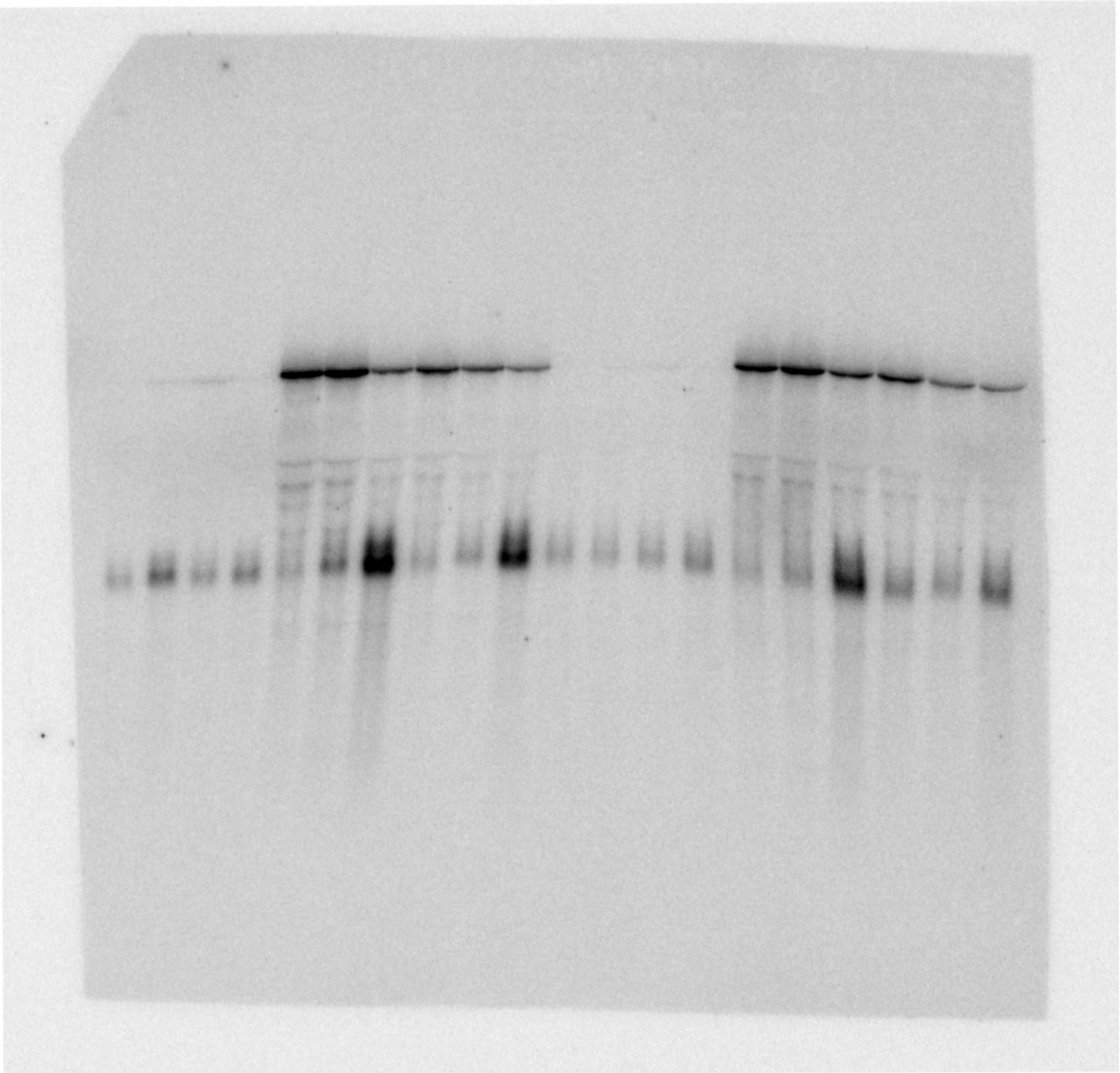

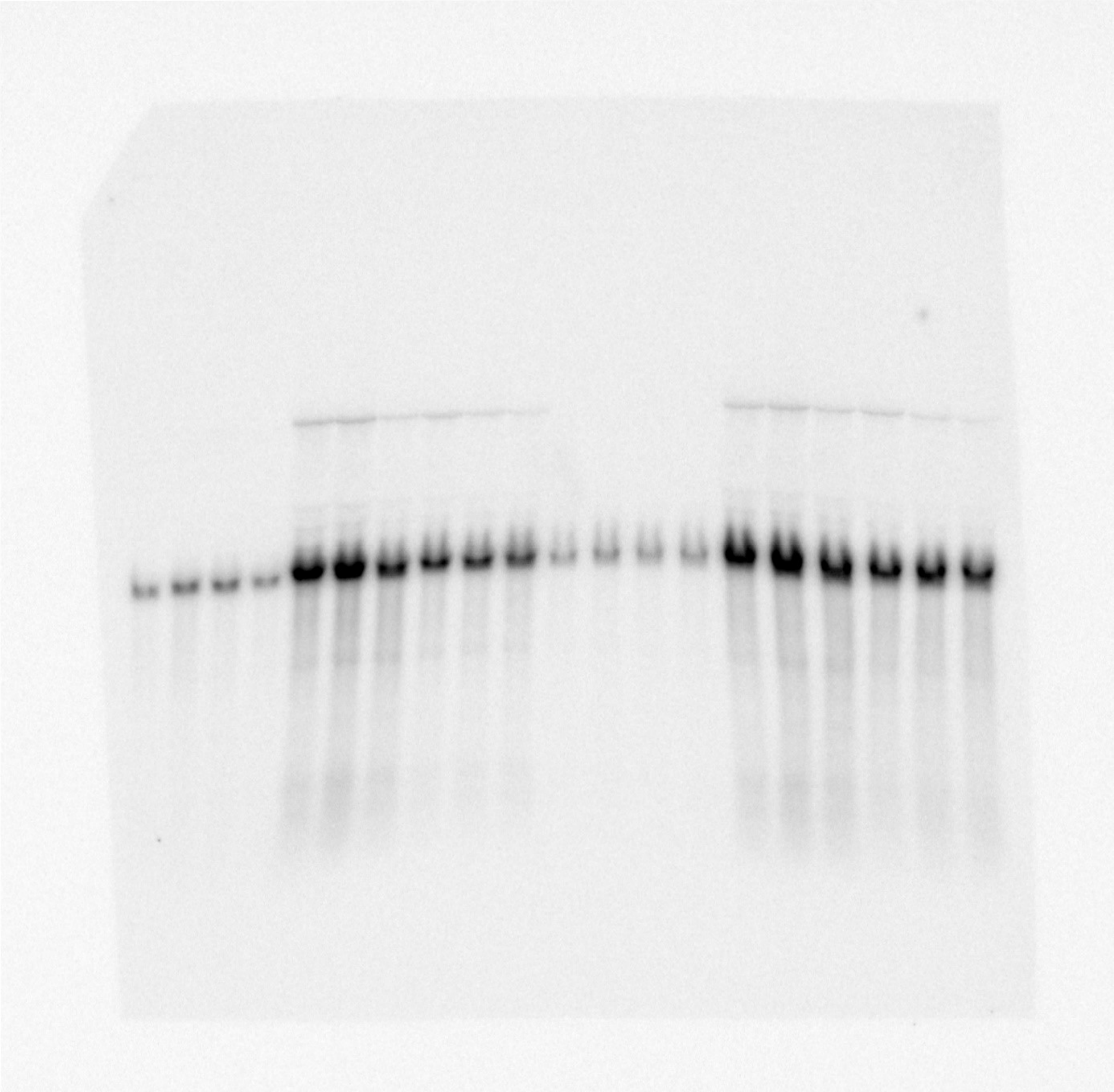

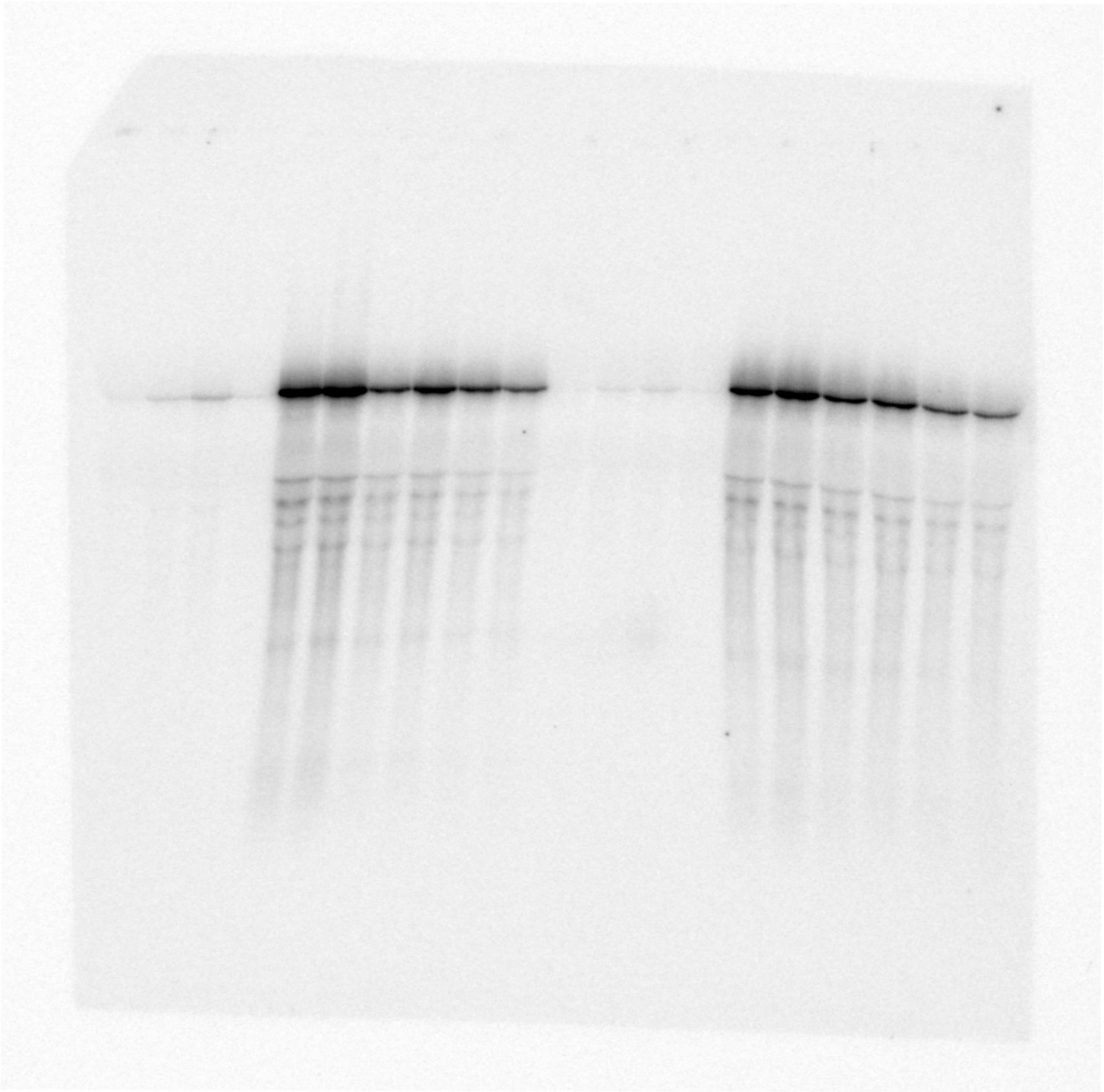

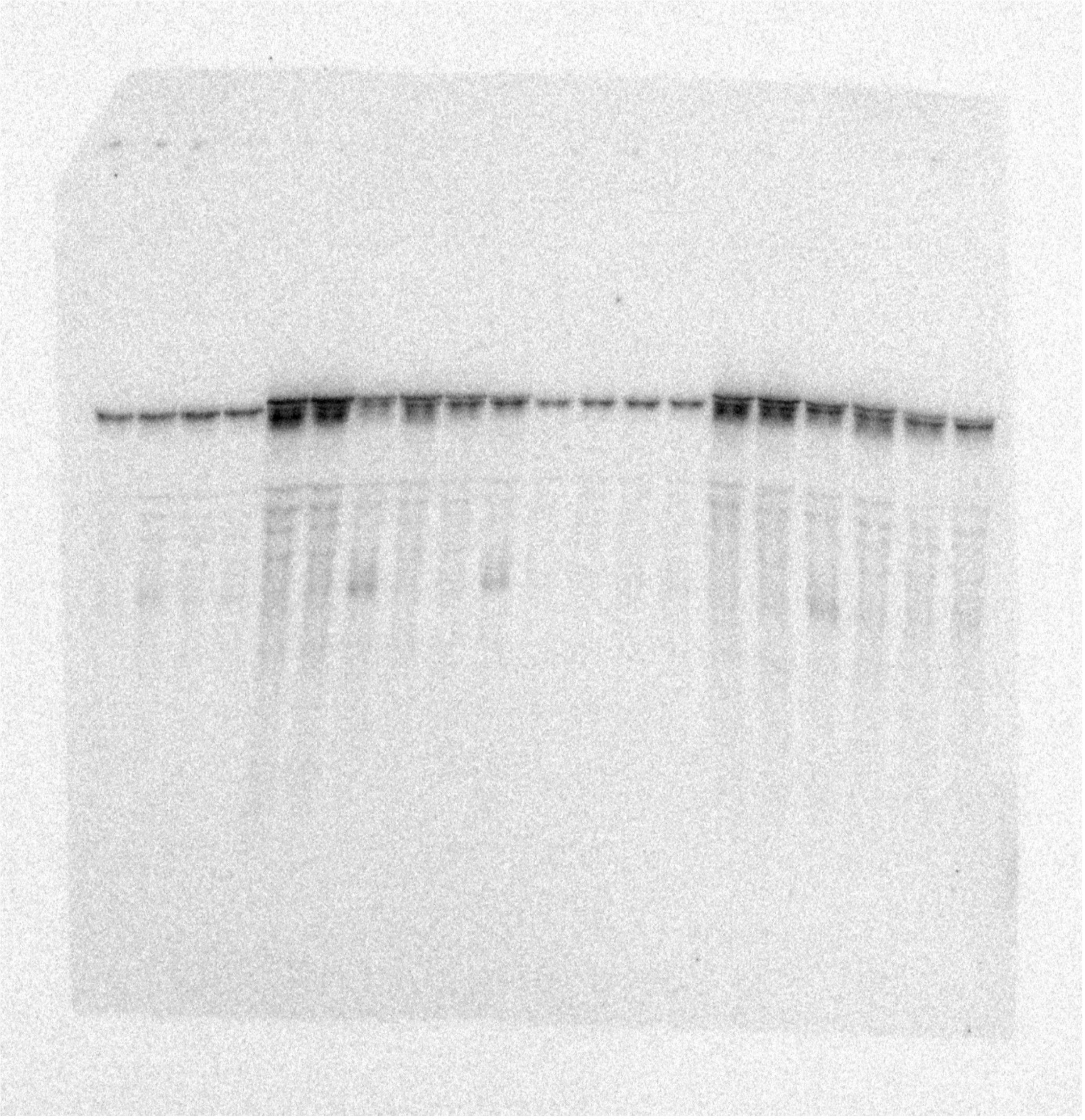

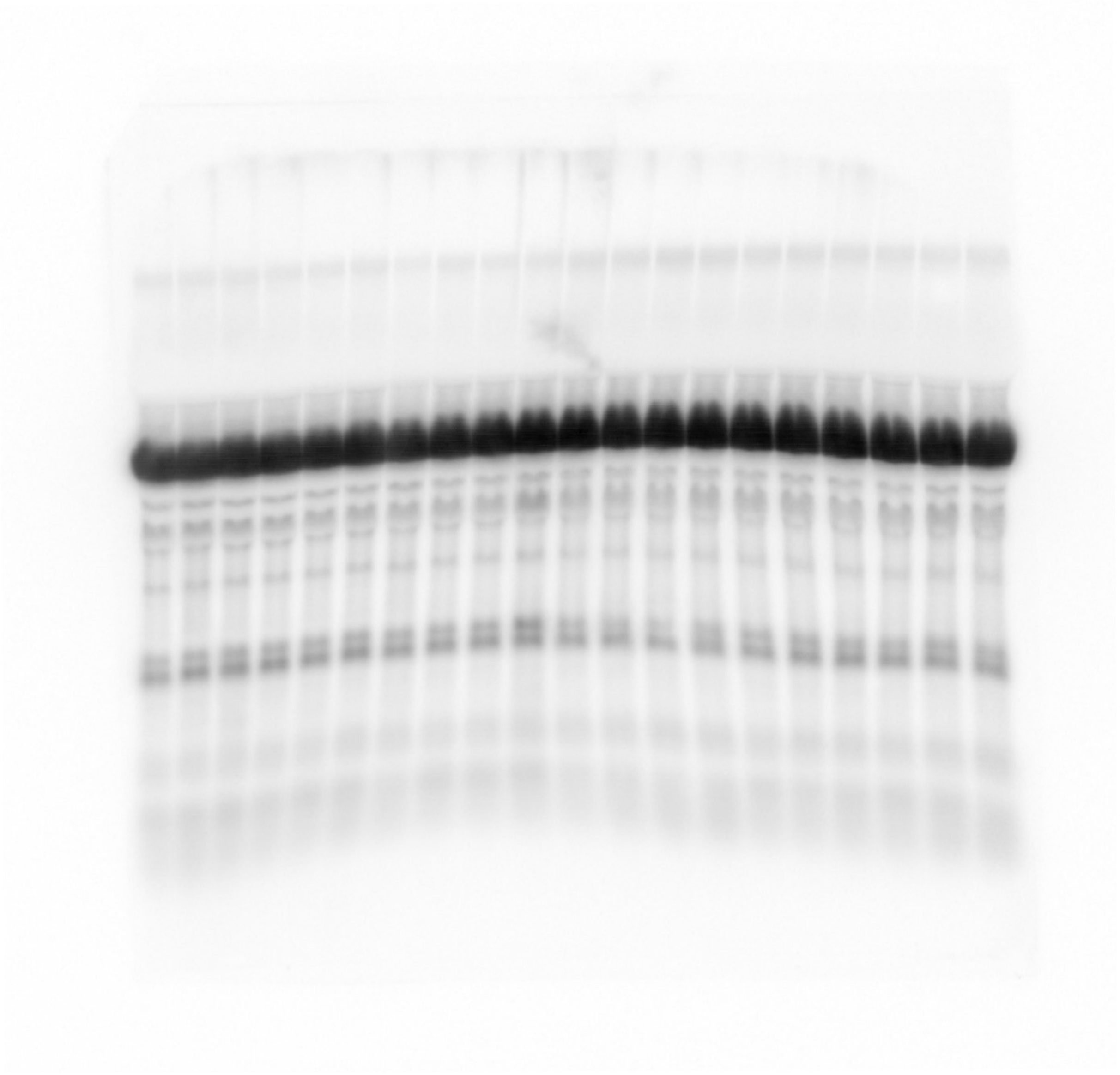

Raw data for Fig. 5A

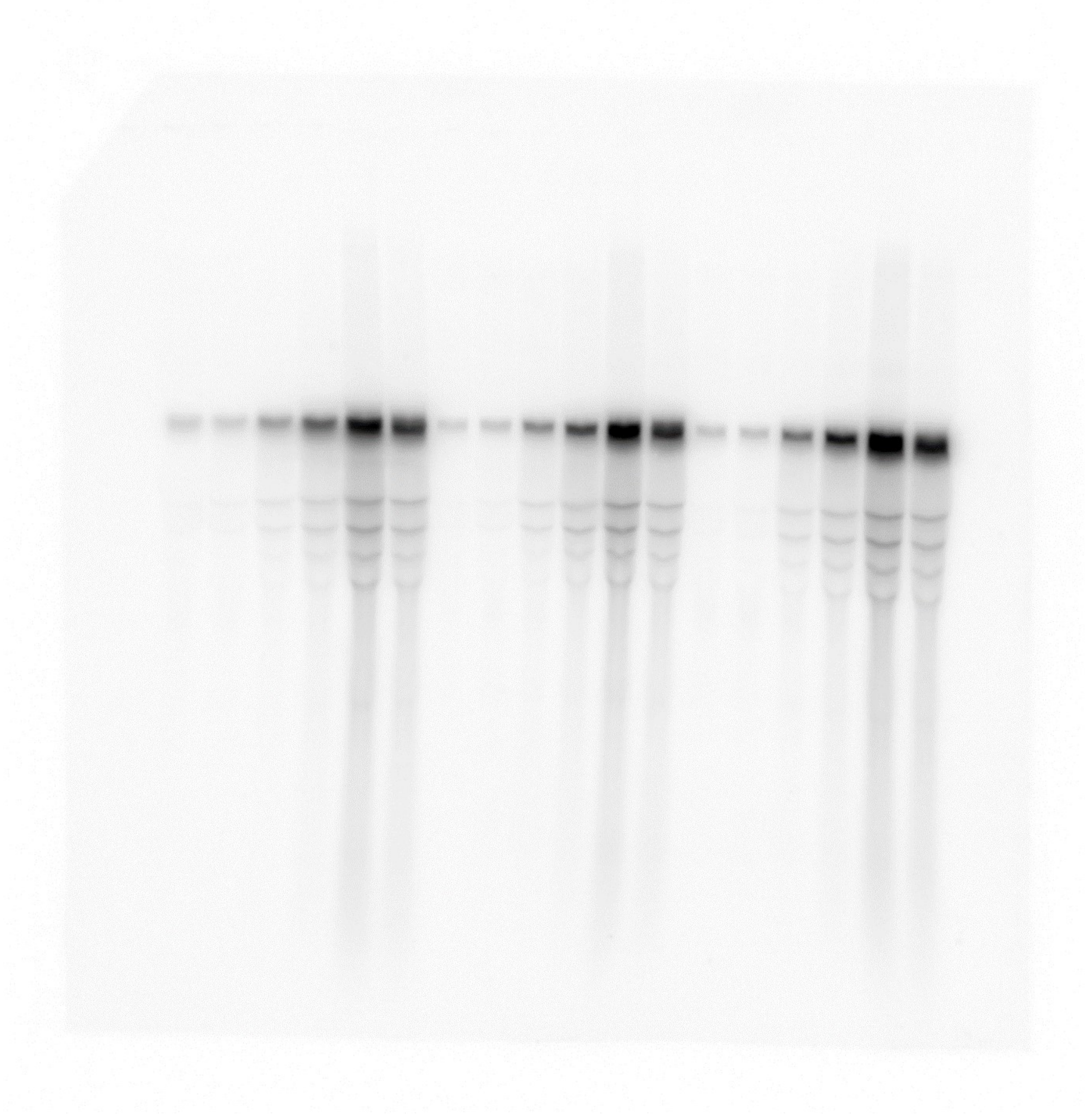

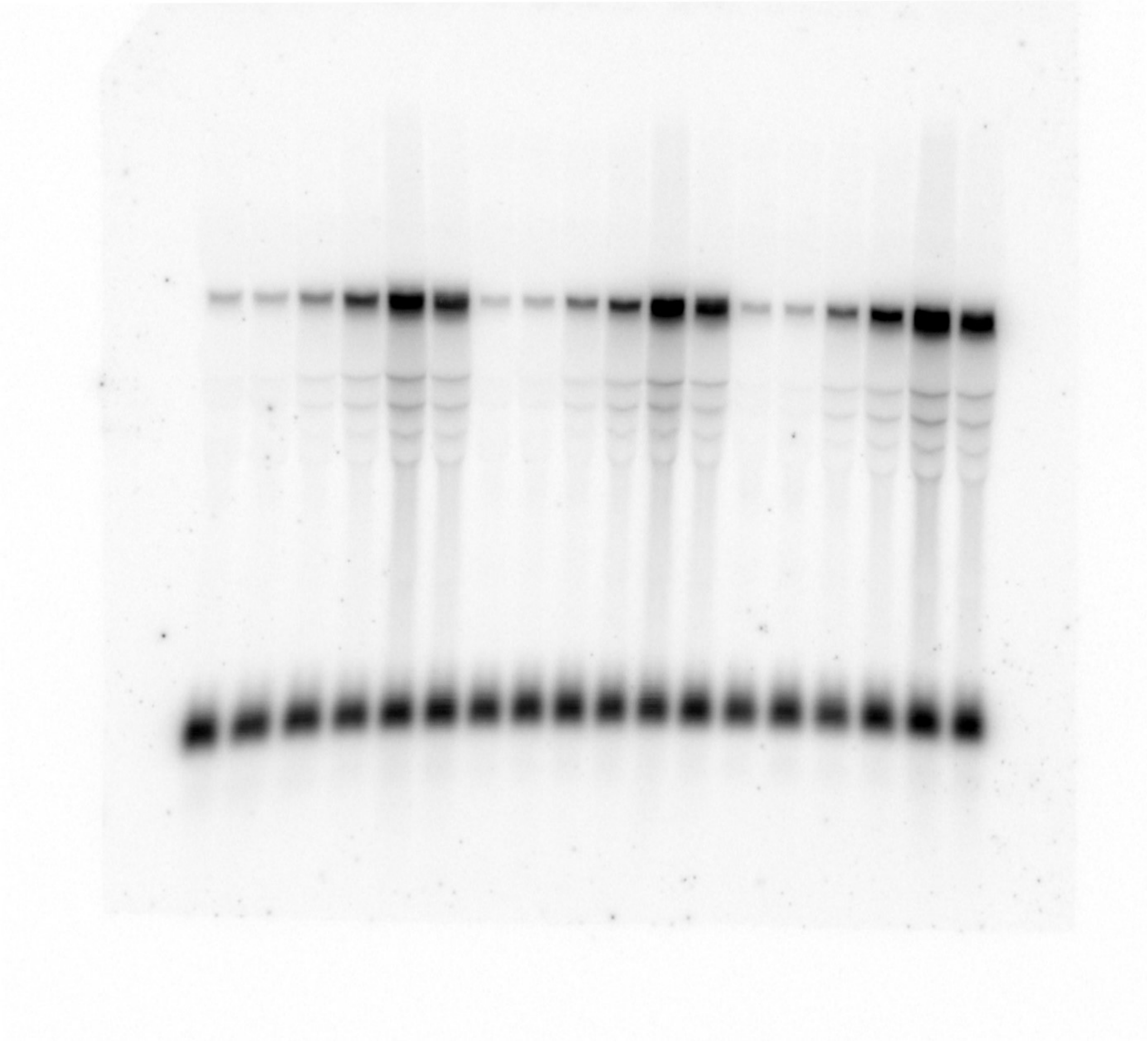

Raw data for Supplementary Fig. S3B

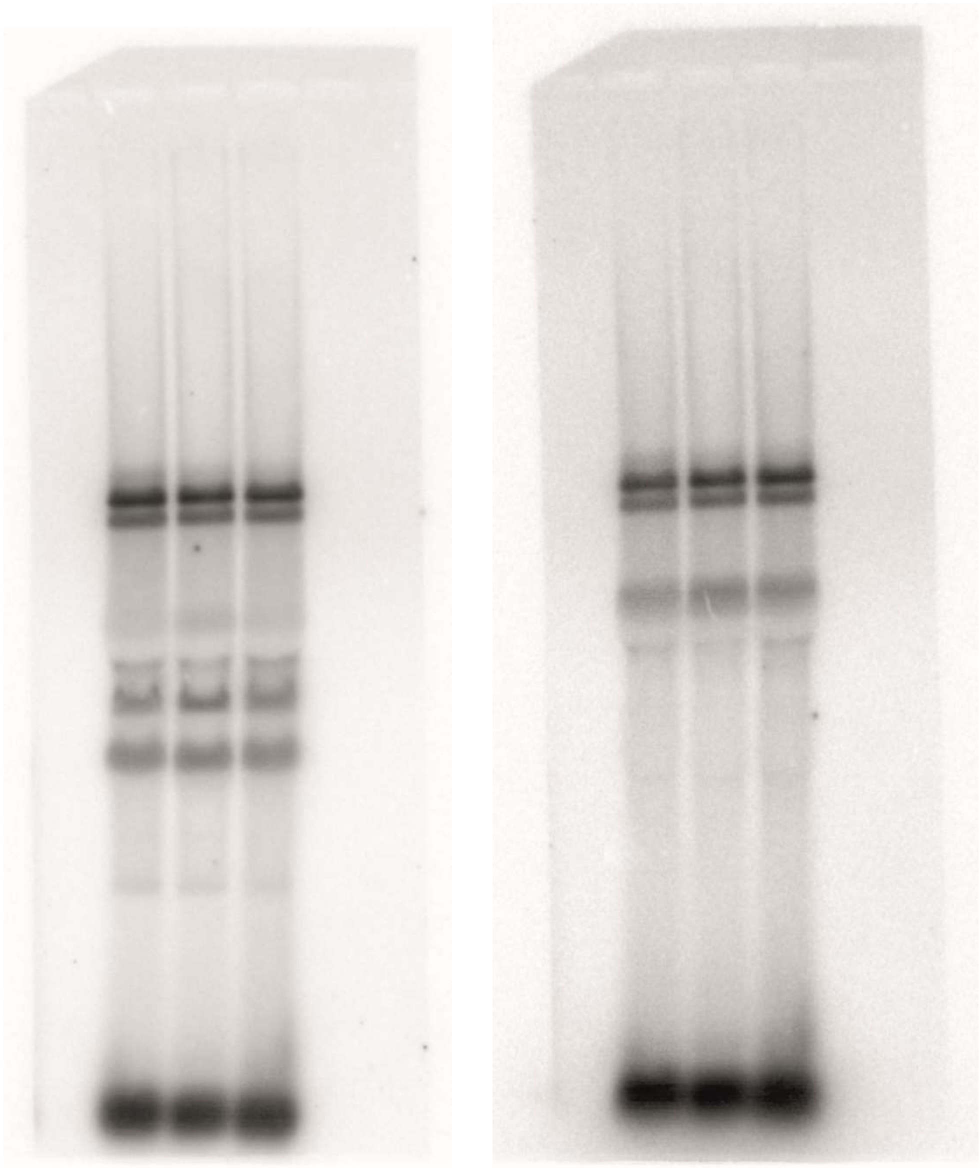

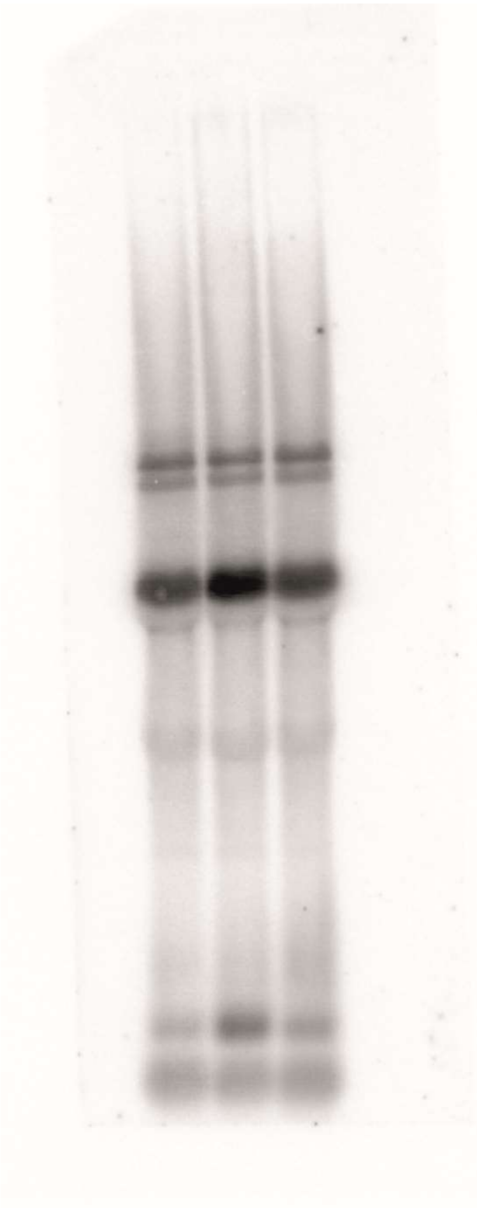

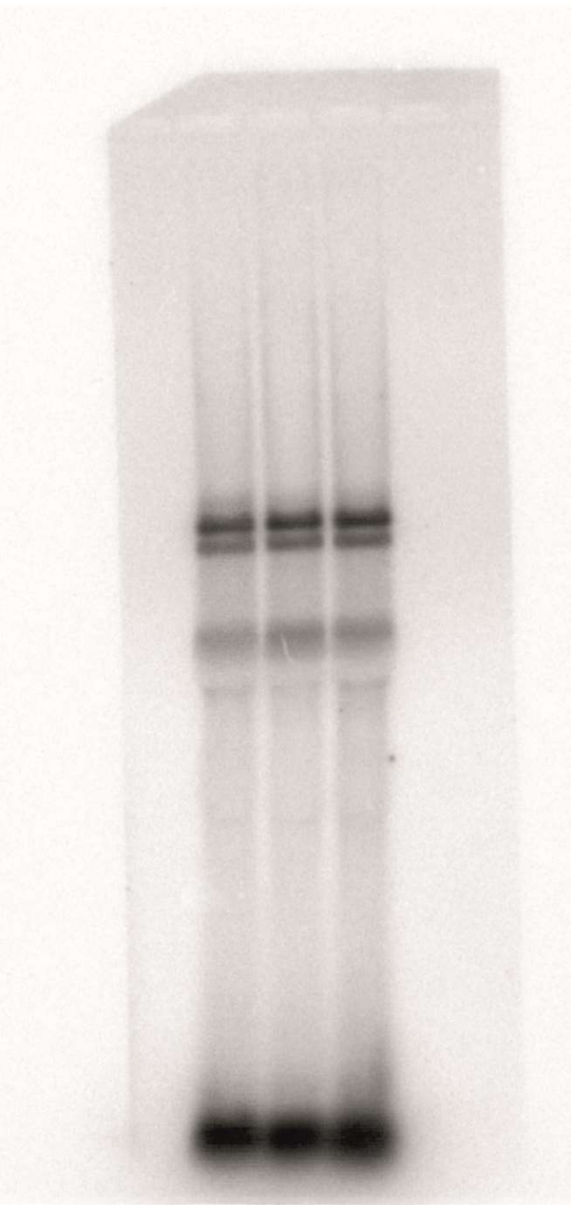

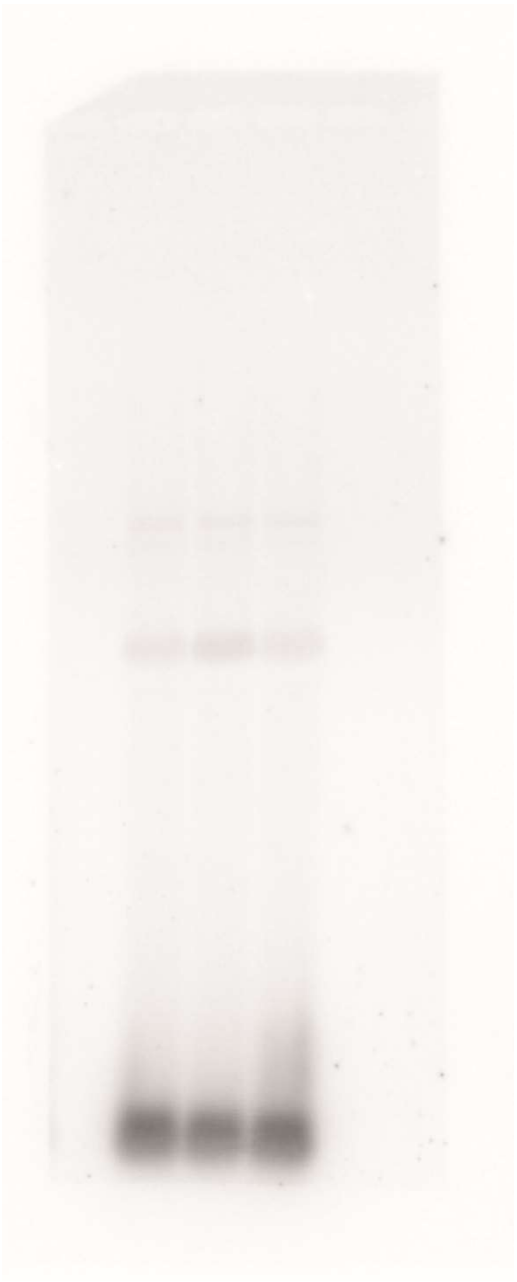

Raw data for Supplementary Fig. S3C

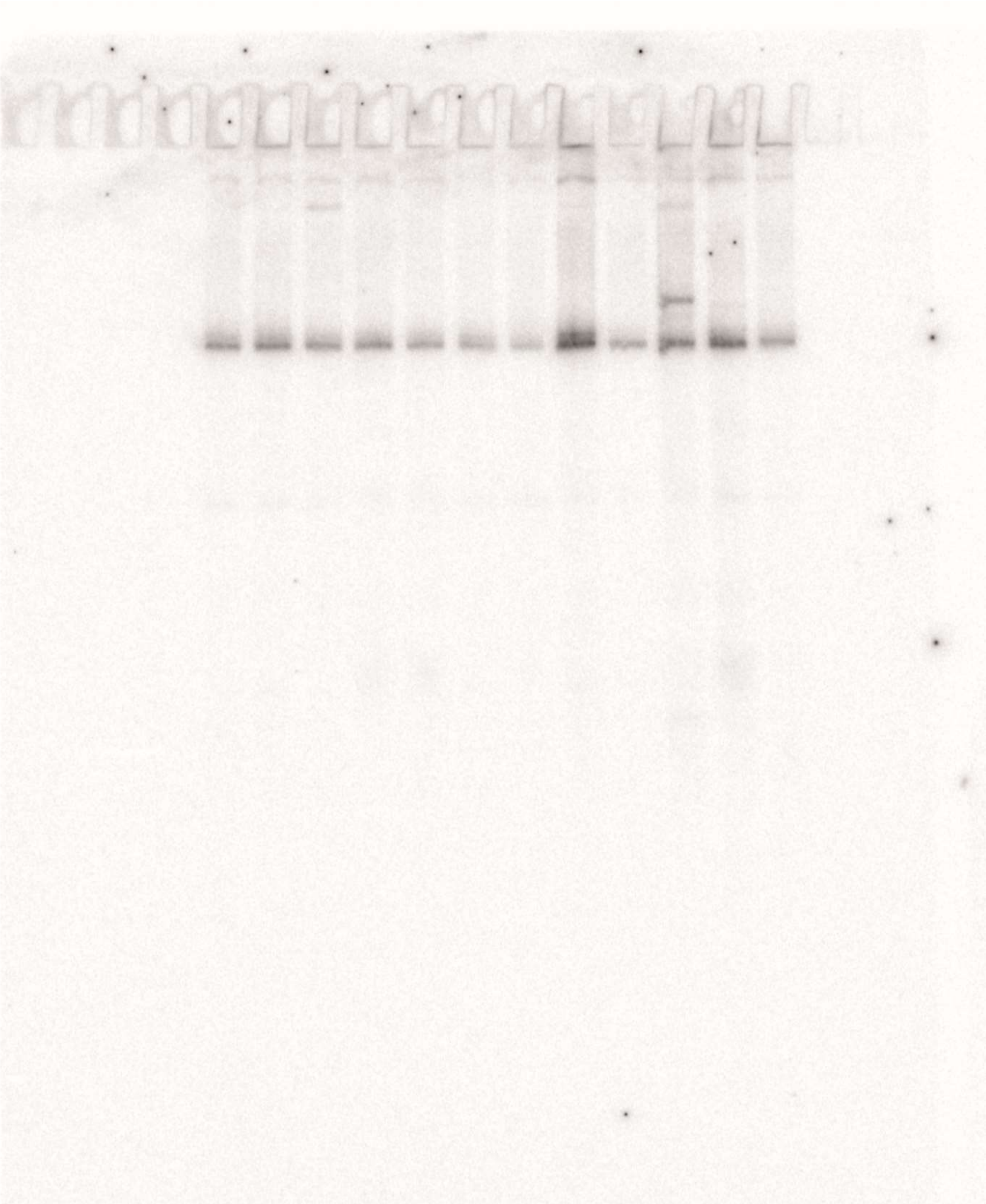

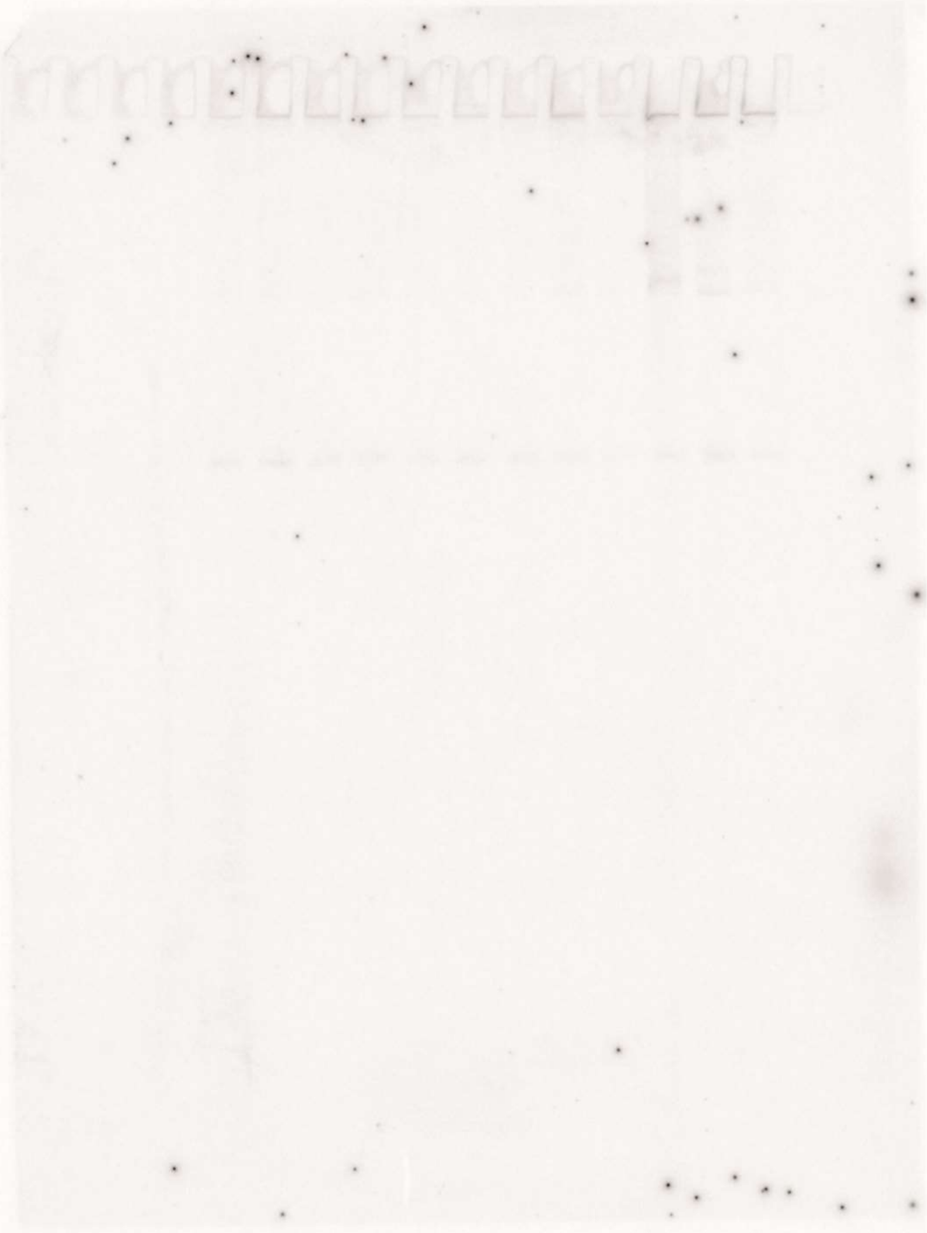

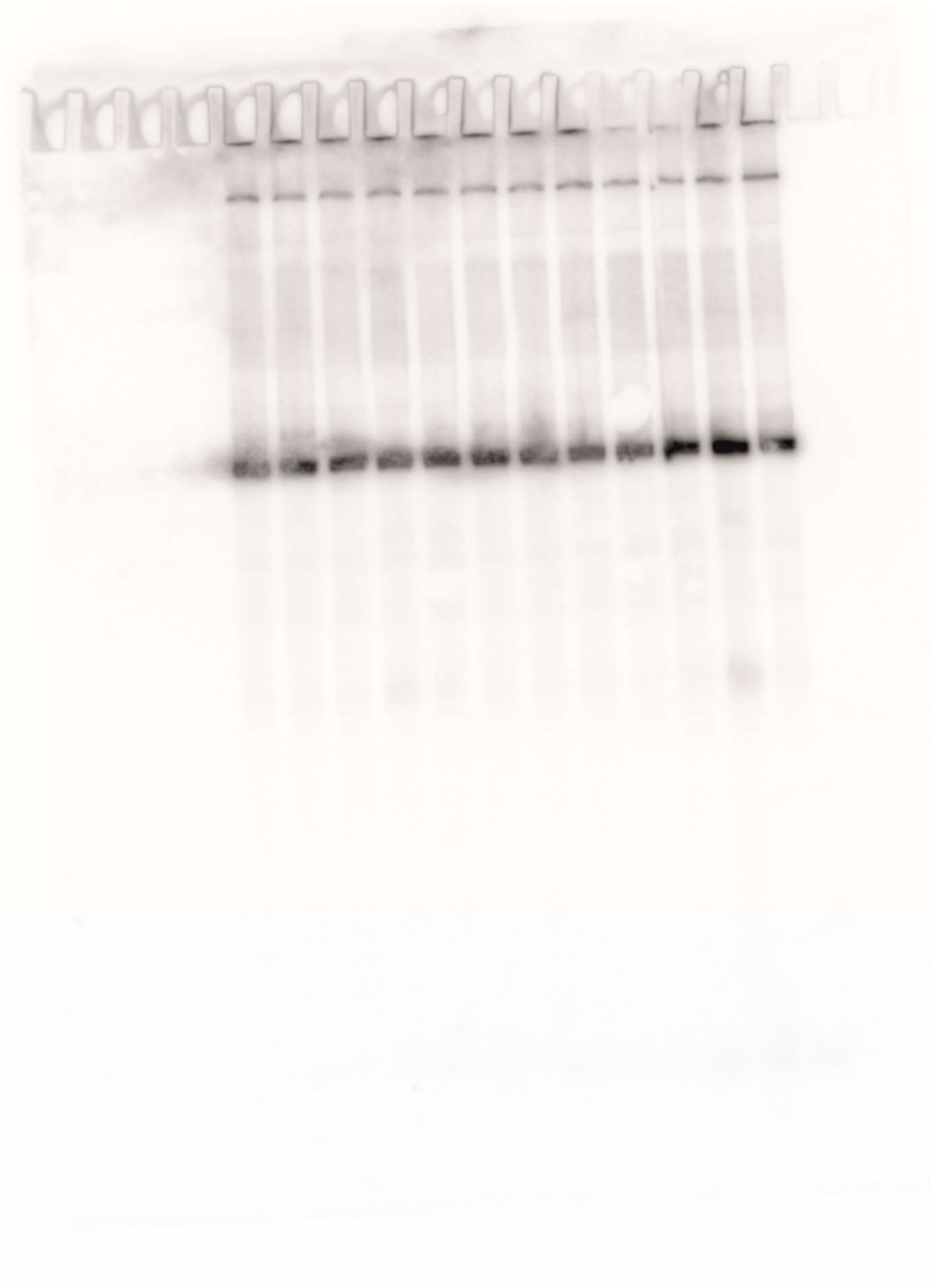

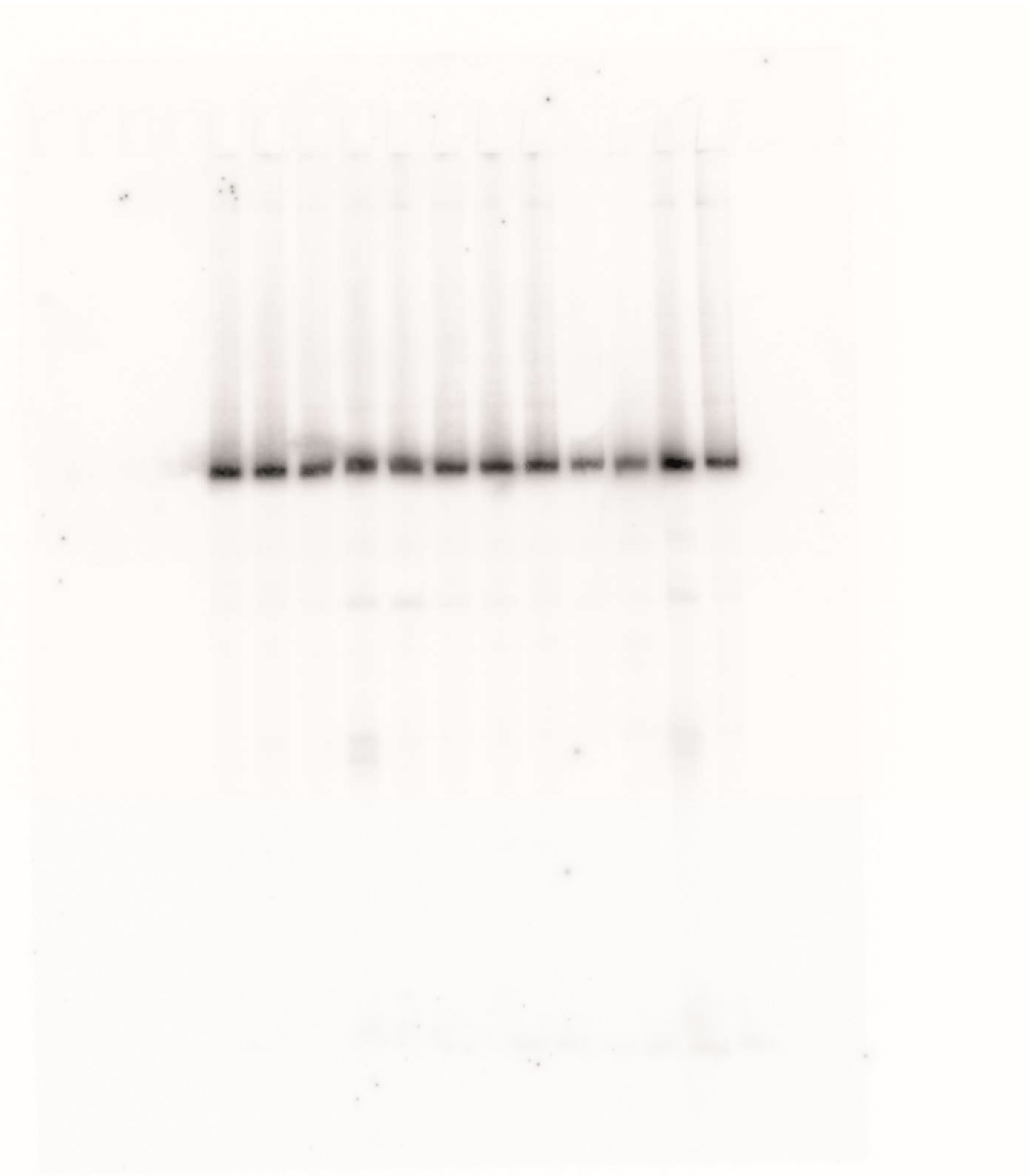

Raw data for Supplementary Fig. S3D

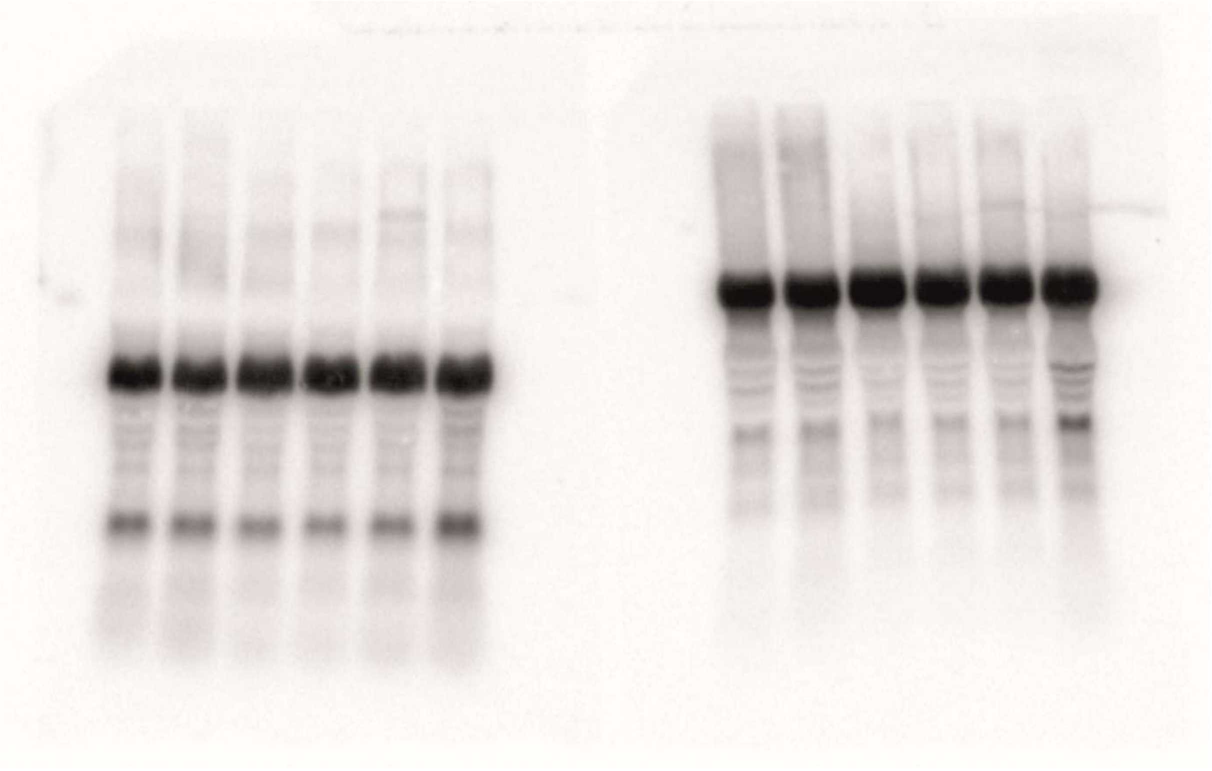

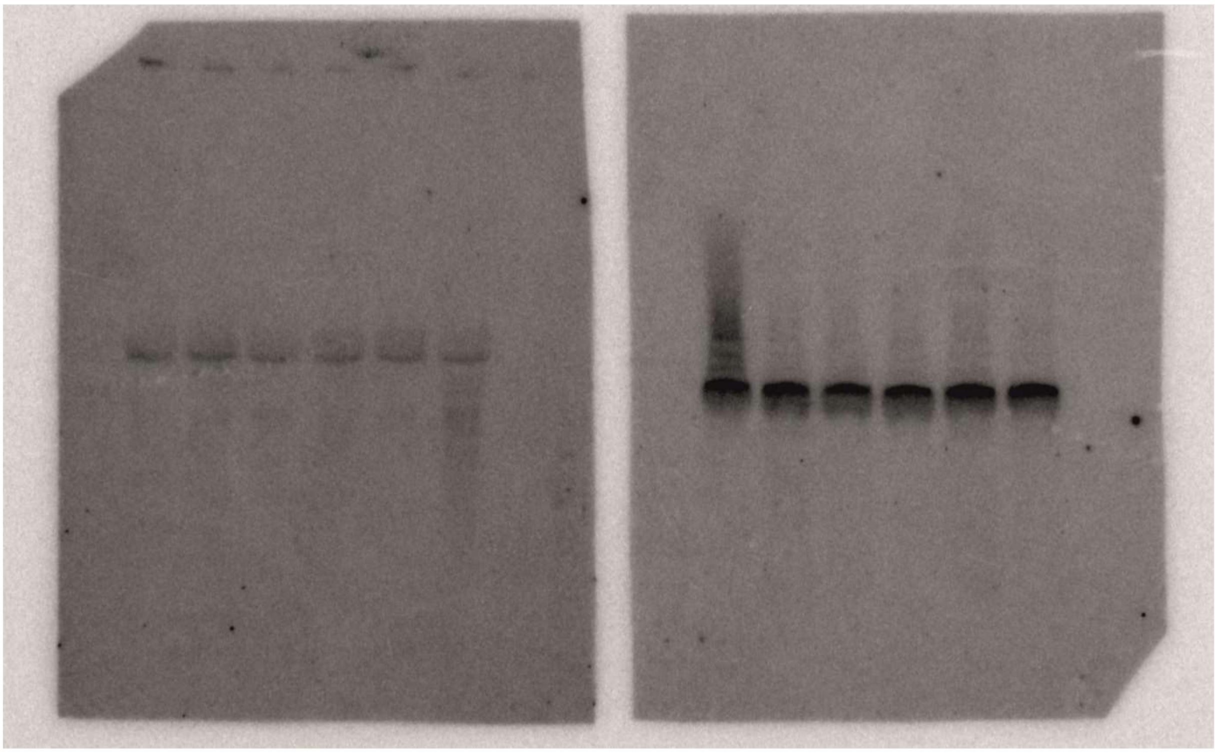

Raw data for Supplementary Fig. S4A

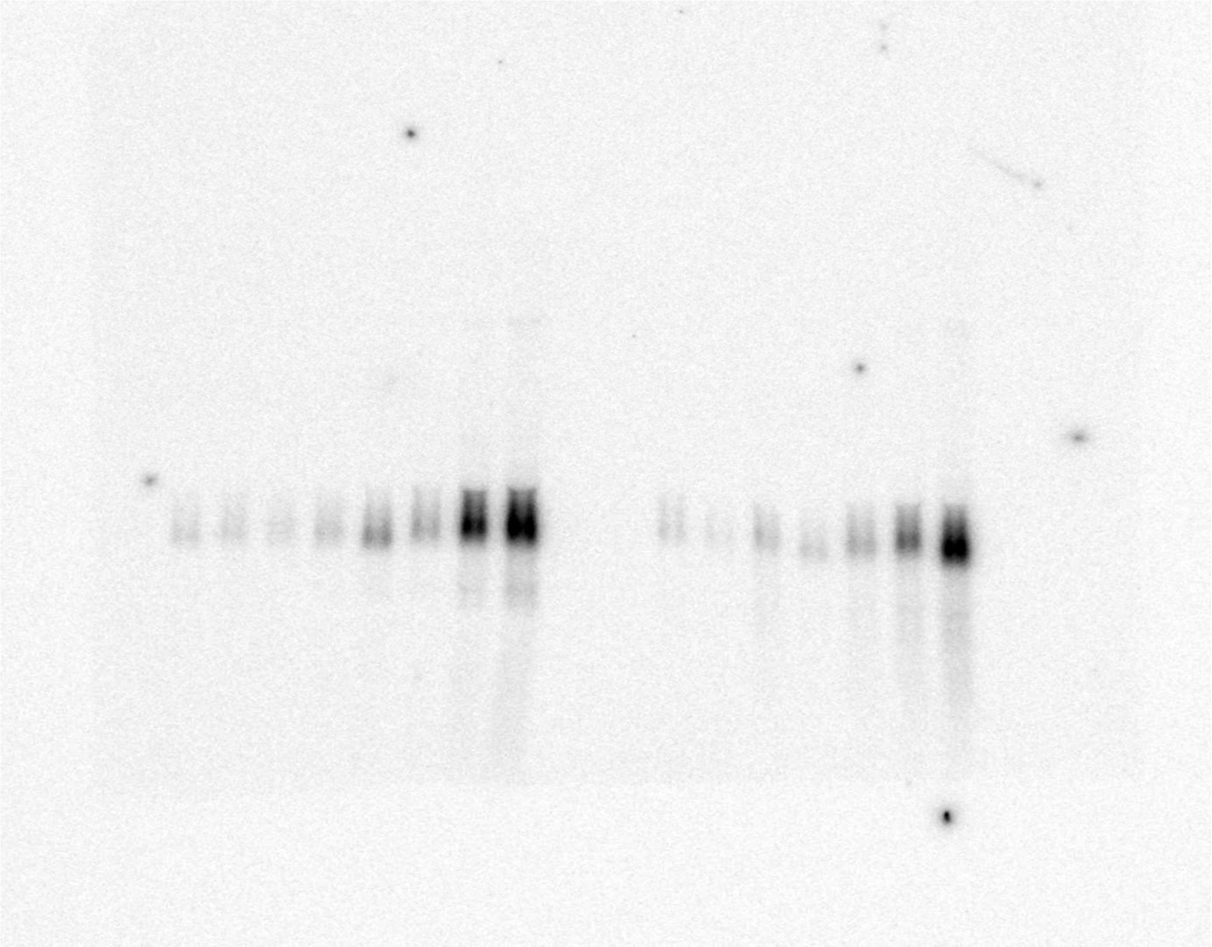

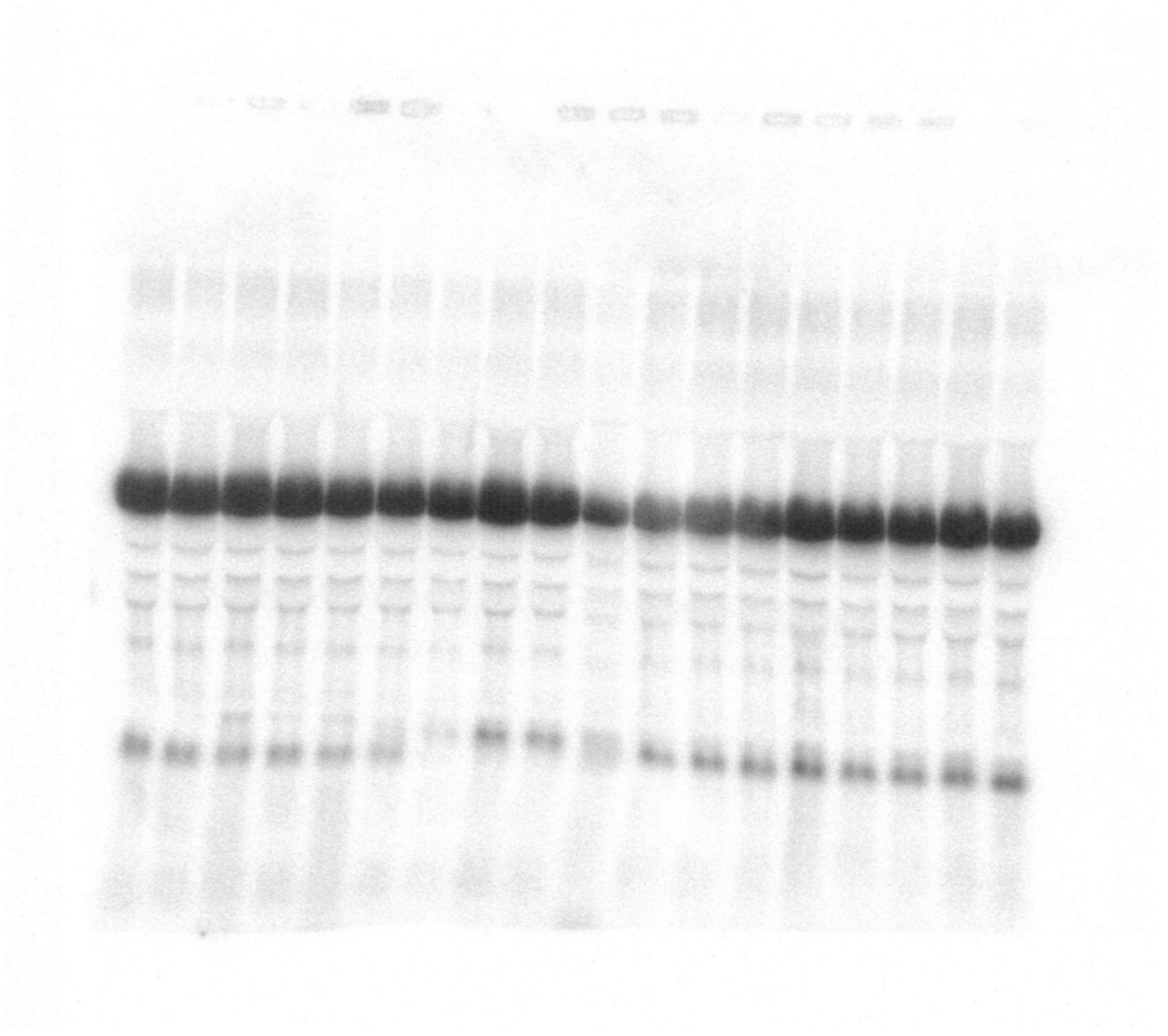

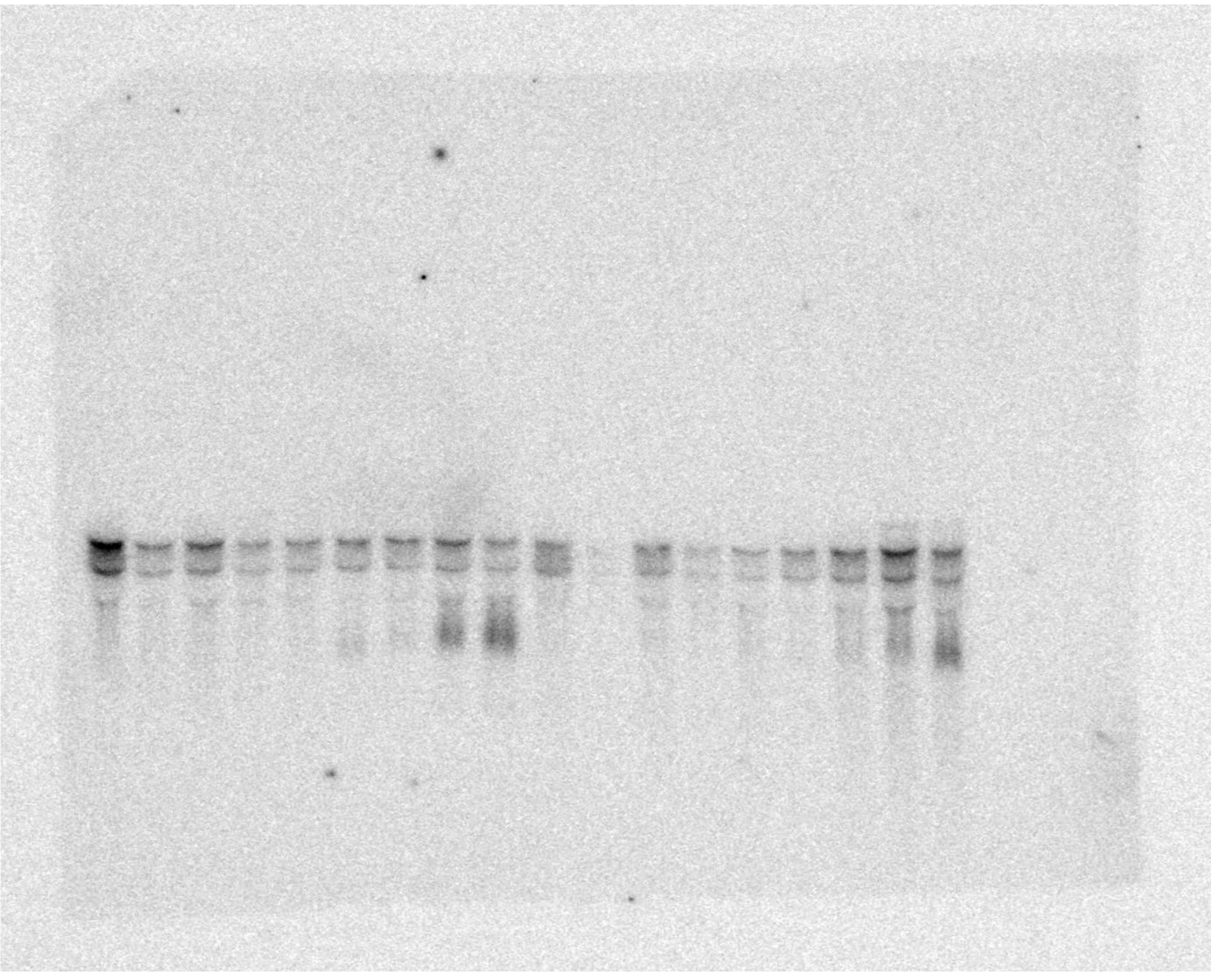

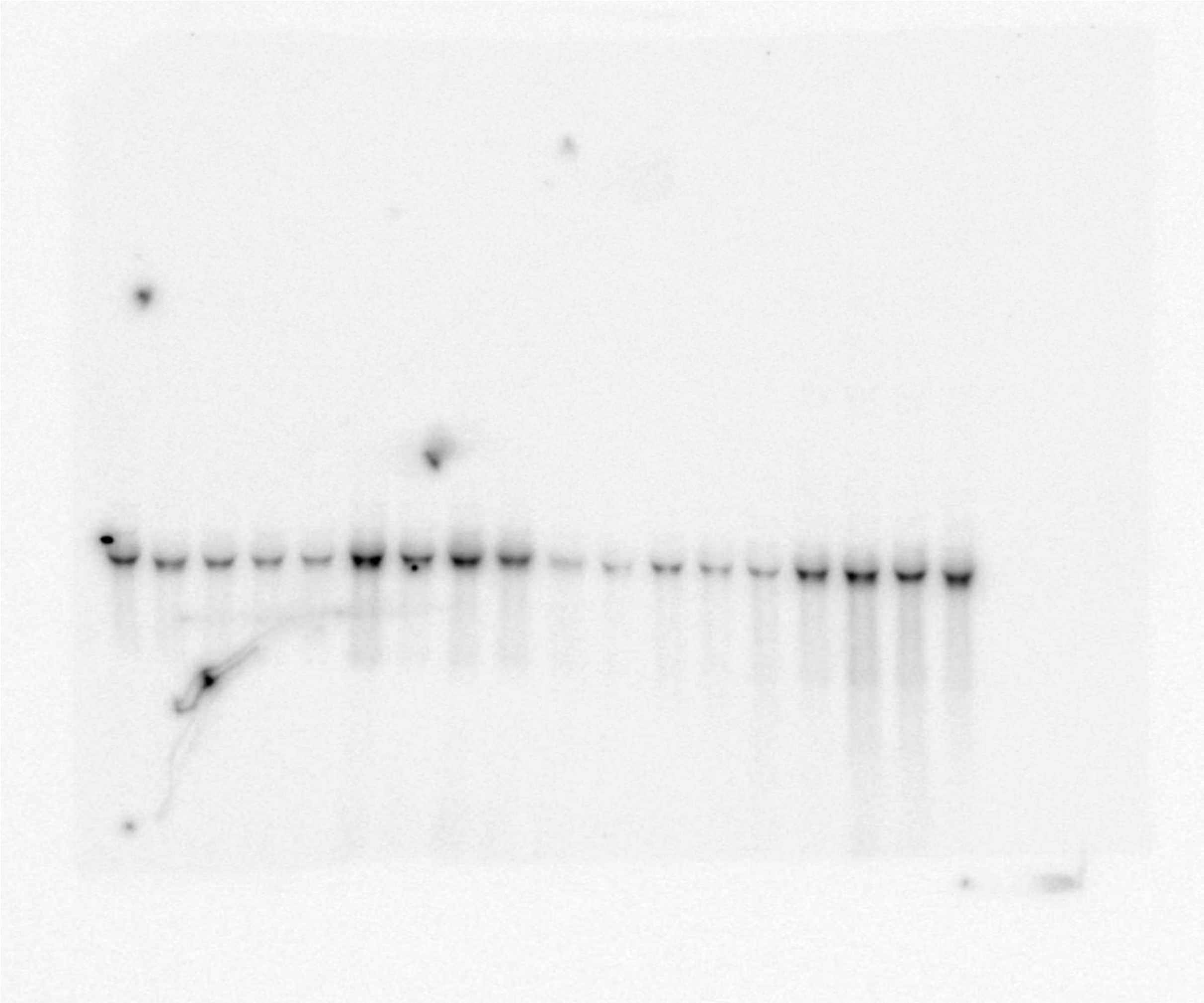

Raw data for Supplementary Fig. S4B

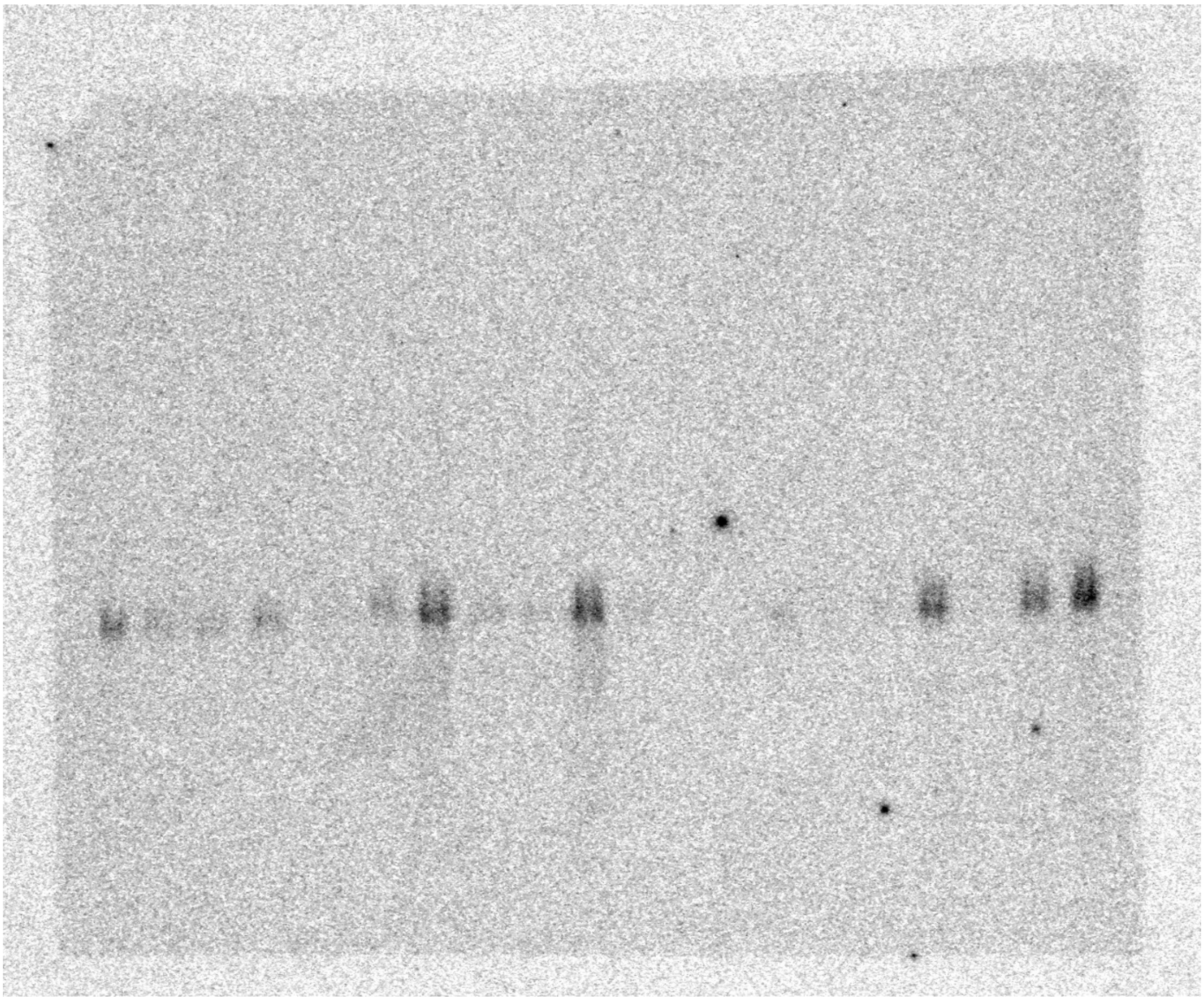

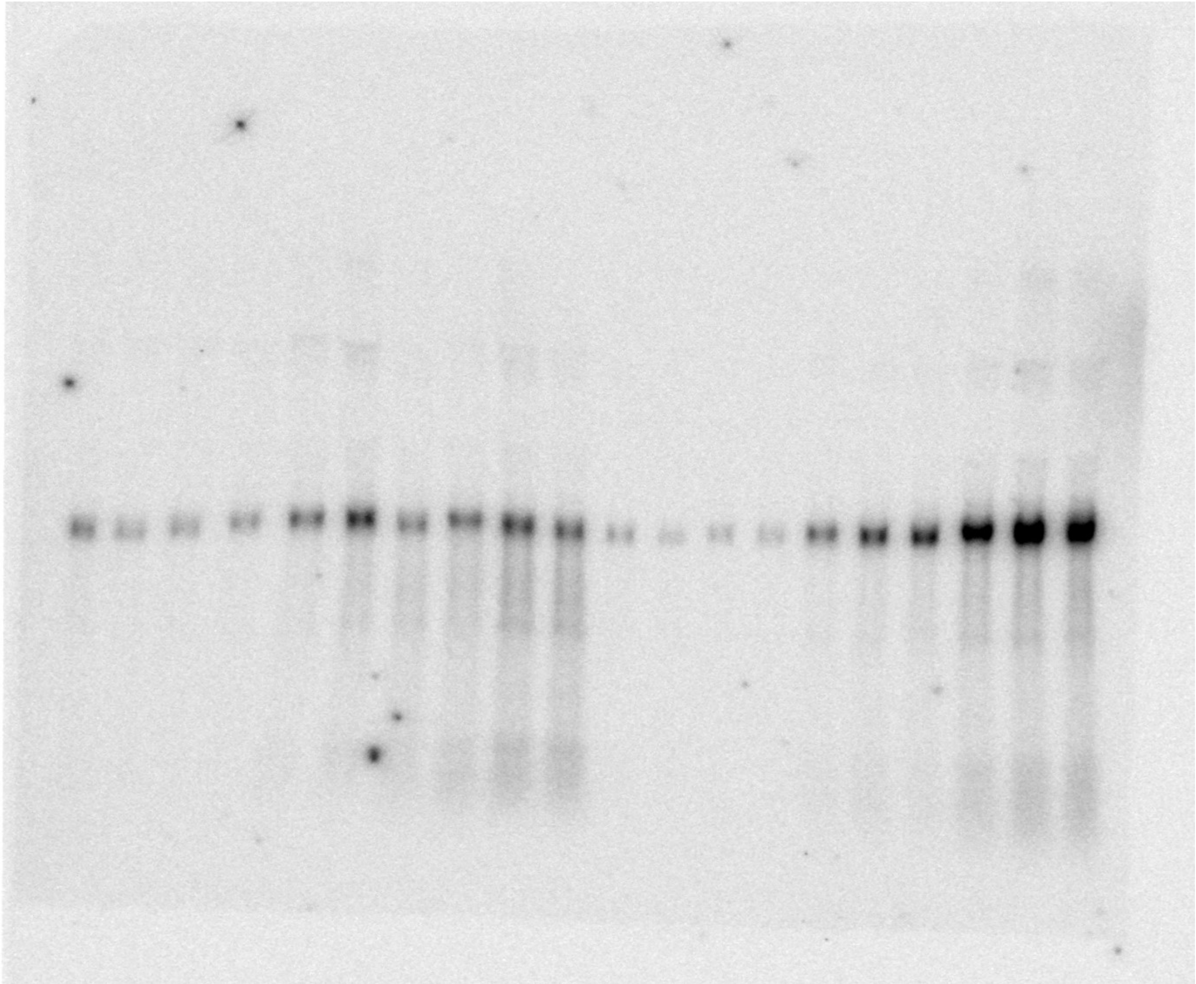

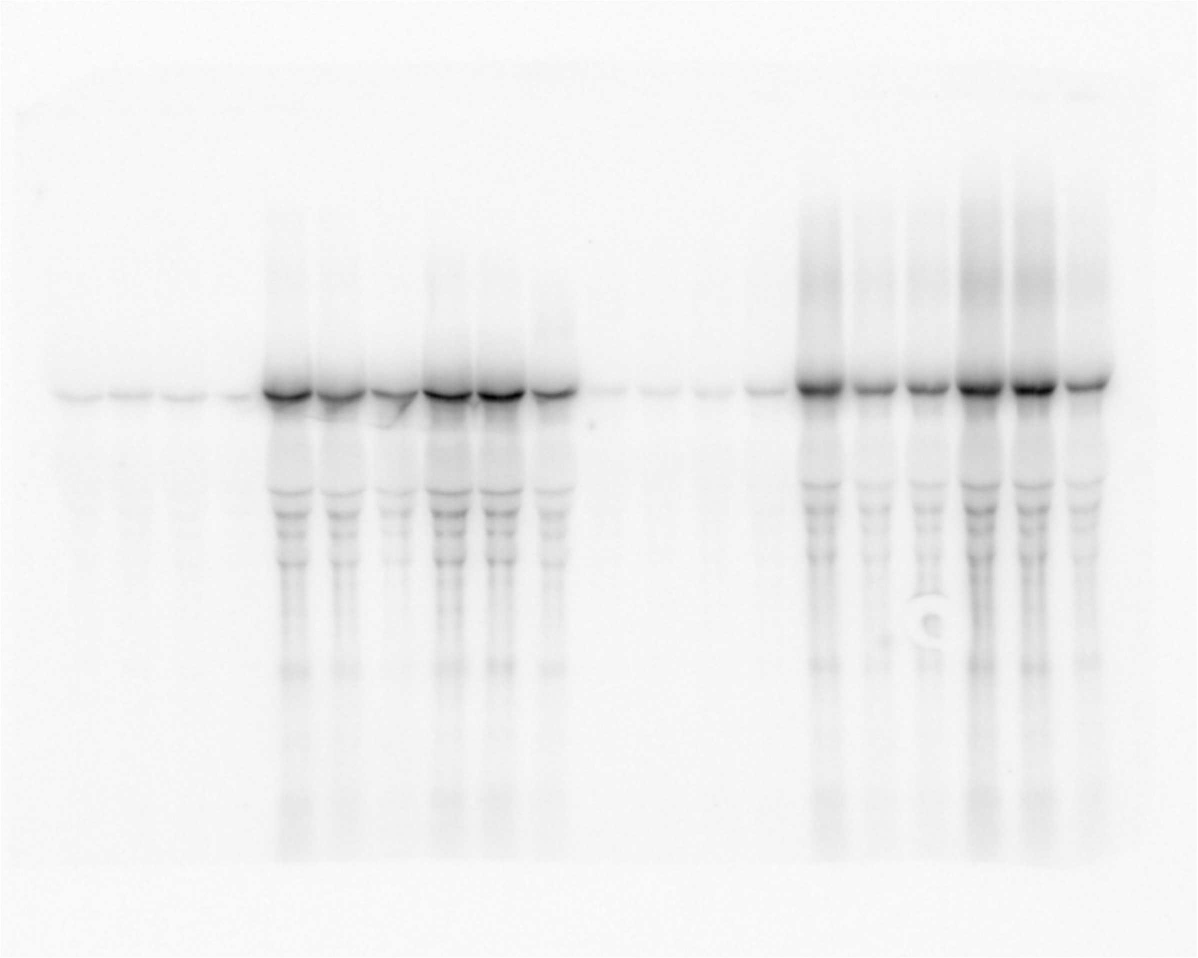

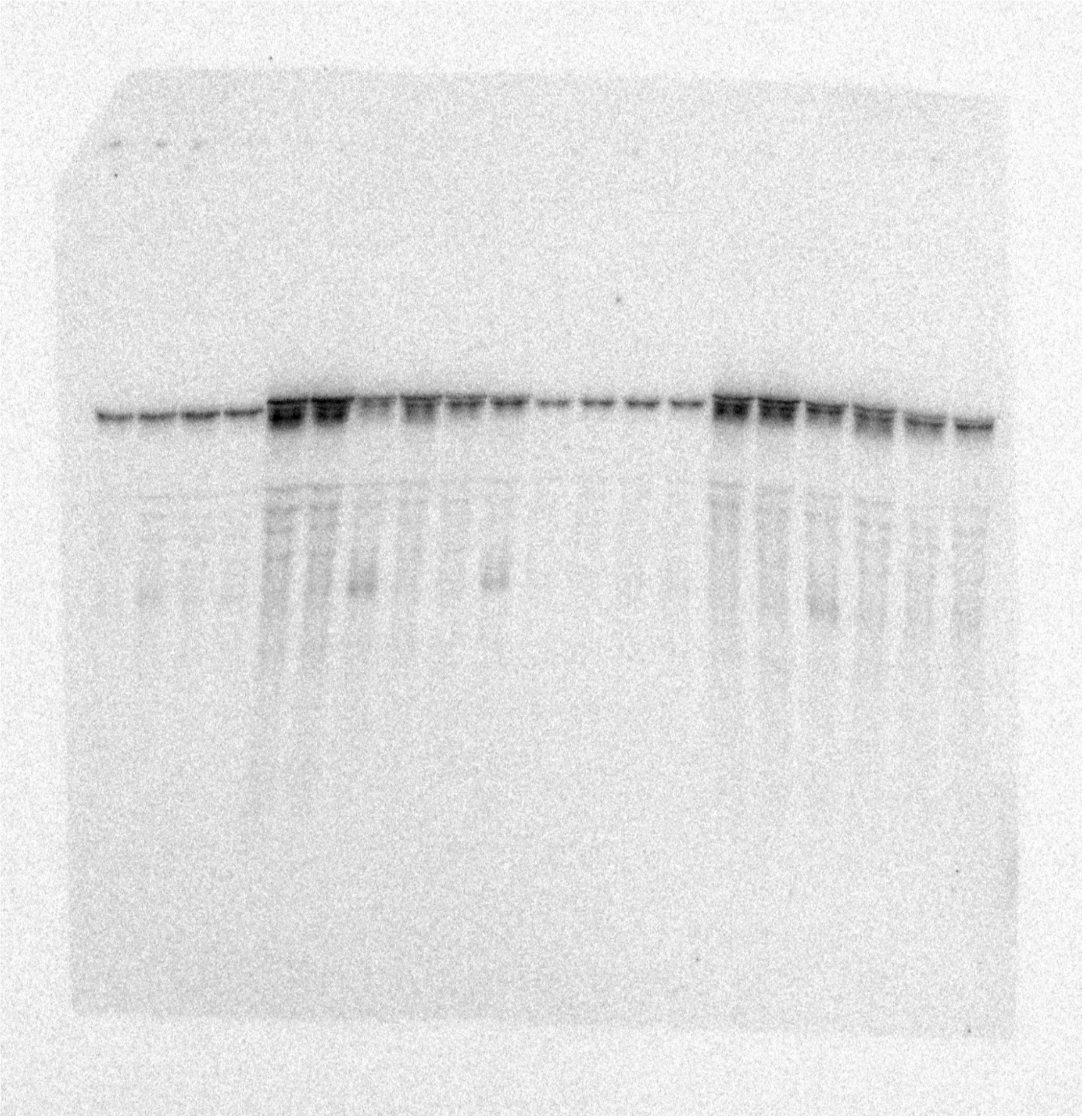

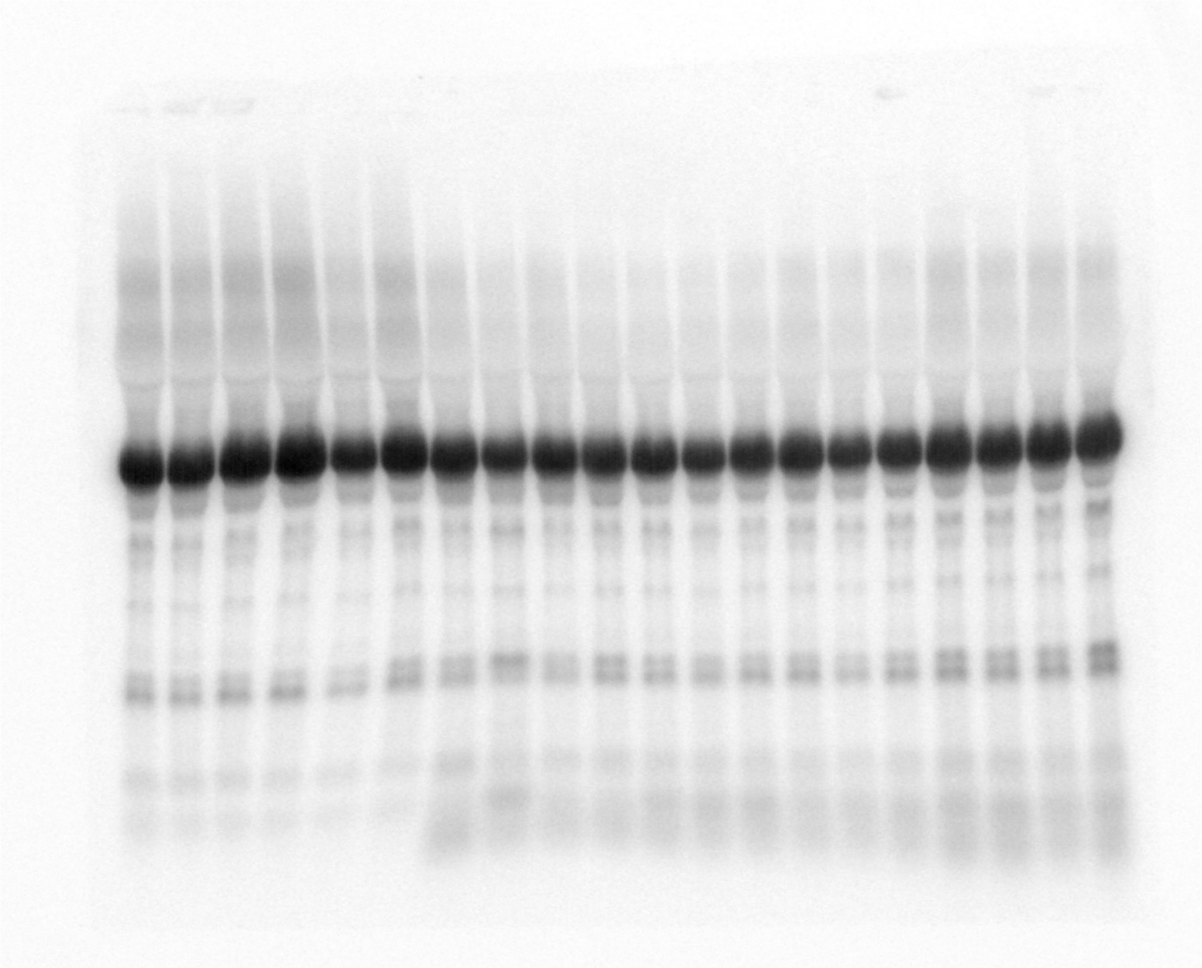

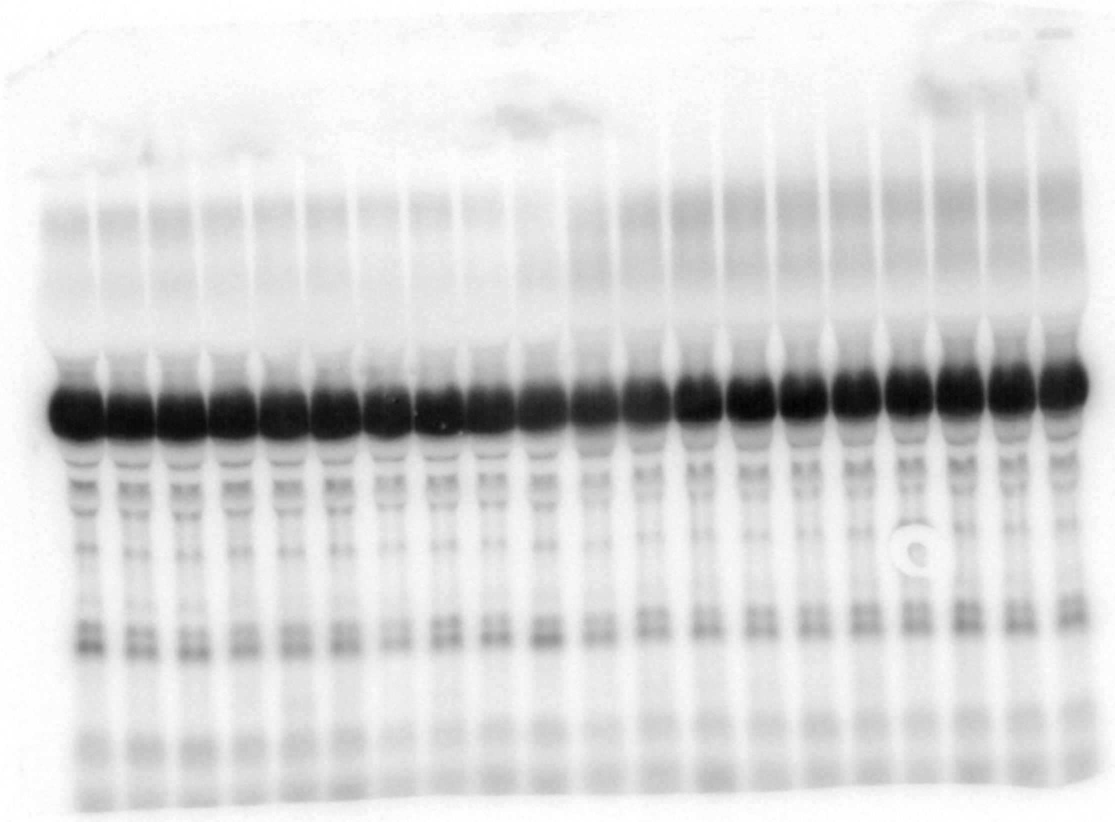

Raw data for Supplementary Fig. S5A

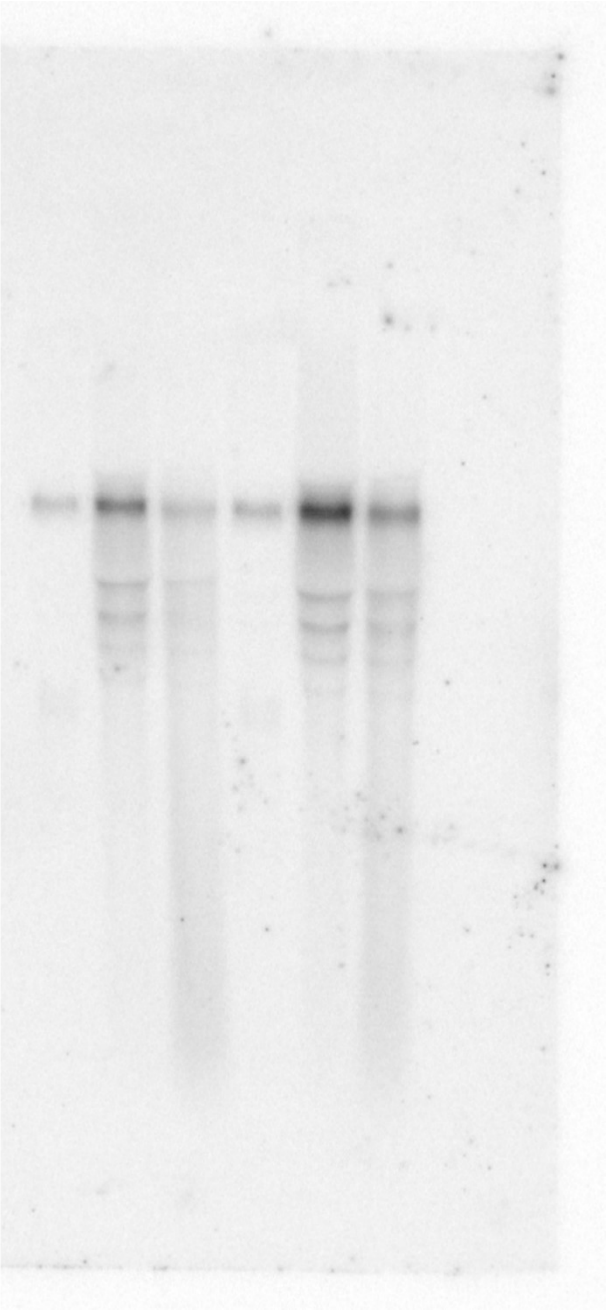

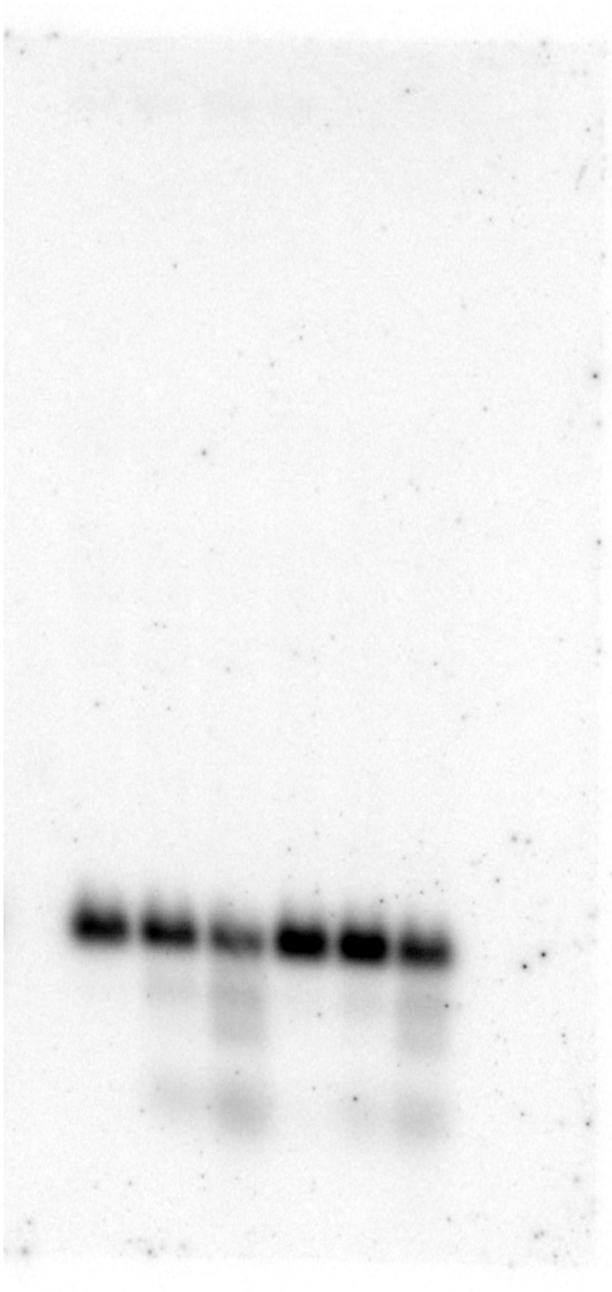

Supplement: Supplementary file 2 — Supplementary Material 2 [file 12870_2023_4561_MOESM2_ESM.pdf]
